# Endoscopic ultrasonography-guided gastroenterostomy versus conventional approaches for cancer-related malignant gastrointestinal outlet obstruction: a meta-analysis with trial sequential analysis

**DOI:** 10.1007/s00464-026-12864-9

**Published:** 2026-05-12

**Authors:** Ana Luíza Rocha Soares Menegat, Brenda Luana Rocha Soares Menegat, Clara Rocha Dantas, Barbara Antonia Dups Talah, Francisco Cezar Aquino de Moraes

**Affiliations:** 1 University of Caxias Do Sul, Caxias Do Sul, 95070-560 Brazil; 2 University of Buenos Aires, Buenos Aires, C1053 Argentina; 3 Pontifical Catholic University of Paraná, Curitiba, 81020-430 Brazil; 4 Federal University of São Paulo, São Paulo, 04023-062 Brazil

**Keywords:** EUS-GE, Surgical gastrojejunostomy, Enteral stenting, Malignant gastric obstruction

## Abstract

**Background:**

Malignant gastric outlet obstruction (MGOO) is a disabling complication of advanced gastrointestinal cancers, accounting for 50–80% of gastric outlet obstruction cases. As patients have unresectable disease, management is palliative and traditionally relies on surgical gastrojejunostomy (SGJ) or enteral stenting, both with limitations. Endoscopic ultrasound-guided gastroenterostomy (EUS-GE) has recently emerged, but its role remains uncertain. We conducted a systematic review and meta-analysis comparing EUS-GE with SGJ and enteral stenting.

**Methods:**

Pubmed, Embase, Scopus, and Cochrane were searched for eligible studies. Mean differences (MDs) and risk ratio (RR) with 95% confidence intervals (CIs) were calculated using a random-effects model. Heterogeneity was examined with *I*^2^ statistics and trial sequential analysis evaluated the cumulative evidence. Prespecified subgroup analyses were conducted for randomized controlled trials and propensity score-matched studies. Statistical analyses were performed using RStudio.

**Results:**

Twenty-two studies were included, comprising 22,211 and 1,479 patients in the EUS-GE vs SGJ and EUS-GE vs enteral stenting analysis, respectively. EUS-GE was associated with fewer infections (RR 0.46; *p* = 0.0137), overall postoperative complications (RR 0.38; *p* < 0.0001), including both major (RR 0.43; *p* = 0.0109) and minor complications (RR 0.35; *p* < 0.0001), and a reduced need for reintervention (RR 0.15; *p* < 0.0001) than SGJ, as well as higher clinical success (RR 1.11; *p* = 0.0003) compared with enteral stenting. SGJ showed higher technical success (RR 0.98; *p* = 0.0003).

**Conclusion:**

EUS-GE offers a safe and effective minimally invasive alternative for MGOO. Its growing evidence base supports its consideration as a preferred option in centers with appropriate expertise.

**Graphical Abstract:**

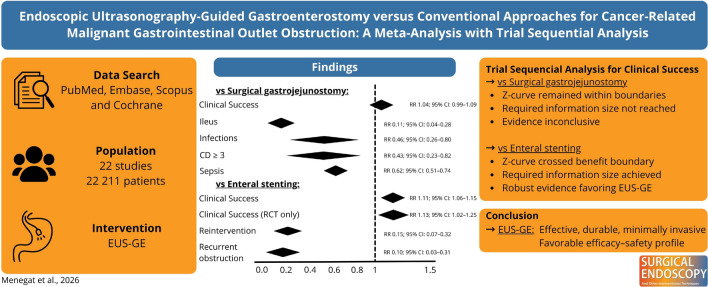

**Supplementary Information:**

The online version contains supplementary material available at 10.1007/s00464-026-12864-9.

Gastrointestinal cancers represent a significant global health burden. Approximately 30–40% of patients with gastric cancer are diagnosed with metastatic disease at the time of presentation [1, 2]. Among patients who develop malignant gastric outlet obstruction, malignancies of the upper gastrointestinal tract account for the majority of cases, with gastric and pancreatic cancers together representing approximately 60–70% of malignant gastric outlet obstruction etiologies, underscoring the clinical relevance of this complication in advanced gastrointestinal malignancies [3, 4].

Malignant gastric outlet obstruction (MGOO) represents a clinically significant complication that markedly impairs quality of life in individuals with gastrointestinal malignancies [5]. Patients with MGOO frequently experience profound reductions in quality of life, driven by symptoms such as nausea, vomiting, abdominal discomfort, and malnutrition. These manifestations are often further aggravated by the systemic and local effects of the underlying malignancy. In clinical cohorts, more than three-quarters of patients are unable to tolerate solid food at presentation, with up to 40% completely unable to maintain oral intake [6]. Management of MGOO often requires invasive interventions and is associated with a substantial burden of adverse events, including stent-related complications, such as re-obstruction, stent migration, bleeding, and perforation, which collectively require repeat endoscopic or surgical procedures in approximately 15–20% of cases [7]. Although curative surgery is feasible only for a small proportion of patients diagnosed at an early stage, palliation of unresectable MGOO can be achieved through surgical gastrojejunostomy (SGJ) or endoscopic placement of a self-expandable metal stent across the obstructed lumen, each approach offering distinct advantages and limitations [8–10].

Historically, among the available palliative options, SGJ was considered the standard approach [11, 12]. SGJ provides durable relief of obstruction-related symptoms and generally requires fewer repeat procedures [13, 14]. Compared with enteral stenting, surgical bypass has consistently been associated with lower rates of re-obstruction and reintervention and longer intestinal patency, outcomes that may translate into improved medium-term survival in selected patient groups (e.g., gastric cancer) [12, 15]. However, these clinical advantages must be balanced against higher perioperative morbidity and a longer hospital stay following surgery, factors that can delay recovery and the timing of systemic therapy [16–18].

With the advent of therapeutic endoscopy, the introduction of self-expanding metal stents (SEMS), first described in 1992, provided a less invasive alternative to surgical bypass and quickly became the preferred first-line palliative option for palliation of MGOO [19, 20]. Enteral stenting provides rapid symptom relief, shorter hospital stays, and faster recovery, making it particularly suitable for patients with limited life expectancy or poor functional status [19, 21, 22]. However, duodenal SEMSs have shown limited long-term durability, with significant rates of stent dysfunction and recurrence of obstructive symptoms (5.40%–42.50%), often necessitating repeat interventions and reducing their effectiveness over time [23–26].

As an alternative, endoscopic ultrasonography-guided gastroenterostomy (EUS-GE) has recently emerged as a promising technique, using EUS to deploy a lumen-apposing metal stent (LAMS) between the stomach and proximal jejunum, a technique that may reduce stent failure by minimizing tumor ingrowth [27–29]. Although EUS-GE is increasingly adopted, a retrospective analysis of the National Inpatient Sample database demonstrated that approximately 20% of the 20,930 hospitalizations for MGOO in the USA were managed with this technique, with the majority of cases still treated surgically [30]. As the global burden of gastrointestinal malignancies continues to increase and the need for effective palliative strategies becomes increasingly urgent, uncertainty persists regarding the optimal therapeutic approach for patients with malignant gastric outlet obstruction [31–33]. To address this ongoing uncertainty, we performed a systematic review and meta-analysis evaluating the efficacy and safety of EUS-GE) compared with SGJ and enteral stenting in adult patients initially diagnosed with gastrointestinal malignancies complicated by malignant gastric outlet obstruction.

## Methods

### Protocol and registration

This systematic review and meta-analysis was conducted following the methodological standards outlined in the Cochrane Handbook for Systematic Reviews of Interventions and reported according to the Preferred Reporting Items for Systematic Reviews and Meta-Analyses (PRISMA) statement (Supplementary Tables 1 and 2) [34, 35]. The study protocol was prospectively registered in the International Prospective Register of Systematic Reviews (PROSPERO; https://www.crd.york.ac.uk/) on October 31, 2025, under the registration number CRD420251180968.

### Eligibility criteria

Studies were considered eligible when they met the following inclusion criteria: (1) enrollment of patients aged ≥ 18 years with MGOO; (2) use of EUS-GE as the intervention; (3) comparison with either SGJ or enteral stenting, allowing analysis of two predefined contrasts (EUS-GE vs SGJ and EUS-GE vs enteral stent); (4) reporting of at least one outcome of interest; and (5) study design classified as a randomized controlled trial (RCT), prospective cohort study, or retrospective cohort study.

Publications were excluded if they met any of the following criteria: (1) narrative or systematic reviews; (2) case reports; (3) case series; (4) clinical guidelines; (5) editorials; (6) letters or correspondence; (7) conference abstracts without extractable data; (8) protocols without published results; or (9) studies involving overlapping patient cohorts without uniquely identifiable data.

### Search strategy

A comprehensive literature search was performed in PubMed, Embase, Scopus, and the Cochrane Central Register of Controlled Trials (CENTRAL) from database inception through October 2025. Search strategies combined free-text terms related to “gastric outlet obstruction,” “malignant obstruction,” “endoscopic ultrasonography–guided gastroenterostomy,” “EUS-GE,” “surgical gastrojejunostomy,” and “enteral stent,” using Boolean operators (AND, OR) to structure the queries. The complete search strings for each database are provided in Supplementary Table 3.

All records retrieved from the searches were imported into Zotero (version 6.0; Corporation for Digital Scholarship, United States; https://www.zotero.org) for automated and manual deduplication. After deduplication, two independent reviewers (A.L.R.S.M. and B.L.R.S.M.) screened titles and abstracts using Rayyan (Rayyan Systems Inc., Qatar) [36]. Full texts of potentially relevant studies were subsequently assessed for eligibility. Any discrepancies between reviewers were resolved by consensus.

### Data extraction

Data extraction was performed by two reviewers (C.R.D. and B.A.D.T.). Extracted information included first author and year of publication, study design, follow-up duration, country, details of the EUS-GE technique, SGJ technique or enteral stent type, number of patients, sex distribution, age, type of underlying malignancy, and the outcomes reported in each study.

### Quality assessment

Quality assessment of the included studies was performed using the RoB 2 tool for RCTs and the ROBINS-I tool for observational studies. Two reviewers (B.A.D.T. and C.R.D.) evaluated the methodological quality of the studies, and any differences in judgment were resolved by a third reviewer (A.L.R.S.M.). For trials assessed with RoB 2, risk of bias was judged as low, some concerns, or high across the domains of the randomization process, deviations from intended interventions, missing outcome data, outcome measurement, and selection of reported results [37].

Observational studies were appraised with ROBINS-I, which examines potential bias across the domains of confounding, participant selection, intervention classification, deviations from intended interventions, missing data, outcome measurement, and selection of reported results [38]. Each study was categorized as having a low, moderate, serious, or critical risk of bias.

### Sensitivity analysis

To assess the stability of the pooled estimates, we performed a leave-one-out sensitivity analysis, in which each study was removed sequentially and the meta-analysis was recalculated [39]. This procedure helps determine whether any individual study exerts a disproportionate influence on the summary effect or contributes meaningfully to between-study variability. In addition, Baujat plots were constructed to visually depict each study’s contribution to the overall heterogeneity (*Q* statistic) and its leverage on the pooled estimate, enabling the identification of studies with a potentially outsized impact on the results [40]. Only outcomes demonstrating at least moderate heterogeneity (*I*^2^ > 25%) were subjected to these analyses.

### Assessment of publication bias

Assessment of publication bias was performed exclusively for the primary outcomes of technical and clinical success. Funnel plots were constructed for these pooled analyses and visually inspected to evaluate symmetry, with marked asymmetry considered suggestive of potential publication bias, small-study effects, or selective reporting [41]. In addition, Egger’s regression test was applied to statistically assess funnel plot asymmetry, with significant deviation from symmetry interpreted as evidence of small-study effects [42].

### Endpoints and subgroup analysis

For the comparison between EUS-GE and SGJ, the primary outcomes of interest included (1) technical success, (2) clinical success, (3) postoperative complications (ileus, infectious complications, sepsis, anastomotic leak, and perforation), (4) Clavien–Dindo complications ≥ grade 3 and < grade 3, (5) postoperative mortality, (6) reintervention, (7) recurrent obstruction, (8) length of hospital stay, (9) operative time, and (10) time to oral intake.

For the comparison between EUS-GE and enteral stenting, the primary outcomes of interest included (1) technical success, (2) clinical success, (3) postoperative complications, (4) 30-day mortality, (5) reintervention, (6) recurrent obstruction, and (7) length of hospital stay.

To further explore the robustness and consistency of the findings, a predefined subgroup analysis was conducted. For each outcome with at least three eligible studies, the meta-analysis will be repeated by restricting the dataset to (1) RCTs and (2) propensity score-matched (PSM) cohort studies. These subgroup analyses were performed independently for the comparisons of EUS-GE versus SGJ and EUS-GE versus enteral stenting, whenever the available evidence permits.

### Statistical analysis

This meta-analysis was conducted within a random-effects framework, given the expected methodological and clinical heterogeneity across studies comparing EUS-GE with SGJ and with enteral stenting. Effect sizes for dichotomous outcomes were synthesized as risk ratios (RRs) with 95% confidence intervals (CIs), while continuous outcomes were pooled as mean differences (MDs) with corresponding 95% CIs. Study weights were calculated using the inverse-variance method and separate pairwise meta-analyses were performed for each comparison.

Between-study heterogeneity was modeled using a random-effects approach with the restricted maximum likelihood (REML) estimator for *τ*^2^ [43]. Confidence intervals followed a predefined strategy: Wald-type CIs were applied when *τ*^2^ = 0, whereas the Hartung–Knapp–Sidik–Jonkman (HKSJ) adjustment was used when *τ*^2^ > 0 and more than two studies contributed to the analysis, ensuring more reliable inference under heterogeneous conditions [44]. Statistical heterogeneity was quantified using Cochran’s *Q* test and the *I*^2^ statistic, with *p* values > 0.10 and *I*^2^ values between 25 and 50% interpreted as indicative of low-to-moderate heterogeneity. When applicable, 95% prediction intervals (PIs) were computed to estimate the expected range of true effects in future settings [34]. All analyses were conducted in R (version 4.4.1; R Foundation for Statistical Computing) using the “meta” (v. 8.2—1) and “metafor” (v. 4.8—0) packages, with data management performed using “readxl” (v. 1.4.5) and “openxlsx” (v. 4.2.8). A *p* value < 0.05 was considered statistically significant [45].

### Trial sequential analysis

Trial sequential analysis (TSA) was performed to assess the robustness of the cumulative evidence and to control for the risks of type I and type II errors associated with repeated significance testing in cumulative meta-analyses. The analysis was conducted using the TSA Viewer (version 0.9.5.10 Beta; Copenhagen Trial Unit, Center for Clinical Intervention Research, Rigshospitalet, Copenhagen, Denmark) [46]. A random-effects model with the DerSimonian–Laird estimator was used, as implemented in the software. A two-sided type I error (*α*) of 5% and a statistical power of 80% were assumed. The required information size was calculated using an information axis based on sample size, according to the event proportion observed in the control group and the anticipated intervention effect derived from the pooled relative risk. Trial sequential monitoring boundaries for benefit, harm, and futility were constructed using the O’Brien–Fleming alpha-spending function.

## Results

### Study selection and baseline characteristics

**Fig. 1:**
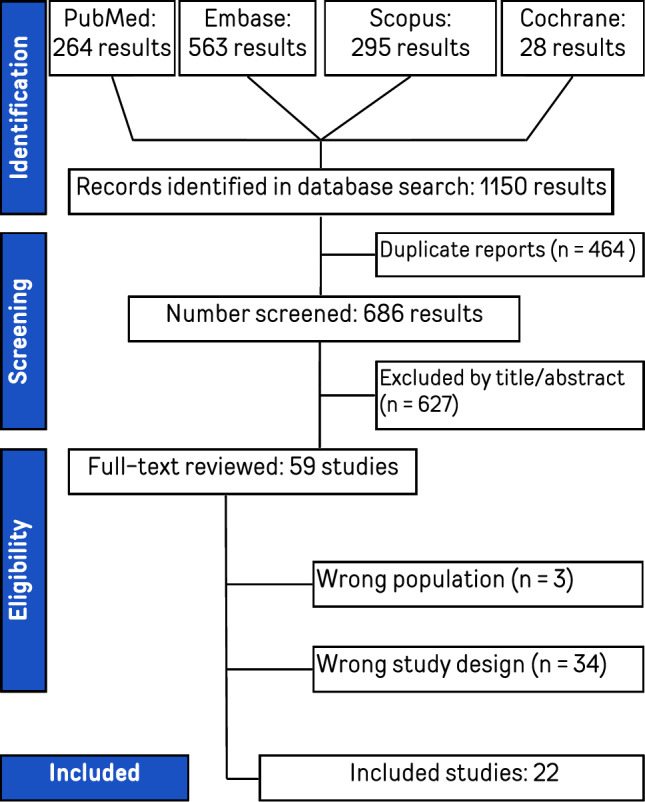
PRISMA flow diagram of study screening and selection

The initial search identified 1155 studies across PubMed, Embase, Scopus, and the Cochrane Library. After removing 464 duplicates and screening 686 titles and abstracts, 59 studies were selected for full-text assessment according to the prespecified criteria. Of these, 34 were excluded due to wrong study design and 3 due to wrong population, resulting in 22 studies included in the final analysis (Fig. 1).

A total of 22 studies were included in the analysis, comprising 3 RCTs and 19 retrospective cohort studies. Thirteen studies compared EUS-GE vs SGJ, enrolling 5,102 (22.97%) patients in the EUS-GE group and 17,109 (77.03%) in the SGJ group, for a total of 22,211 patients. Sex distribution was reported inconsistently across studies, with 551 (2.48%) females and 665 males (2.99%) among those providing these data. The mean age was 67.12 years in the EUS-GE group and 71.78 years in the SGJ group. Eleven studies compared EUS-GE vs enteral stenting, including 706 (47.73%) patients undergoing EUS-GE and 773 (52.27%) receiving enteral stenting, 1,479 patients in total. Among these, 690 (46.65%) females and 789 (53.35%) males were reported, with mean ages of 66.88 years for EUS-GE and 66.68 years for enteral stenting. All baseline characteristics are detailed in Table 1 (EUS-GE vs SGJ) and Table 2 (EUS-GE vs enteral stenting) [30, 47–67].

**Table 1:** Individual characteristics of the included studies for EUS-GE vs surgical gastrojejunostomy

Study	Study design	Follow-up (days)	Country	EUS-GE technique (*n*)	SGJ technique (*n*)	Number of patients (*n*)			Female:male (*n*)			Age (years) (mea*n* ± SD)		Type of malignancy	
						EUS-GE	SGJ	Total	EUS-GE	SGJ	Total	EUS-GE	SGJ	EUS-GE	SGJ
Abbas, 2022 [47]	Retrospective cohort	I 98 C 199	USA	Direct/ freehand: 25	Laparoscopic: 2 Open: 25	25	27	52	14:11	14:13	28:24	65.35 ± 10.21	61.50 ± 14.87	Pancreatic: 40.00% Gastric: 4.00% Cholangiocarcinoma: 20.00% Other: 36.00%	Pancreatic: 33.00% Gastric: 22.00% Cholangiocarcinoma: 4.00% Small bowel: 11.00% Other: 30.00%
Bang, 2025 [48]	RCT	180	USA, India, and Germany	Direct/ freehand: 38	Laparoscopic: 10 Robotic: 5 Open: 21	38	36	74	15:23	15:21	30:44	65.30 ± 15.10	61.50 ± 12.30	Pancreaticobiliary: 57.90% Gastroduodenal: 18.40% Other: 23.70%	Pancreaticobiliary: 55.60% Gastroduodenal: 36.10% Other: 8.30%
Bronswijk, 2021 [49]	Retrospective cohort*	I 76 C 122	Netherlands, Italy, and Belgium	Wireless simplified technique: 37	Laparoscopic: 37	37	37	74	18:19	15:22	33:41	65.50 ± 12.50	66.40 ± 11.10	Pancreatic: 40.50% Biliary/gall bladder: 13.50% Gastric: 13.50% Duodenal: 16.20% Benign disease: 5.40% Breast cancer: 2.70% Colorectal: 5.40% Neuroendocrine tumor: 2.70% Non-small cell lung: 2.70%	Pancreatic: 35.10% Biliary/gall bladder: 5.40% Gastric: 13.50% Duodenal: 21.60% Benign disease: 5.40% Breast cancer: 2.70% Ampullary: 2.70% Non-small cell lung: 2.70%
Canakis, 2023 [50]	Retrospective cohort	196	USA	Direct/ freehand with oroenteric-assisted jejunal distension: 187	Laparoscopic: 46 Open: 77	187	123	310	76:111	68:55	144:166	67.50 ± 12.60	61.60 ± 13.50	Pancreatic: 38.50% Gastric: 9.63% Duodenal: 14.40% Biliary: 3.74% Metastatic: 21.40% Extraluminal: 11.80% Gallbladder: 0.53%	Pancreatic: 53.70% Gastric: 7.32% Duodenal: 7.32% Biliary: 8.13% Metastatic: 5.69% Extraluminal: 14.60% Gallbladder: 3.25%
Chan, 2023 [51]	Retrospective cohort	I 74 C 177.5	China	EPASS: 30	Laparoscopic: 35	30	35	65	8:22	NR	NR	63.04 ± 13.72	67.78 ± 9.98	Gastric: 26.67% Pancreatic: 26.67% Duodenal: 3.33% Gallbladder: 10.00% Other: 33.33%	Gastric: 68.57% Pancreatic: 20.00% Duodenal: 2.85% Other: 8.58%
Jaruvongvanich, 2023 [52]	Retrospective cohort	I 233 C 331	USA and Belgium	Enteral catheter-assisted: 186 Direct: 22 Ballon catheter-assisted: 11 Pediatric gastroscope-assisted: 10 EPASS: 3	Laparoscopic or exploratory laparotomy	232	73	305	97:135	37:36	134:171	64.50 ± 12.30	62.10 ± 14.40	NR	NR
Khashab, 2017 [53]	Retrospective cohort	I 115 C 196	USA and Japan	EPASS: 22 Balloon-assisted: 6 Direct: 2	Open: 63	30	63	93	13:17	31:32	44:49	70.00 ± 13.30	68.00 ± 9.60	Gastric: 17.60% Ampullary: 6.70% Pancreatic: 56.00% Biliary/Gallbladder: 6.70% Extrinsic/Metastatic: 13.00%	Ampullary: 14.00% Duodenal: 1.50% Pancreatic: 84.50%
Kouanda, 2021 [54]	Retrospective cohort	I 98 C 166	USA	Enteral catheter-assisted: 36	Open: 14	36	14	50	16:20	6:8	22:28	70.40 ± 11.80	71.50 ± 15.60	Pancreatic: 72.20% Ampullary: 2.80% Duodenal: 2.80% Cholangiocarcinoma: 5.60% Gallbladder: 2.80% Metastatic: 13.90%	Pancreatic: 21.40% Gastric: 57.10% Duodenal: 7.10% Cholangiocarcinoma: 7.10% Metastatic: 7.10%
Martinet, 2024 [55]	Retrospective cohort	I 468 C 333	France	Direct or wireless simplified technique: 40	Open: 22	40	22	62	21:19	14:8	35:27	74.58 ± 12.30	68.07 ± 11.88	Gastric: 15.00% Bilio-duodenal-pancreatic: 77.50% Others: 7.50%	Bilio-duodenal-pancreatic: 81.82% Others: 18.18%
Pavert, 2025 [56]	RCT	168	Netherlands	Direct: 48	Laparoscopic: 50	48	50	98	20:28	23:27	43:55	69.40 ± 9.20	70.60 ± 10.50	Pancreatic: 58.00% Duodenal: 8.00% Cholangiocarcinoma: 8.00% Metastasis: 6.00% Gallbladder: 6.00% Ampullary: 4.00% Other: 8.00%	Pancreatic: 50.00% Duodenal: 14.00% Cholangiocarcinoma: 12.00% Metastasis: 6.00% Gallbladder: 4.00% Gastric: 4.00% Other: 10.00%
Pawa, 2023 [57]	Retrospective cohort	NR	USA	Direct: 29	Robotic: 15	29	15	44	10:19	7:8	17:27	66.30 ± 12.60	64.10 ± 13.70	Pancreatic: 52.00% Metastatic: 21.00% Duodenal: 10.00% Cholangiocarcinoma: 7.00% Hepatocellular carcinoma: 3.00% Gastric: 7.00%	Pancreatic: 67.00% Metastatic: 7.00% Duodenal: 13.00% Gastric: 13.00%
Perez-Miranda, 2017 [58]	Retrospective cohort	I 60 C 268.8	USA, Spain, and France	Balloon catheter-assisted: 9 Ultra-slim scope: 7 Nasobiliary drain catheter: 3 Direct: 6	Laparoscopic: 29	25	29	54	14:11	7:22	21:33	63.90^§^	75.80^§^	NR	NR
Pinnam, 2025 [30]	Retrospective cohort	NR	USA	NR	NR	4345	16,585	20,930	NR	NR	NR	66.90^§^	67.10^§^	Gastric: 71.99% Duodenal: 8.44% Pancreatic: 19.56%	Gastric: 55.16% Duodenal: 8.67% Pancreatic: 36.16%

*Propensity score-matched study. ^§^Data reported as mean. *EPASS* Endoscopic ultrasound-guided balloon-occluded gastrojejunostomy bypass. *EUS-GE* Endoscopic ultrasonography-guided gastroenterostomy. *SGJ* Surgical gastrojejunostomy. *I* Intervention. *C* Control

**Table 2:** Individual characteristics of the included studies for EUS-GE vs Enteral Stenting

Study	Study design	Follow-up (days)	Country	EUS-GE technique (*n*)	Stent type (*n*)	Number of patients (*n*)			Female:male (*n*)			Age (years) (mean ± SD)		Type of malignancy	
						EUS-GE	ES	Total	EUS-GE	ES	Total	EUS-GE	ES	EUS-GE	ES
Bellocchi, 2024 [59]	Retrospective cohort*	I 80 C 99	Italy	Direct freehand: 45	SEMS (Uncovered Enteral Wallflex; 22 mm)	45	45	90	27:18	24:21	51:39	68.90 ± 11.50	70.00 ± 10.00	Pancreatic: 66.70% Other: 33.30%	Pancreatic: 82.20% Other: 17.80%
Chan, 2023 [51]	Retrospective cohort	I 74 C 54	China	EPASS: 30	SEMS (Uncovered Wallflex or Niti-S)	30	49	79	8:22	19:30	27:52	63.04 ± 13.72	69.31 ± 12.30	Gastric: 26.67% Pancreatic: 26.67% Duodenal: 3.33% Gallbladder: 10.00% Others: 33.33%	Gastric: 36.74% Pancreatic: 36.74% Duodenal: 6.12% Gallbladder: 8.16% Others: 12.24%
Chen, 2017 [60]	Retrospective cohort	I 103 C 23.5	USA and Japan	EPASS: 22 Balloon-assisted: 6 Direct: 2	SEMS (Enteral Wallstent or Duodenal Wallflex; 20 or 22 mm)	30	52	82	13:17	20:32	33:49	70.00 ± 13.30	64.00 ± 13.20	Gastric: 16.70% Duodenal/Ampullary: 6.70% Pancreatic: 56.70% Extrinsic/Metastatic: 13.30% Biliary/Gallbladder: 6.70%	Gastric: 5.80% Duodenal/Ampullary: 13.50% Pancreatic: 53.80% Extrinsic/Metastatic: 19.20% Biliary/Gallbladder: 7.70%
Ge, 2019 [61]	Retrospective cohort	NR	USA	Direct freehand: 22	SEMS (Uncovered duodenal Wallflex or Evolution; 22 mm)	22	78	100	13:9	31:47	44:56	66.40 ± 9.20	65.70 ± 12.60	Gastric: 4.60% Duodenal: 4.60% Pancreatic: 31.80% Biliary (gall bladder or cholangiocarcinoma): 18.20% Metastatic: 40.90%	Gastric: 10.30% Duodenal: 1.30% Pancreatic: 51.30% Ampullary: 2.60% Biliary (gall bladder or cholangiocar- cinoma): 10.30% Metastatic: 24.40%
Jaruvongvanich, 2023 [52]	Retrospective cohort	I 233 C 56	USA and Belgium	Enteral catheter-assisted: 186 Direct: 22 Ballon catheter-assisted: 11 Pediatric gastroscope-assisted: 10 EPASS: 3	SEMS (Uncovered)	232	131	363	97:135	64:67	161:202	64.50 ± 12.30	66.90 ± 11.80	NR	NR
Mahajan, 2025 [62]	Retrospective cohort	NR	India	Direct freehand: 60	SEMS (Uncovered duodenal WallFlex; 22 mm)	60	112	172	28:32	49:63	77:95	54.52 ± 12.75	53.63 ± 12.16	Gastric: 16.66% Periampullary: 15.00% Pancreatic: 35.00% Gallbladder: 25.00% Others: 16.66%†	Gastric: 26.79% Periampullary: 11.60% Pancreatic: 23.22% Gallbladder: 26.79% Others: 11.60%
Sánchez- Aldehuelo, 2023 [63]	Retrospective cohort	NR	Spain	Free-hand EUS-GE with nasobiliary catheter and methylene blue distension: 79	SEMS (Uncovered duodenal Wallflex; 22 mm)	79	97	176	36:43	39:58	75:101	72.40 ± 10.70	70.80 ± 11.70	Pancreatic: 62.00% Gastric: 19.00% Duodenal: 6.30% Biliary/Gallbladder: 6.30% Others: 6.30%	Pancreatic: 46.40% Gastric: 27.80% Duodenal: 6.20% Biliary/Gallbladder: 8.30% Others: 11.30%
Seitz, 2024 [64]	Retrospective cohort*	NR	Germany	Direct: 42 Guidewire-assisted: 2	SEMS (Uncovered duodenal Wallflex; 22 mm)	44	44	88	24:20	23:21	47:41	75.00^§^	74.00^§^	Pancreatic: 51.00% Gastric: 16.30% Biliary: 6.10% Other: 26.50%	Pancreatic: 48.20% Gastric: 10.70% Biliary: 8.90% Other: 32.10%
von Wanrooij, 2022 [65]	Retrospective cohort*	I 103 C 51	Netherlands, Belgium and Italy	EPASS: 88	SEMS (Uncovered duodenal Wallflex; 22 mm)	88	88	176	44:44	48:40	92:84	66.00 ± 12.10	66.00 ± 10.40	Gastric: 9.10% Duodenal: 9.10% Pancreatic: 56.80% Biliary: 12.50% Other: 12.50%	Gastric: 7.90% Duodenal: 11.40% Pancreatic: 63.60% Biliary: 5.70% Other: 11.40%
Teoh, 2025 [66]	RCT	NR	China, Belgium, Brazil, India, Italy, and Spain	Wireless simplified technique: 48	SEMS (Uncovered duodenal Wallflex; 22 mm)	48	49	97	23:25	28:21	51:46	69.50 ± 12.60	64.80 ± 13.00	Pancreatic: 44.00% Gastric: 29.00% Duodenal: 10.00% Periampullary: 8.00% Cholangiocarcinoma: 4.00% Gallbladder: 4.00%	Pancreatic: 47.00% Gastric: 29.00% Duodenal: 12.00% Periampullary: 8.00% Cholangiocarcinoma: 2.00% Gallbladder: 2.00%
Vanella, 2023 [67]	Prospective cohort*	I 73 C 78	Italy	Wireless simplified technique: 28	SEMS (Uncovered duodenal Wallflex or Evolution; 22 mm)	28	28	56	14:14	18:10	32:24	65.42 ± 10.93	68.42 ± 12.49	Pancreatic: 64.30% Ampullary: 7.10% Biliary/Gallbladder: 10.70% Gastric: 7.10% Others: 10.70%	Pancreatic: 71.40% Ampullary: 10.70% Biliary/Gallbladder: 7.10% Gastric: 7.10% Others: 3.60%

### EUS-GE vs surgical gastrojejunostomy

#### Technical and clinical success

**Fig. 2:**
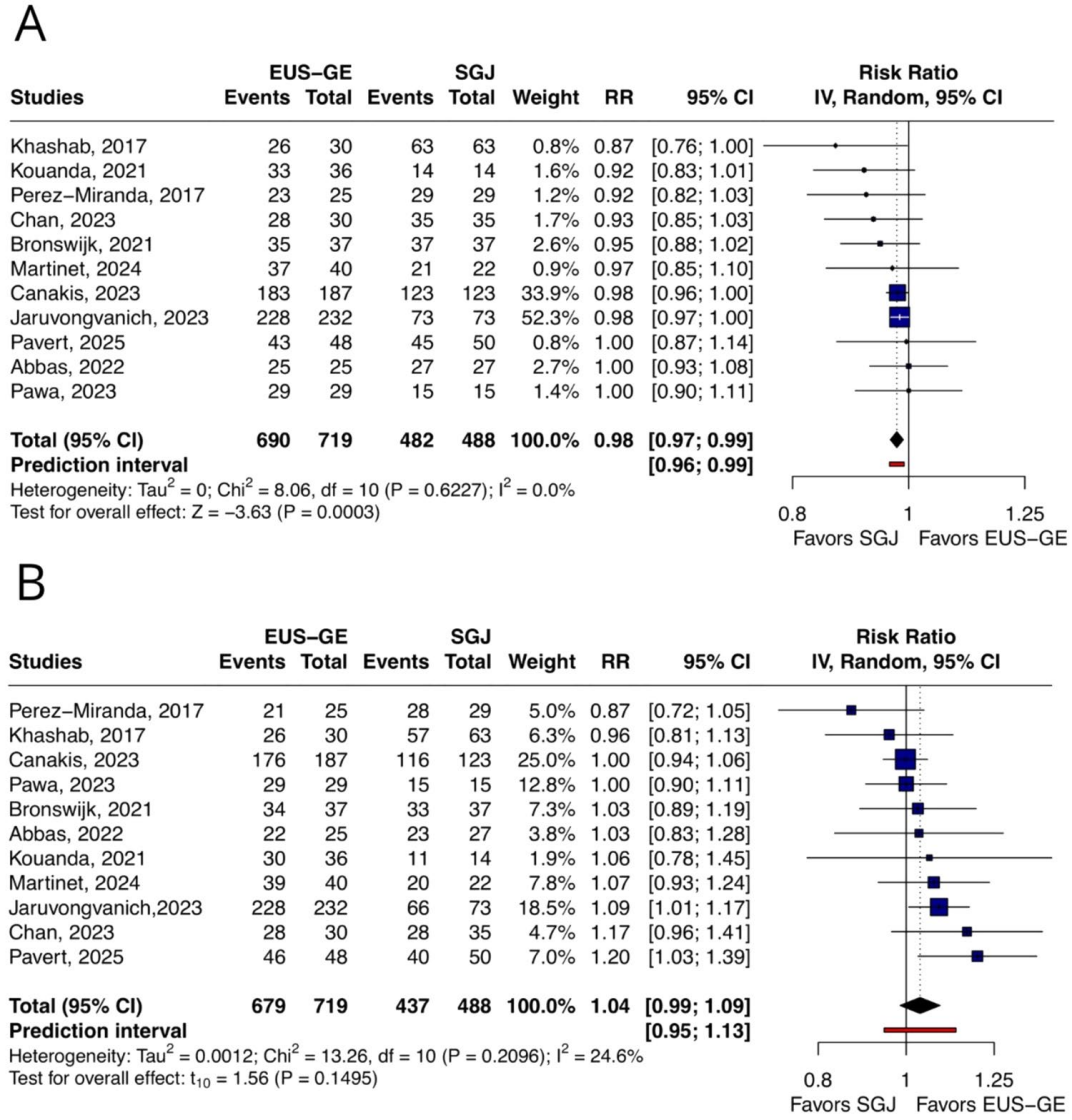
Forest plots comparing EUS-GE and SGJ in MGOO patients for **A** technical success and **B** clinical success outcomes

The pooled analysis demonstrated that SGJ was associated with higher technical success than EUS-GE (98.77% vs 95.96%; RR 0.98; 95% CI 0.97 to 0.99; *p* = 0.0003; I^2^ = 0%; Fig. 2A). The prediction interval indicated a consistent direction of effect across potential future studies (PI 0.96 to 0.99).

For clinical success, no statistically significant difference was observed between EUS-GE and SGJ (94.43% vs 89.54%; RR 1.04; 95% CI 0.99 to 1.09; *p* = 0.1495; *I*^2^ = 24.60%; Fig. 2B).

#### Postoperative complications and Clavien–Dindo grade

**Fig. 3:**
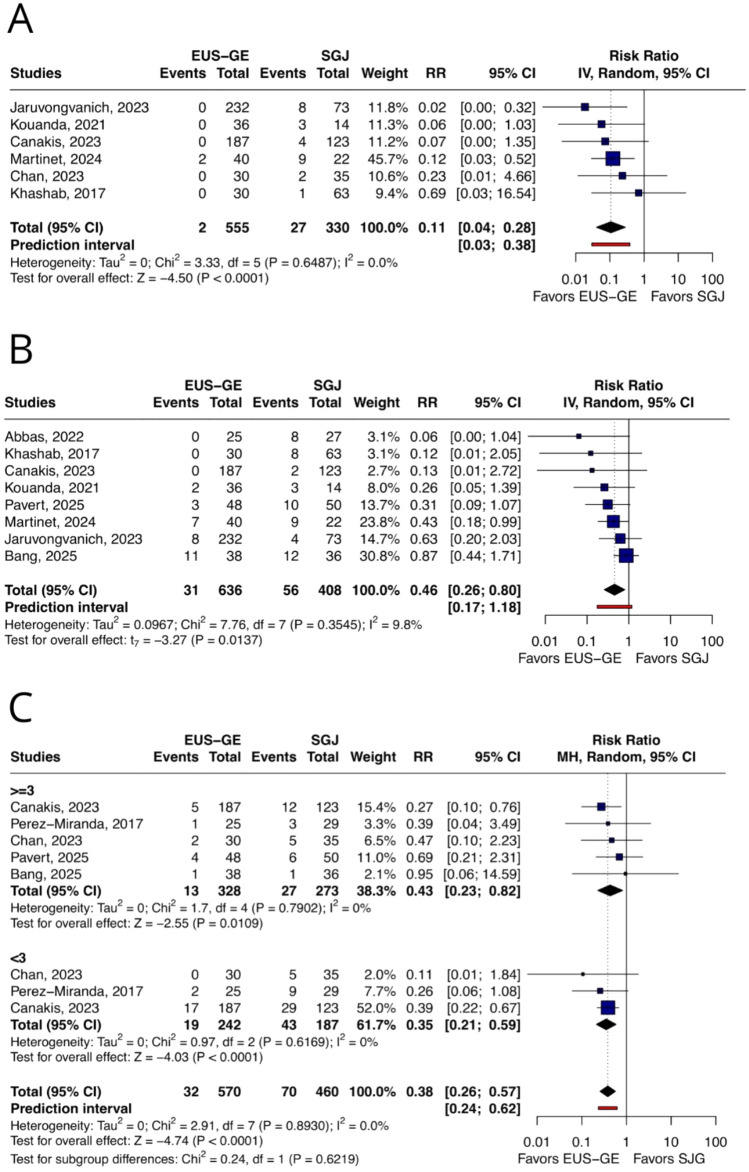
Forest plots comparing EUS-GE and SGJ in MGOO patients for **A** postoperative ileus, **B** postoperative infections, and **C** Clavien–Dindo complication grades stratified into ≥ 3 versus < 3

Regarding postoperative ileus, EUS-GE was associated with a reduction in this complication (0.36% vs 8.18%; RR 0.11; 95% CI 0.04 to 0.28; *p* < 0.0001; *I*^2^ = 0%; Fig. 3A). The prediction interval (PI 0.03 to 0.38) demonstrated a consistent direction of effect.

Infections also occurred less frequently among patients undergoing EUS-GE compared with SGJ (4.87% vs 13.72%; RR 0.46; 95% CI 0.26 to 0.80; *p* = 0.0137; *I*^2^ = 9.80%; Fig. 3B). The prediction interval (PI 0.17 to 1.18) suggests that future studies may show variable magnitudes of benefit.

Similarly, sepsis was less frequent in patients undergoing EUS-GE (2.71% vs 4.58%; RR 0.62; 95% CI 0.51 to 0.74; *p* < 0.0001; *I*^2^ = 0%; Supplementary Fig. 1). The prediction interval (PI 0.46 to 0.84) confirmed this finding.

Anastomotic leak was also less common with EUS-GE (0.30% vs 5.76%; RR 0.17; 95% CI 0.04 to 0.68; *p* = 0.0124; *I*^2^ = 0%; Supplementary Fig. 2). The prediction interval (PI 0.02 to 1.61) indicated wide uncertainty due to limited events.

In contrast, for perforation, no statistically significant difference was found between EUS-GE and SGJ (1.12% vs 1.08%; RR 1.05; 95% CI 0.67 to 1.65; *p* = 0.7551; *I*^2^ = 0%; Supplementary Fig. 3).

Overall postoperative complications graded by Clavien–Dindo were significantly reduced with EUS-GE compared with SGJ (5.61% vs 15.21%; RR 0.38; 95% CI 0.26 to 0.57; *p* < 0.0001; *I*^2^ = 0%; Fig. 3C). The prediction interval (PI 0.24 to 0.62) confirmed the consistency of this association. Both major (≥ 3) (3.96% vs 9.89%; RR 0.43; 95% CI 0.23 to 0.82; *p* = 0.0109; *I*^2^ = 0) and minor complications (< 3) (7.85% vs 22.99%; RR 0.35; 95% CI 0.21 to 0.59; *p* < 0.0001; *I*^2^ = 0%) were less frequent with EUS-GE. No significant subgroup differences were detected (*χ*^2^ = 0.24, *p* = 0.6219), indicating consistent effects across severity strata.

#### Postoperative mortality

No statistically significant difference in overall postoperative mortality was observed between EUS-GE and SGJ (4.80% vs 3.66%; RR 0.87; 95% CI 0.65 to 1.15; *p* = 0.2645; *I*^2^ = 43.40%; Supplementary Fig. 4). Similarly, no significant differences were identified for 30-day mortality, inpatient mortality, or follow-up mortality, and subgroup analyses showed consistent findings across time-based strata.

#### Reintervention and recurrent obstruction

For reintervention, no statistically significant difference was found between EUS-GE and SGJ (8.68% vs 10.73%; RR 0.67; 95% CI 0.13 to 3.56; *p* = 0.5816; *I*^2^ = 78.10%; Supplementary Fig. 5).

Similarly, for recurrent obstruction, no statistically significant difference was observed (9.47% vs 21.42%; RR 0.58; 95% CI 0.29 to 1.18; *p* = 0.1341; *I*^2^ = 0%; Supplementary Fig. 6).

#### Length of hospital stay and operative time

**Fig. 4:**
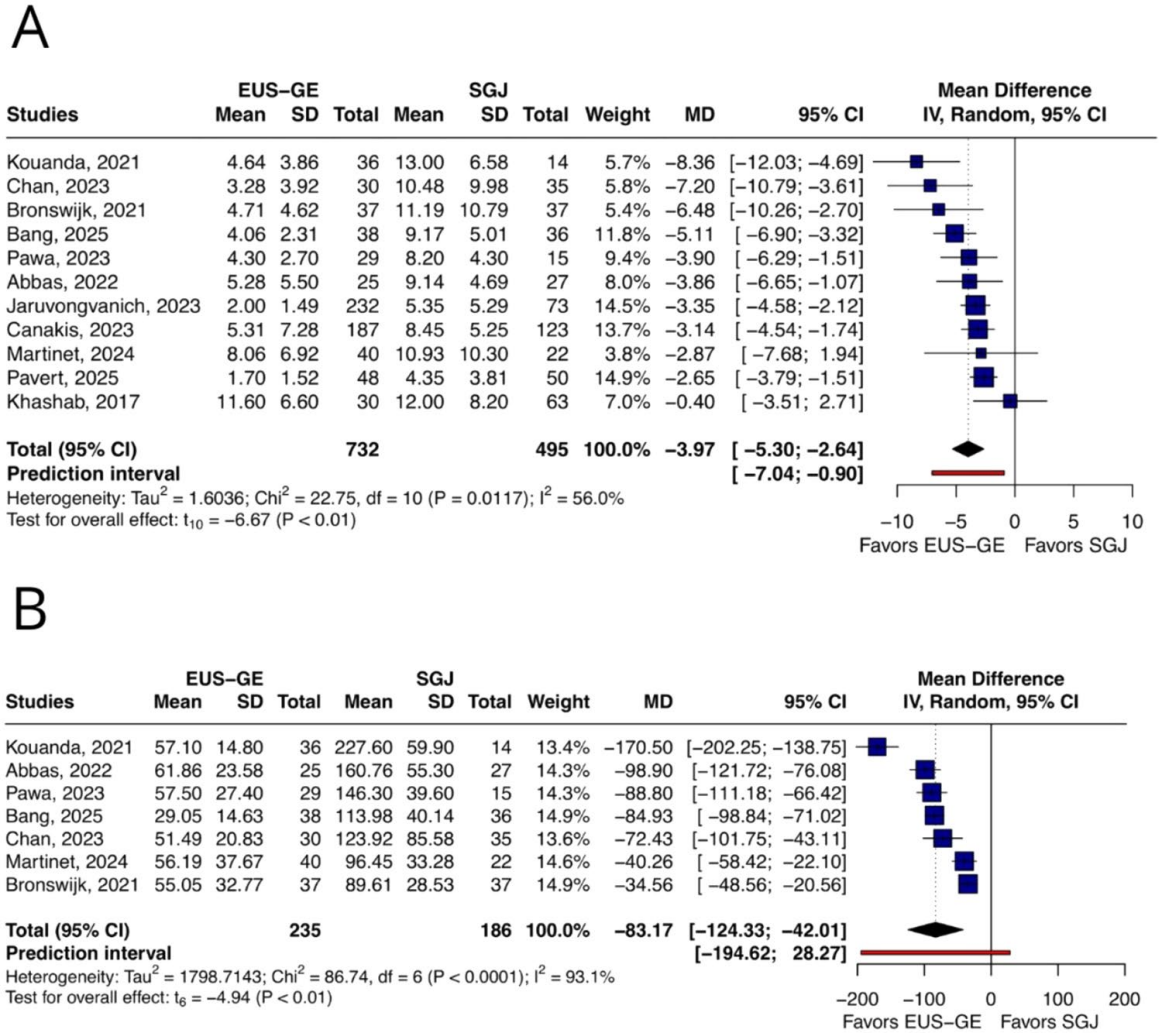
Forest plots comparing EUS-GE and SGJ in MGOO patients for **A** length of hospital stay and **B** operative time

EUS-GE was associated with a reduction in hospital length of stay compared with SGJ (MD –3.97 days; 95% CI –5.30 to –2.64; *p* < 0.01; *I*^2^ = 56.00%; Fig. 4A). The prediction interval (PI –7.04 to –0.90) indicated that future studies are also likely to favor EUS-GE. However, in the subgroup analysis restricted to RCTs and studies using propensity score matching, the reduction in hospital stay was no longer statistically significant (MD –4.32 days; 95% CI –8.99 to 0.36; *p* = 0.06; *I*^2^ = 73.70%; Supplementary Fig. 7).

Similarly, operative time was shorter in the EUS-GE group (MD –83.17 min; 95% CI –124.33 to –42.01; *p* < 0.01; *I*^2^ = 93.10%; Fig. 4B). The prediction interval (PI –194.62 to 28.27) indicated substantial between-study variability, suggesting that future studies may not favor EUS-GE.

#### Time to oral intake

EUS-GE was associated with a reduction in time to oral intake compared with SGJ (MD –2.46 days; 95% CI –2.89 to –2.03; *p* < 0.01; *I*^2^ = 1.90%; Supplementary Fig. 8). The prediction interval (PI –3.15 to –1.76) indicated that future studies are also likely to favor EUS-GE in terms of earlier resumption of oral intake.

### EUS-GE vs enteral stenting

#### Technical and clinical success

**Fig. 5:**
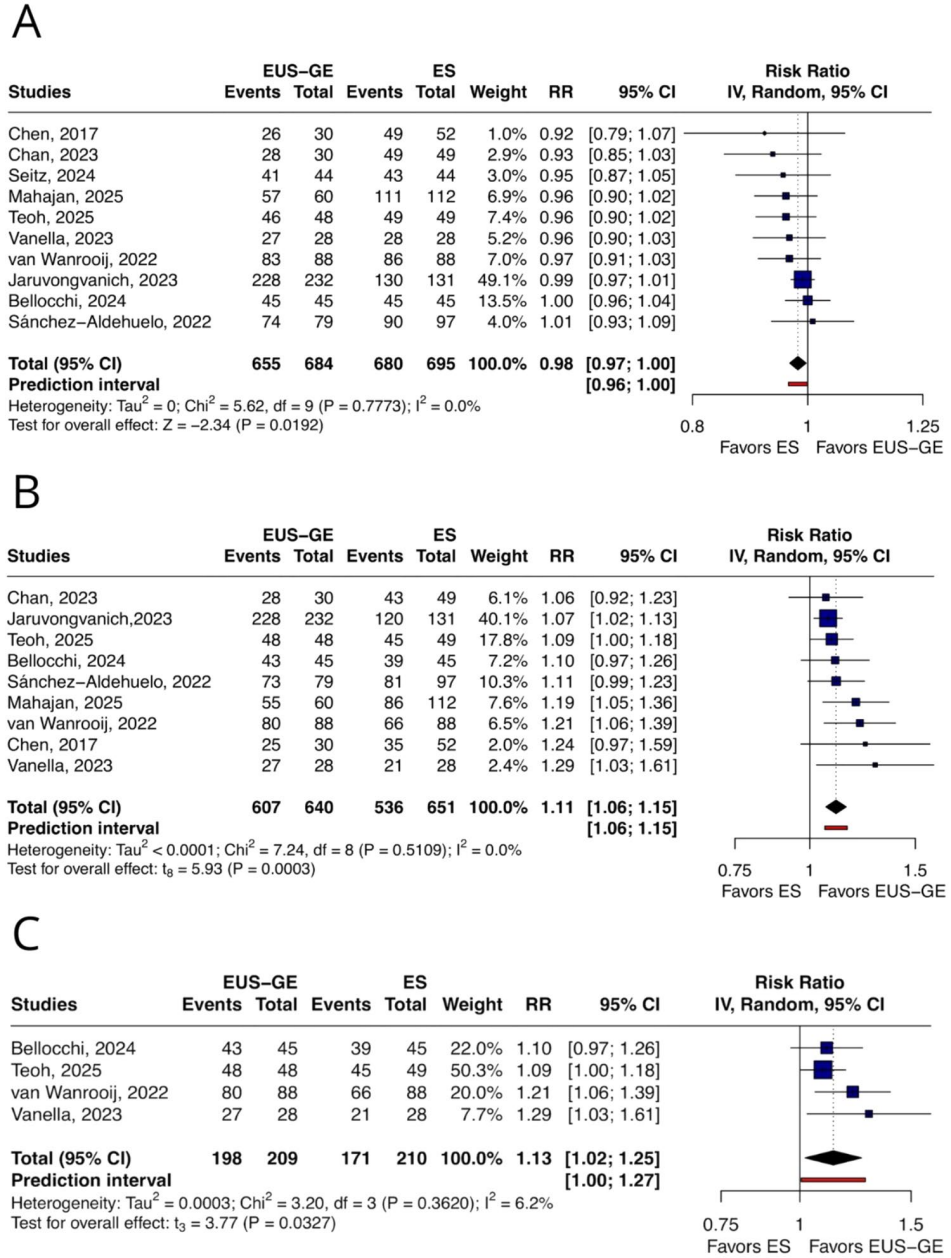
Forest plots comparing EUS-GE and enteral stenting (ES) in MGOO patients for **A** technical success, **B** clinical success, and **C** subgroup analyses of clinical success (randomized controlled trials and propensity score-matched cohort studies)

Regarding technical success, EUS-GE and enteral stenting demonstrated comparable outcomes, with no statistically significant difference between groups (95.76% vs 97.84%; RR 0.98; 95% CI 0.97 to 1.00; *p* = 0.0192; *I*^2^ = 0%; Fig. 5A). In the subgroup analysis restricted to RCTs and PSM studies, results were consistent with the main analysis (95.65% vs 98.81%; RR 0.98; 95% CI 0.95 to 1.00; *p* = 0.0687; *I*^2^ = 0%; Supplementary Fig. 9).

For clinical success, EUS-GE was associated with a higher clinical success rate compared with enteral stenting (94.84% vs 82.33%; RR 1.11; 95% CI 1.06 to 1.15; *p* = 0.0003; *I*^2^ = 0%; Fig. 5B). The prediction interval (PI 1.06 to 1.15) indicated that this advantage for enteral stenting is expected to persist across future studies. In the subgroup analysis restricted to RCTs and PSM studies, the findings remained consistent with the main analysis (94.73% vs 81.42%; RR 1.13; 95% CI 1.02 to 1.25; *p* = 0.0327; *I*^2^ = 6.20%; Fig. 5C). The prediction interval (1.00 to 1.27) suggested that while some variability is expected, the overall trend continues to favor EUS-GE.

#### Postoperative complications

**Fig. 6:**
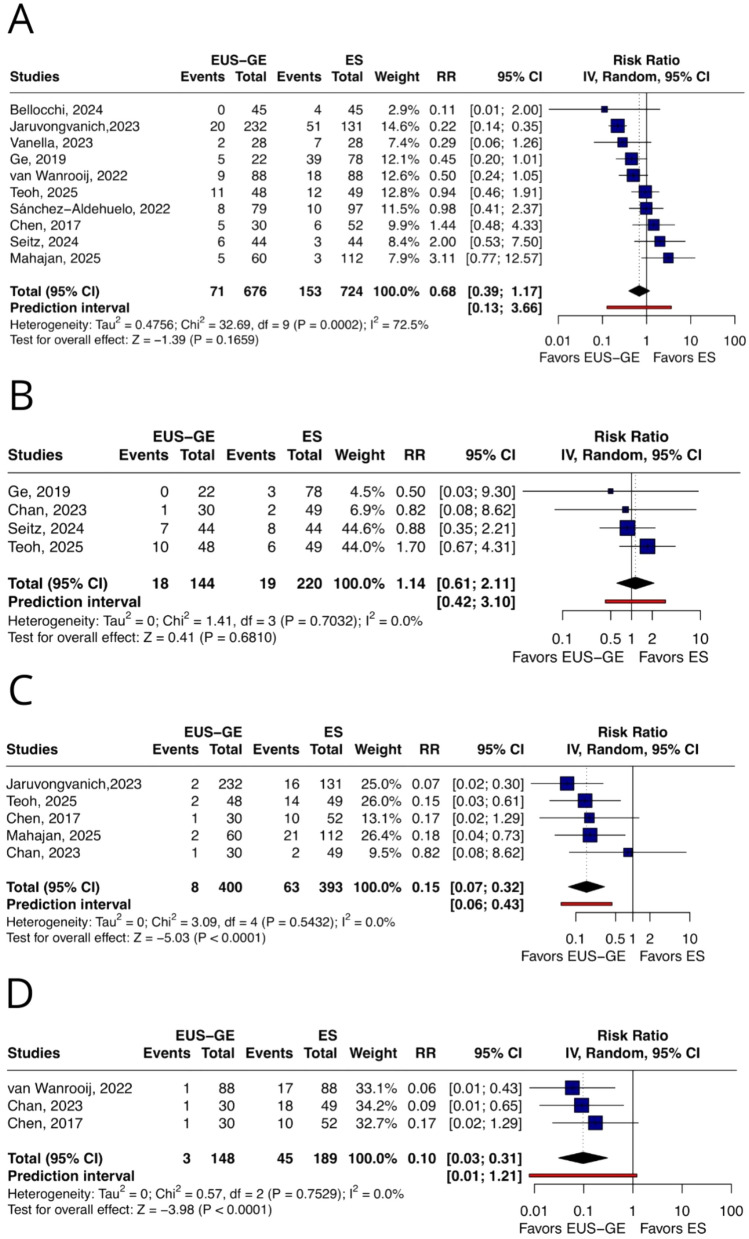
Forest plots comparing EUS-GE and enteral stenting (ES) in MGOO patients for **A** postoperative complications, **B** 30-day mortality, **C** reintervention, and **D** recurrent obstruction

For postoperative complications, no statistically significant difference was found between EUS-GE and enteral stenting (10.50% vs 21.13%; RR 0.68; 95% CI 0.39 to 1.17; *p* = 0.1659; *I*^2^ = 72.50%; Fig. 6A). In the subgroup including RCTs and PSM studies, results were consistent, with no statistically significant difference between EUS-GE and enteral stenting (11.06% vs 17.32%; RR 0.67; 95% CI 0.26 to 1.77; *p* = 0.3188; *I*^2^ = 41.00%; Supplementary Fig. 10).

#### 30-day mortality

For 30-day postoperative mortality, no statistically significant difference was observed between EUS-GE and enteral stenting (12.50% vs 8.63%; RR 1.14; 95% CI 0.61 to 2.11; *p* = 0.6810; *I*^2^ = 0%; Fig. 6B).

#### Reintervention and recurrent obstruction

For reintervention, the pooled analysis demonstrated that EUS-GE was associated with a markedly lower need for subsequent procedures compared with enteral stenting (2.00% vs 16.03%; RR 0.15; 95% CI 0.07 to 0.32; *p* < 0.0001; *I*^2^ = 0%; Fig. 6C). The prediction interval (PI 0.06 to 0.43) indicates that this benefit is expected to persist in future clinical settings, consistently favoring EUS-GE.

For recurrent obstruction, EUS-GE was associated with a lower risk of recurrence compared with enteral stenting (2.02% vs 23.80%; RR 0.10; 95% CI 0.03 to 0.31; *p* < 0.0001; *I*^2^ = 0%; Fig. 6D). However, the wider prediction interval (PI 0.01 to 1.21) indicates that future studies may not consistently favor EUS-GE.

#### Length of hospital stay

For length of hospital stay, no statistically significant difference was observed between EUS-GE and enteral stenting (MD –1.99 days; 95% CI –4.12 to 0.15; *p* = 0.06; *I*^2^ = 68.50%; Supplementary Fig. 11). In the subgroup combining RCTs and PSM studies, results remained consistent, with no significant difference between groups (MD –2.18 days; 95% CI –6.36 to 2.01; *p* = 0.20; *I*^2^ = 70.30%; Supplementary Fig. 12).

### Quality assessment

The RCTs assessed with RoB 2 (Supplementary Fig. 13) demonstrated uniformly low risk of bias across all domains, with no concerns related to randomization, deviations from intended interventions, missing data, outcome measurement, or selective reporting, indicating high overall methodological reliability. In contrast, the observational studies evaluated with ROBINS-I (Supplementary Fig. 14) showed predominantly low-to-moderate risk across most domains, although several studies presented serious concerns, mainly regarding confounding (D1), resulting in moderate-to-serious overall risk of bias in a subset of non-randomized evidence.

### Sensitivity analysis

Leave-one-out sensitivity analyses were conducted for length of hospital stay, mortality, operative time, and reintervention in the EUS-GE versus SGJ comparison. For mortality, exclusion of Pinnam et al. [30] reduced heterogeneity to *I*^2^ = 4.30% without changing the pooled effect estimate. No meaningful changes in heterogeneity or effect estimates were observed for length of hospital stay, operative time, or reintervention (Supplementary Figs. 15–18).

Leave-one-out sensitivity analyses were performed for postoperative complications and length of hospital stay in the EUS-GE versus enteral stenting comparison. Omission of individual studies did not meaningfully alter heterogeneity or pooled effect estimates for either outcome (Supplementary Figs. 19–20).

In the comparison between EUS-GE and SGJ, the Baujat analysis identified Bang et al. for mortality and Jaruvongvanich et al. for reintervention as the main contributors to heterogeneity, which was not consistent with the leave-one-out analysis. In contrast, Kouanda et al. was consistently identified as influential for length of hospital stay and operative time in both analyses (Supplementary Figs. 21–24).

In the comparison between EUS-GE and enteral stenting, the Baujat and leave-one-out analyses were consistent, identifying Jaruvongvanich et al. for postoperative complications and van Wanrooij et al. for length of hospital stay as the main contributors to heterogeneity (Supplementary Figs. 25–26).

### Publication bias

In the comparison between EUS-GE and SGJ, funnel plots for both technical success and clinical success were visually symmetric, indicating no apparent publication bias. This visual assessment was supported by Egger’s regression test, which did not demonstrate significant funnel plot asymmetry (*p* = 0.0568 and *p* = 0.6586, respectively), suggesting the absence of small-study effects.

In contrast, in the comparison between EUS-GE and enteral stenting, funnel plots for both technical success and clinical success were asymmetric. Accordingly, Egger’s regression test indicated significant funnel plot asymmetry for these outcomes (*p* = 0.0251 and *p* = 0.0134, respectively), suggesting the presence of potential small-study effects or selective reporting. Funnel plots are presented in Supplementary Figs. 27–30.

### Trial sequential analysis

**Fig. 7:**
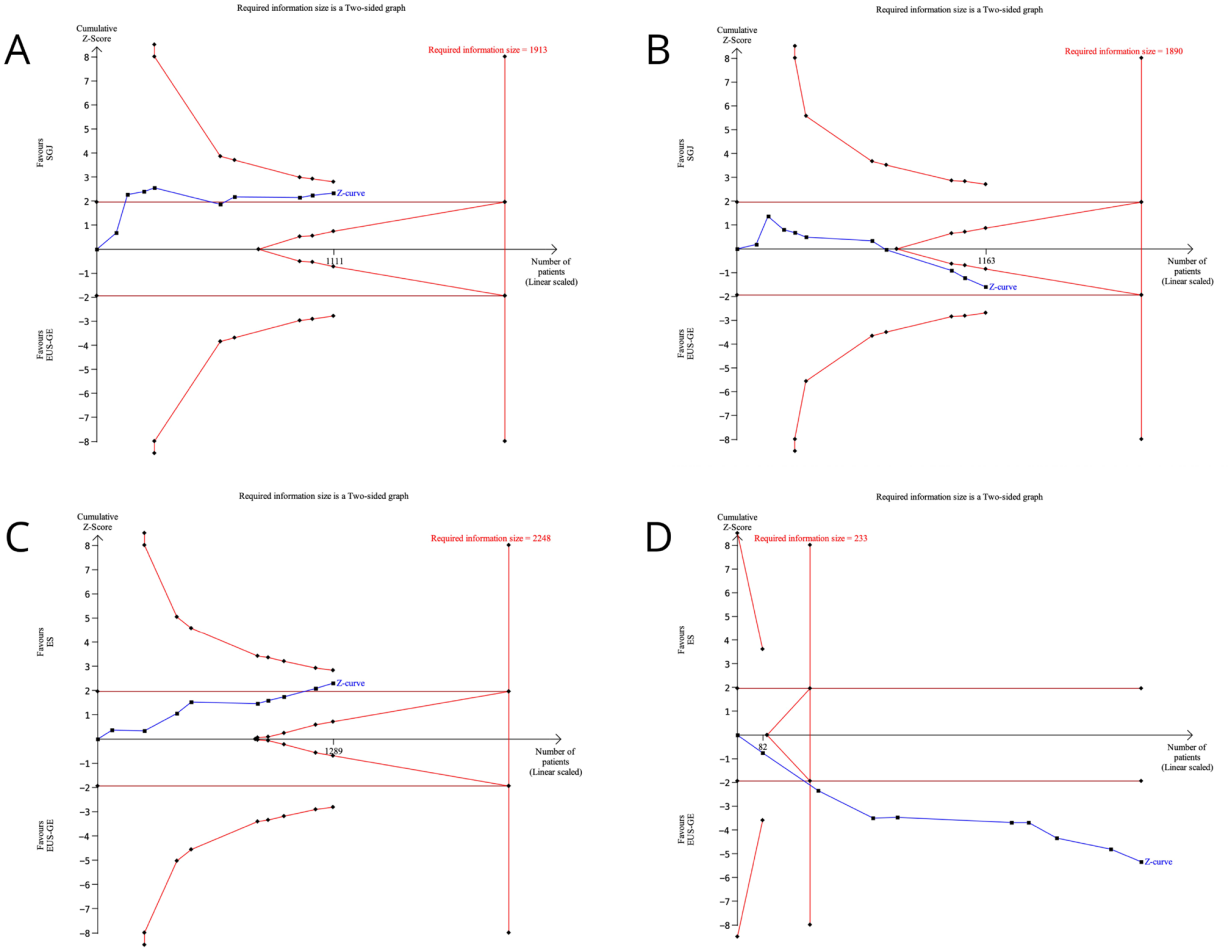
Trial sequential analysis comparing EUS-GE with SGJ for **A** technical success and **B** clinical success and with enteral stenting for **C** technical success and **D** clinical success

For the comparison between EUS-GE and SGJ, TSA was performed for technical success, clinical success, and overall postoperative complications (all-grade Clavien–Dindo). For technical success, the cumulative Z curve crossed the conventional boundary for statistical significance favoring SGJ over EUS-GE but did not cross the trial sequential monitoring boundary for benefit nor reach the required information size (1913 patients); at the final cumulative sample size (1,111 patients), the evidence remained insufficient and susceptible to random error (Fig. 7A). For clinical success, the Z-curve crossed neither the conventional nor the trial sequential monitoring boundaries, and the accrued sample size (1,163 patients) did not reach the required information size (1,890 patients); the Z curve remained within the inner boundaries without entering the futility area, indicating inconclusive evidence rather than absence of effect (Fig. 7B). Additionally, for overall postoperative complications (all-grade Clavien–Dindo), the cumulative Z curve crossed the conventional boundary and exceeded the required information size (182 patients); however, it did not cross the trial sequential monitoring boundary. These findings suggest that, although statistically significant by conventional meta-analysis, the evidence remains potentially subject to random error and is not yet conclusive (Supplementary Fig. 31).

For the comparison between EUS-GE and enteral stenting, TSA was also performed for technical success, clinical success, and reintervention. For technical success, the cumulative Z curve crossed the conventional boundary for statistical significance favoring enteral stenting but did not cross the trial sequential monitoring boundary for benefit nor reach the required information size (2,248 patients), with an accrued sample size of 1,289 patients, indicating insufficient evidence and risk of random error (Fig. 7C). For clinical success, the cumulative Z curve crossed the conventional boundary for benefit favoring EUS-GE and exceeded the required information size (233 patients); however, it did not cross the trial sequential monitoring boundary, indicating that the evidence remains inconclusive (Fig. 7D). Furthermore, for reintervention, the cumulative Z-curve crossed both the conventional and trial sequential monitoring boundaries for benefit favoring EUS-GE and surpassed the required information size (131 patients), indicating firm evidence with a low risk of random error (Supplementary Fig. 32).

## Discussion

In this systematic review and meta-analysis of 22 studies, we included 5,102 patients undergoing EUS-GE and 17,109 undergoing SGJ, as well as a separate comparison of 706 EUS-GE and 773 enteral stenting patients, to evaluate treatment strategies for MGOO. When compared with SGJ, EUS-GE was associated with a higher risk of technical failure, but significantly lower rates of postoperative complications, including ileus, infections, sepsis, anastomotic leak, and both major and minor complications. Additionally, EUS-GE was associated with shorter operative time, shorter hospital stay, and earlier return to oral intake. No significant differences were observed between EUS-GE and SGJ in terms of clinical success, postoperative mortality, reintervention, or recurrent obstruction. When compared with enteral stenting, EUS-GE demonstrated higher clinical success and significantly lower rates of reintervention and recurrent obstruction. No significant differences were observed in technical success, postoperative complications, 30-day mortality, or length of hospital stay.

Current guideline recommendations for the management of MGOO emphasize a tailored approach based on patient characteristics, life expectancy, and local expertise. The American Society for Gastrointestinal Endoscopy (ASGE) suggests either duodenal SEMS placement or SGJ as palliative options for patients with incurable MGOO, with the choice driven by clinical condition, expected survival, and shared decision-making. Specifically, SEMS placement is favored in patients with poor surgical candidacy or short life expectancy (< 6 months), whereas SGJ is recommended for those with > 6 months expected survival and good performance status [33]. In contrast, the European Society of Gastrointestinal Endoscopy (ESGE) guideline provides a strong recommendation, though supported by low-quality evidence, that EUS-GE should be offered in expert centers as an alternative to SEMS or SGJ for malignant obstruction [68]. Recent international reviews further support this shift, highlighting EUS-GE’s potential to achieve durable luminal patency with lower reintervention rates, positioning it as an emerging preferred modality when technical expertise is available [69].

Our findings are consistent with and help contextualize these recommendations. Consistent with ESGE guidance, EUS-GE demonstrated clear advantages over enteral stenting in our analysis, particularly regarding durability of obstruction relief, with markedly lower rates of reintervention and recurrent obstruction. These results reinforce the role of EUS-GE as a more durable alternative to SEMS in appropriately selected patients. Conversely, when comparing EUS-GE with SGJ, our data revealed comparable clinical success and postoperative mortality, supporting the ASGE position that both procedures represent valid therapeutic options. Importantly, EUS-GE was associated with fewer postoperative complications, such as infections, ileus, and Clavien–Dindo ≥ 3 events, suggesting a more favorable perioperative safety profile. Taken together, these findings support the evolving role of EUS-GE within current treatment algorithms.

The observed differences across treatment strategies can be explained by their distinct physiological and technical characteristics. EUS-GE creates a gastroenteric anastomosis using a LAMS positioned under direct EUS visualization, establishing a stable bypass that is not dependent on tumor ingrowth or extrinsic compression, thereby providing a longer-lasting luminal patency and explaining the markedly lower rates of recurrent obstruction and reintervention observed in our analysis when compared with enteral stenting [70]. In contrast, enteral stents traverse the site of malignant obstruction and therefore remain susceptible to tumor ingrowth, overgrowth, biofilm formation, and stent migration, all of which contribute to early dysfunction and the need for repeated interventions [71]. Additionally, restoration of luminal continuity through a bypass may facilitate earlier resumption of oral intake and improved recovery, aligning with our findings of shorter time to oral intake [72].

On the other hand, EUS-GE requires adequate gastric distension, stable anatomic apposition, and unobstructed access to the target jejunal loop. Accordingly, ESGE guidance notes that massive ascites, peritoneal carcinomatosis, or distorted anatomy from bulky tumor burden are relative contraindications, which may explain the higher likelihood of technical failure in these settings [68]. Moreover, because EUS-GE relies on transmural access and LAMS deployment, risks such as gastric leakage, peritonitis, bowel perforation, and stent misdeployment may account for procedure-specific complications not typically seen with surgical bypass [73]. In contrast, SGJ provides excellent long-term patency but at the cost of greater physiological stress and higher postoperative morbidity, mechanisms consistent with the higher complication profile we observed in comparison to EUS-GE [5]. These mechanistic differences help explain why EUS-GE combines favorable recovery outcomes with durable efficacy.

In the subgroup analyses restricted to RCTs and PSM studies, aimed at reducing baseline imbalances and confounding, the overall interpretation remained consistent with the primary analyses. For the comparison between EUS-GE and enteral stenting, the outcomes that were non-significant in the main analysis (technical success, postoperative complications, and length of hospital stay) also remained non-significant after restricting to higher-quality designs, indicating that these results were not meaningfully influenced by study design. Clinical success continued to favor enteral stenting in both analyses, although the effect was less pronounced in the restricted subgroup, likely reflecting the smaller dataset available for this comparison. In the comparison with surgery, only length of hospital stay could be reassessed, and this was the single outcome in which the significance changed. The difference favoring EUS-GE in the primary analysis was no longer statistically significant in the subgroup analysis. This change likely reflects residual confounding in the observational studies included in the main analysis. Once the comparison was limited to designs with better control of baseline differences, the effect estimate remained in the same direction, but the reduced sample size widened the confidence interval, resulting in a non-significant finding.

Significant heterogeneity was observed across several outcomes, including operative time, length of hospital stay, postoperative complications, and reintervention rates. This variability is likely multifactorial and may be largely explained by differences in procedural techniques, operator experience, and patient selection across studies. Notably, EUS-GE lacks a standardized procedural approach, with multiple techniques described, including direct, balloon-assisted, and endoscopic ultrasound-guided balloon-occluded gastrojejunostomy bypass (EPASS) methods [69]. A multicenter study comparing direct and balloon-assisted approaches demonstrated similar efficacy but significantly shorter mean procedure time with the direct technique (35 vs. 90 min; *p* < 0.001), highlighting substantial variability in procedural complexity [74]. Furthermore, the learning curve associated with EUS-GE is considerable, with proficiency achieved after approximately 25 cases and mastery after 40 cases, which may further contribute to heterogeneity in operative time and complication rates [75]. Adverse event rates have been reported to range from 0 to 30%, which likely explains the variability observed in postoperative complications [70, 73]. In the surgical arm, differences in operative approaches (open versus minimally invasive techniques) may influence recovery time, complication rates, and hospital stay, further contributing to heterogeneity. Additionally, variability in patient characteristics, including disease severity, presence of ascites, and performance status, may impact outcomes such as reintervention and hospitalization.

Our study has important limitations. First, most included studies were observational, which introduces inherent risks of confounding and selection bias. To mitigate this limitation, we performed subgroup analyses restricted to PSM cohorts and RCTs for outcomes with at least three available studies, which helped reduce the impact of baseline imbalances. Second, some outcomes demonstrated substantial statistical heterogeneity. However, leave-one-out sensitivity analyses confirmed the stability of the results, with no relevant changes in the overall effect estimates. Third, it was not possible to evaluate individual EUS-GE techniques separately due to insufficient reporting across studies, limiting technique-specific inferences. Forth, evidence of potential publication bias was identified in the comparison between EUS-GE and enteral stenting, as suggested by funnel plot asymmetry and significant Egger’s regression test results for both technical and clinical success. This finding indicates the possibility of small-study effects or selective reporting, which may lead to overestimation of treatment effects and should be considered when interpreting these outcomes. Finally, as expected in observational research, most non-randomized studies were rated as having a high risk of bias due to confounding, although the complementary subgroup analyses using RCTs and propensity-matched data help to partially address this concern.

Finally, these findings have implications for future research and clinical practice. By incorporating TSA, this study provides a more comprehensive assessment of the strength and reliability of the available evidence. Further efforts toward standardization of the EUS-GE technique, including LAMS type, deployment strategy, adjunctive maneuvers, and procedural reporting, may help reduce heterogeneity across studies and enhance comparability [55]. Given the predominance of observational data, future research may benefit from incorporating sensitivity analyses, such as *E* value calculations, to further assess the robustness of findings against potential unmeasured confounding. Moreover, the development of prediction models or clinical nomograms may improve patient selection and support individualized decision-making in clinical practice. Future research may therefore focus on long-term durability, patient-centered outcomes, and cost-effectiveness rather than short-term efficacy endpoints [69].

## Conclusion

In this systematic review and meta-analysis, EUS-GE was associated with significantly lower rates of postoperative complications and shorter operative time, hospital stay, and time to oral intake compared with SGJ, despite a higher risk of technical failure. When compared with enteral stenting, EUS-GE demonstrated higher clinical success and significantly lower rates of reintervention and recurrent obstruction. In light of these findings, EUS-GE appears to represent a favorable therapeutic option for MGOO in centers with sufficient expertise, combining effectiveness, durability, and safety.

## Supplementary Information

Below is the link to the electronic supplementary material.

Supplementary file 1 (DOCX 10958 KB)
