# Supplementary material for: Endoscopic ultrasonography-guided gastroenterostomy versus conventional approaches for cancer-related malignant gastrointestinal outlet obstruction: a meta-analysis with trial sequential analysis
Source: Surg Endosc. 2026 May 12;40(6):4573–97. doi: 10.1007/s00464-026-12864-9 (PMC13246873; doi:10.1007/s00464-026-12864-9)

**Supplementary Material**

**Supplementary Table 1.** PRISMA 2020 Checklist

**Supplementary Table 2.** PRISMA 2020 for Abstract Checklist

**Supplementary Table 3.** Search Strategies

**Supplementary Figure 1.** Forest plot comparing EUS-GE and SGJ in mGOO patients for postoperative sepsis

**Supplementary Figure 2.** Forest plot comparing EUS-GE and SGJ in mGOO patients for anastomotic leak

**Supplementary Figure 3.** Forest plot comparing EUS-GE and SGJ in mGOO patients for perforation

**Supplementary Figure 4.** Forest plots comparing EUS-GE and SGJ in mGOO patients for mortality across different time intervals (30-day, follow-up, and inpatient)

**Supplementary Figure 5.** Forest plot comparing EUS-GE and SGJ in mGOO patients for reintervention

**Supplementary Figure 6.** Forest plot comparing EUS-GE and SGJ in mGOO patients for recurrent obstruction

**Supplementary Figure 7.** Forest plots presenting subgroup analyses (randomized controlled trials and propensity score–matched cohort studies) for length of hospital stay comparing EUS-GE and SGJ in patients with mGOO

**Supplementary Figure 8.** Forest plot comparing EUS-GE and SGJ in mGOO patients for time to oral intake

**Supplementary Figure 9.** Forest plots presenting subgroup analyses (randomized controlled trials and propensity score–matched cohort studies) for technical success comparing EUS-GE and enteral setting (ES) in patients with mGOO

**Supplementary Figure 10.** Forest plots presenting subgroup analyses (randomized controlled trials and propensity score–matched cohort studies) for postoperative complications comparing EUS-GE and enteral setting (ES) in patients with mGOO

**Supplementary Figure 11.** Forest plot comparing EUS-GE and enteral stenting (ES) in mGOO patients for length of hospital stay

**Supplementary Figure 12.** Forest plots presenting subgroup analyses (randomized controlled trials and propensity score–matched cohort studies) for length of hospital stay comparing EUS-GE and enteral setting (ES) in patients with mGOO

**Supplementary Figure 13.** Risk-of-Bias for RCTs performed by Rob-2

**Supplementary Figure 14.** Risk-of-Bias for observational studies by Robins-I

**Supplementary Figure 15.** Leave-one-out of mortality comparing EUS-GE and SGJ

**Supplementary Figure 16.** Leave-one-out of reintervention comparing EUS-GE and SGJ

**Supplementary Figure 17.** Leave-one-out of length of hospital comparing EUS-GE and SGJ

**Supplementary Figure 18.** Leave-one-out of operative time comparing EUS-GE and SGJ

**Supplementary Figure 19.** Leave-one-out of postoperative complications comparing EUS-GE and enteral stenting

**Supplementary Figure 20.** Leave-one-out of length of hospital stay comparing EUS-GE and enteral stenting

**Supplementary Figure 21.** Baujat plot analysis of mortality comparing EUS-GE and SGJ

**Supplementary Figure 22.** Baujat plot analysis of reintervention comparing EUS-GE and SGJ

**Supplementary Figure 23.** Baujat plot analysis of length of hospital stay comparing EUS-GE and SGJ

**Supplementary Figure 24.** Baujat plot analysis of operative time comparing EUS-GE and SGJ

**Supplementary Figure 25.** Baujat plot analysis of postoperative complications comparing EUS-GE and enteral stenting

**Supplementary Figure 26.** Baujat plot analysis of length of hospital stay comparing EUS-GE and enteral stenting

**Supplementary Figure 27.** Funnel plot of technical success comparing EUS-GE and SGJ

**Supplementary Figure 28.** Funnel plot of clinical success comparing EUS-GE and SGJ

**Supplementary Figure 29.** Funnel plot of technical success comparing enteral stenting

**Supplementary Figure 30.** Funnel plot of clinical success comparing enteral stenting

**Supplementary Figure 31.** TSA comparing EUS-GE with SGJ for overall postoperative complications (all-grade Clavien-Dindo)

**Supplementary Figure 32.** TSA comparing EUS-GE with enteral stenting for reintervention

**Supplementary Table 1.** PRISMA 2020 Checklist

| **Section and Topic** | **Item #** | **Checklist item** | **Location where item is reported** |
| --- | --- | --- | --- |
| **TITLE** | | |  |
| Title | 1 | Identify the report as a systematic review. | Title page |
| **ABSTRACT** | | |  |
| Abstract | 2 | See the PRISMA 2020 for Abstracts checklist. | Supplementary Figure 2 |
| **INTRODUCTION** | | |  |
| Rationale | 3 | Describe the rationale for the review in the context of existing knowledge. | Pages 2-3 |
| Objectives | 4 | Provide an explicit statement of the objective(s) or question(s) the review addresses. | Page 3 |
| **METHODS** | | |  |
| Eligibility criteria | 5 | Specify the inclusion and exclusion criteria for the review and how studies were grouped for the syntheses. | Pages 3-4 |
| Information sources | 6 | Specify all databases, registers, websites, organisations, reference lists and other sources searched or consulted to identify studies. Specify the date when each source was last searched or consulted. | Page 5 |
| Search strategy | 7 | Present the full search strategies for all databases, registers and websites, including any filters and limits used. | Supplementary Table 3 |
| Selection process | 8 | Specify the methods used to decide whether a study met the inclusion criteria of the review, including how many reviewers screened each record and each report retrieved, whether they worked independently, and if applicable, details of automation tools used in the process. | Pages 4-5 |
| Data collection process | 9 | Specify the methods used to collect data from reports, including how many reviewers collected data from each report, whether they worked independently, any processes for obtaining or confirming data from study investigators, and if applicable, details of automation tools used in the process. | Page 5 |
| Data items | 10a | List and define all outcomes for which data were sought. Specify whether all results that were compatible with each outcome domain in each study were sought (e.g. for all measures, time points, analyses), and if not, the methods used to decide which results to collect. | Pages 6-7 |
|  | 10b | List and define all other variables for which data were sought (e.g. participant and intervention characteristics, funding sources). Describe any assumptions made about any missing or unclear information. | Pages 6-7 |
| Study risk of bias assessment | 11 | Specify the methods used to assess risk of bias in the included studies, including details of the tool(s) used, how many reviewers assessed each study and whether they worked independently, and if applicable, details of automation tools used in the process. | Pages 5-6 |
| Effect measures | 12 | Specify for each outcome the effect measure(s) (e.g. risk ratio, mean difference) used in the synthesis or presentation of results. | Pages 7-8 |
| Synthesis methods | 13a | Describe the processes used to decide which studies were eligible for each synthesis (e.g. tabulating the study intervention characteristics and comparing against the planned groups for each synthesis (item #5)). | Pages 7-8 |
|  | 13b | Describe any methods required to prepare the data for presentation or synthesis, such as handling of missing summary statistics, or data conversions. | Pages 7-8 |
|  | 13c | Describe any methods used to tabulate or visually display results of individual studies and syntheses. | Pages 7-8 |
|  | 13d | Describe any methods used to synthesize results and provide a rationale for the choice(s). If meta-analysis was performed, describe the model(s), method(s) to identify the presence and extent of statistical heterogeneity, and software package(s) used. | Pages 7-8 |
|  | 13e | Describe any methods used to explore possible causes of heterogeneity among study results (e.g. subgroup analysis, meta-regression). | Page 6 |
|  | 13f | Describe any sensitivity analyses conducted to assess robustness of the synthesized results. | Page 6 |
| Reporting bias assessment | 14 | Describe any methods used to assess risk of bias due to missing results in a synthesis (arising from reporting biases). | Pages 5-6 |
| Certainty assessment | 15 | Describe any methods used to assess certainty (or confidence) in the body of evidence for an outcome. | Page NO |
| **RESULTS** | | |  |
| Study selection | 16a | Describe the results of the search and selection process, from the number of records identified in the search to the number of studies included in the review, ideally using a flow diagram. | Pages 8-9  Figure 1 |
|  | 16b | Cite studies that might appear to meet the inclusion criteria, but which were excluded, and explain why they were excluded. | Pages 8-9  Figure 1 |
| Study characteristics | 17 | Cite each included study and present its characteristics. | Pages 9  Table 1 |
| Risk of bias in studies | 18 | Present assessments of risk of bias for each included study. | Pages 22-23  Supplementary Figures 17-18 |
| Results of individual studies | 19 | For all outcomes, present, for each study: (a) summary statistics for each group (where appropriate) and (b) an effect estimate and its precision (e.g. confidence/credible interval), ideally using structured tables or plots. | Pages 13-22 |
| Results of syntheses | 20a | For each synthesis, briefly summarise the characteristics and risk of bias among contributing studies. | Pages 22-23  Supplementary Figures 17-18 |
|  | 20b | Present results of all statistical syntheses conducted. If meta-analysis was done, present for each the summary estimate and its precision (e.g. confidence/credible interval) and measures of statistical heterogeneity. If comparing groups, describe the direction of the effect. | Pages 13-24 |
|  | 20c | Present results of all investigations of possible causes of heterogeneity among study results. | Pages 23-24 |
|  | 20d | Present results of all sensitivity analyses conducted to assess the robustness of the synthesized results. | Pages 23-24 |
| Reporting biases | 21 | Present assessments of risk of bias due to missing results (arising from reporting biases) for each synthesis assessed. | Pages 22-23  Supplementary Figures 17-18 |
| Certainty of evidence | 22 | Present assessments of certainty (or confidence) in the body of evidence for each outcome assessed. | Page NO |
| **DISCUSSION** | | |  |
| Discussion | 23a | Provide a general interpretation of the results in the context of other evidence. | Page 25 |
|  | 23b | Discuss any limitations of the evidence included in the review. | Page 28 |
|  | 23c | Discuss any limitations of the review processes used. | Page 28 |
|  | 23d | Discuss implications of the results for practice, policy, and future research. | Page 28 |
| **OTHER INFORMATION** | | |  |
| Registration and protocol | 24a | Provide registration information for the review, including register name and registration number, or state that the review was not registered. | Page 4 |
|  | 24b | Indicate where the review protocol can be accessed, or state that a protocol was not prepared. | Page 4 |
|  | 24c | Describe and explain any amendments to information provided at registration or in the protocol. | Page 4 |
| Support | 25 | Describe sources of financial or non-financial support for the review, and the role of the funders or sponsors in the review. | Page 29 |
| Competing interests | 26 | Declare any competing interests of review authors. | Page 29 |
| Availability of data, code and other materials | 27 | Report which of the following are publicly available and where they can be found: template data collection forms; data extracted from included studies; data used for all analyses; analytic code; any other materials used in the review. | Page NO |

**Supplementary Table 2.** PRISMA 2020 for Abstract Checklist

| **Section and Topic** | **Item #** | **Checklist item** | **Reported (Yes/No)** |
| --- | --- | --- | --- |
| **TITLE** | | |  |
| Title | 1 | Identify the report as a systematic review. | Yes |
| **BACKGROUND** | | |  |
| Objectives | 2 | Provide an explicit statement of the main objective(s) or question(s) the review addresses. | Yes |
| **METHODS** | | |  |
| Eligibility criteria | 3 | Specify the inclusion and exclusion criteria for the review. | No |
| Information sources | 4 | Specify the information sources (e.g. databases, registers) used to identify studies and the date when each was last searched. | Yes |
| Risk of bias | 5 | Specify the methods used to assess risk of bias in the included studies. | No |
| Synthesis of results | 6 | Specify the methods used to present and synthesise results. | Yes |
| **RESULTS** | | |  |
| Included studies | 7 | Give the total number of included studies and participants and summarise relevant characteristics of studies. | Yes |
| Synthesis of results | 8 | Present results for main outcomes, preferably indicating the number of included studies and participants for each. If meta-analysis was done, report the summary estimate and confidence/credible interval. If comparing groups, indicate the direction of the effect (i.e. which group is favoured). | Yes |
| **DISCUSSION** | | |  |
| Limitations of evidence | 9 | Provide a brief summary of the limitations of the evidence included in the review (e.g. study risk of bias, inconsistency and imprecision). | No |
| Interpretation | 10 | Provide a general interpretation of the results and important implications. | Yes |
| **OTHER** | | |  |
| Funding | 11 | Specify the primary source of funding for the review. | No |
| Registration | 12 | Provide the register name and registration number. | No |

**Supplementary Table 3.** Search Strategies

| **Database** | **Search Strategy** |
| --- | --- |
| **PubMed** | ("malignant gastric outlet obstruction” OR “neoplastic gastric outlet obstruction” OR “gastric outlet obstruction” OR “GOO” OR “pylorostenosis” OR “pyloric obstruction”) AND (“EUS-GE” OR “Endoscopic ultrasonography-guided gastroenterostomy” OR “EUS-guided gastroenterostomy” OR “endoscopic gastroenterostomy” OR “echoendoscopy” OR “echo-endoscopy” OR “endoscopic-echography” OR “EUS-guided” OR “ultrasound-guided”) AND (“gastroenterostomy” OR “gastroenteric anastomosis” OR “gastroenteroanastomosis” OR “surgical gastroenterostomy” OR "gastro-enterostomy” OR "SGE” OR “laparoscopic gastroenterostomy” OR “L-GE” OR “Surgical gastrojejunostomy” OR “gastrojejunostomy” OR “gastro-jejunostomy” OR “SGJ” OR “gastroduodenostomy” OR "gastro-duodenostomy” OR “bypass surgery” OR “gastrojejunostomy bypass” OR “endoscopic enteral stenting” OR “EES” OR ”duodenal stent placement” OR “enteral stenting” OR “endoscopic stenting” OR “enteral stent” OR "duodenal stent") |
| **Embase** | ('malignant gastric outlet obstruction':ti,ab OR 'neoplastic gastric outlet obstruction':ti,ab OR 'gastric outlet obstruction':ti,ab OR 'GOO':ti,ab OR 'pylorostenosis':ti,ab OR 'pyloric obstruction':ti,ab) AND ('EUS-GE':ti,ab OR 'Endoscopic ultrasonography-guided gastroenterostomy':ti,ab OR 'EUS-guided gastroenterostomy':ti,ab OR 'endoscopic gastroenterostomy':ti,ab OR 'echoendoscopy':ti,ab OR 'echo-endoscopy':ti,ab OR 'endoscopic-echography':ti,ab OR 'EUS-guided':ti,ab OR 'ultrasound-guided':ti,ab)  AND ('gastroenterostomy':ti,ab OR 'gastroenteric anastomosis':ti,ab OR 'gastroenteroanastomosis':ti,ab OR 'surgical gastroenterostomy':ti,ab OR 'gastro-enterostomy':ti,ab OR 'SGE':ti,ab OR 'laparoscopic gastroenterostomy':ti,ab OR 'L-GE':ti,ab OR 'Surgical gastrojejunostomy':ti,ab OR 'gastrojejunostomy':ti,ab OR 'gastro-jejunostomy':ti,ab OR 'SGJ':ti,ab OR 'gastroduodenostomy':ti,ab OR 'gastro-duodenostomy':ti,ab OR 'bypass surgery':ti,ab OR 'gastrojejunostomy bypass':ti,ab OR 'endoscopic enteral stenting':ti,ab OR 'EES':ti,ab OR 'duodenal stent placement':ti,ab OR 'enteral stenting':ti,ab OR 'endoscopic stenting':ti,ab OR 'enteral stent':ti,ab OR 'duodenal stent':ti,ab) |
| **Scopus** | (TITLE-ABS-KEY("malignant gastric outlet obstruction") OR TITLE-ABS-KEY("neoplastic gastric outlet obstruction") OR TITLE-ABS-KEY("gastric outlet obstruction") OR TITLE-ABS-KEY("GOO") OR TITLE-ABS-KEY("pylorostenosis") OR TITLE-ABS-KEY("pyloric obstruction")) AND (TITLE-ABS-KEY("EUS-GE") OR TITLE-ABS-KEY("Endoscopic ultrasonography-guided gastroenterostomy") OR TITLE-ABS-KEY("EUS-guided gastroenterostomy") OR TITLE-ABS-KEY("endoscopic gastroenterostomy") OR TITLE-ABS-KEY("echoendoscopy") OR TITLE-ABS-KEY("echo-endoscopy") OR TITLE-ABS-KEY("endoscopic-echography") OR TITLE-ABS-KEY("EUS-guided") OR TITLE-ABS-KEY("ultrasound-guided")) AND (TITLE-ABS-KEY("gastroenterostomy") OR TITLE-ABS-KEY("gastroenteric anastomosis") OR TITLE-ABS-KEY("gastroenteroanastomosis") OR TITLE-ABS-KEY("surgical gastroenterostomy") OR TITLE-ABS-KEY("gastro-enterostomy") OR TITLE-ABS-KEY("SGE") OR TITLE-ABS-KEY("laparoscopic gastroenterostomy") OR TITLE-ABS-KEY("L-GE") OR TITLE-ABS-KEY("Surgical gastrojejunostomy") OR TITLE-ABS-KEY("gastrojejunostomy") OR TITLE-ABS-KEY("gastro-jejunostomy") OR TITLE-ABS-KEY("SGJ") OR TITLE-ABS-KEY("gastroduodenostomy") OR TITLE-ABS-KEY("gastro-duodenostomy") OR TITLE-ABS-KEY("bypass surgery") OR TITLE-ABS-KEY("gastrojejunostomy bypass") OR TITLE-ABS-KEY("endoscopic enteral stenting") OR TITLE-ABS-KEY("EES") OR TITLE-ABS-KEY("duodenal stent placement") OR TITLE-ABS-KEY("enteral stenting") OR TITLE-ABS-KEY("endoscopic stenting") OR TITLE-ABS-KEY("enteral stent") OR TITLE-ABS-KEY("duodenal stent")) |
| **Cochrane** | (("malignant gastric outlet obstruction"):ti,ab,kw OR ("neoplastic gastric outlet obstruction"):ti,ab,kw OR ("gastric outlet obstruction"):ti,ab,kw OR ("GOO"):ti,ab,kw OR ("pylorostenosis"):ti,ab,kw OR ("pyloric obstruction"):ti,ab,kw) AND (("EUS-GE"):ti,ab,kw OR ("Endoscopic ultrasonography-guided gastroenterostomy"):ti,ab,kw OR ("EUS-guided gastroenterostomy"):ti,ab,kw OR ("endoscopic gastroenterostomy"):ti,ab,kw OR ("echoendoscopy"):ti,ab,kw OR ("echo-endoscopy"):ti,ab,kw OR ("endoscopic-echography"):ti,ab,kw OR ("EUS-guided"):ti,ab,kw OR ("ultrasound-guided"):ti,ab,kw) AND (("gastroenterostomy"):ti,ab,kw OR ("gastroenteric anastomosis"):ti,ab,kw OR ("gastroenteroanastomosis"):ti,ab,kw OR ("surgical gastroenterostomy"):ti,ab,kw OR ("gastro-enterostomy"):ti,ab,kw OR ("SGE"):ti,ab,kw OR ("laparoscopic gastroenterostomy"):ti,ab,kw OR ("L-GE"):ti,ab,kw OR ("Surgical gastrojejunostomy"):ti,ab,kw OR ("gastrojejunostomy"):ti,ab,kw OR ("gastro-jejunostomy"):ti,ab,kw OR ("SGJ"):ti,ab,kw OR ("gastroduodenostomy"):ti,ab,kw OR ("gastro-duodenostomy"):ti,ab,kw OR ("bypass surgery"):ti,ab,kw OR ("gastrojejunostomy bypass"):ti,ab,kw OR ("endoscopic enteral stenting"):ti,ab,kw OR ("EES"):ti,ab,kw OR ("duodenal stent placement"):ti,ab,kw OR ("enteral stenting"):ti,ab,kw OR ("endoscopic stenting"):ti,ab,kw OR ("enteral stent"):ti,ab,kw OR ("duodenal stent"):ti,ab,kw) |

**Supplementary Figure 1.** Forest plot comparing EUS-GE and SGJ in mGOO patients for postoperative sepsis


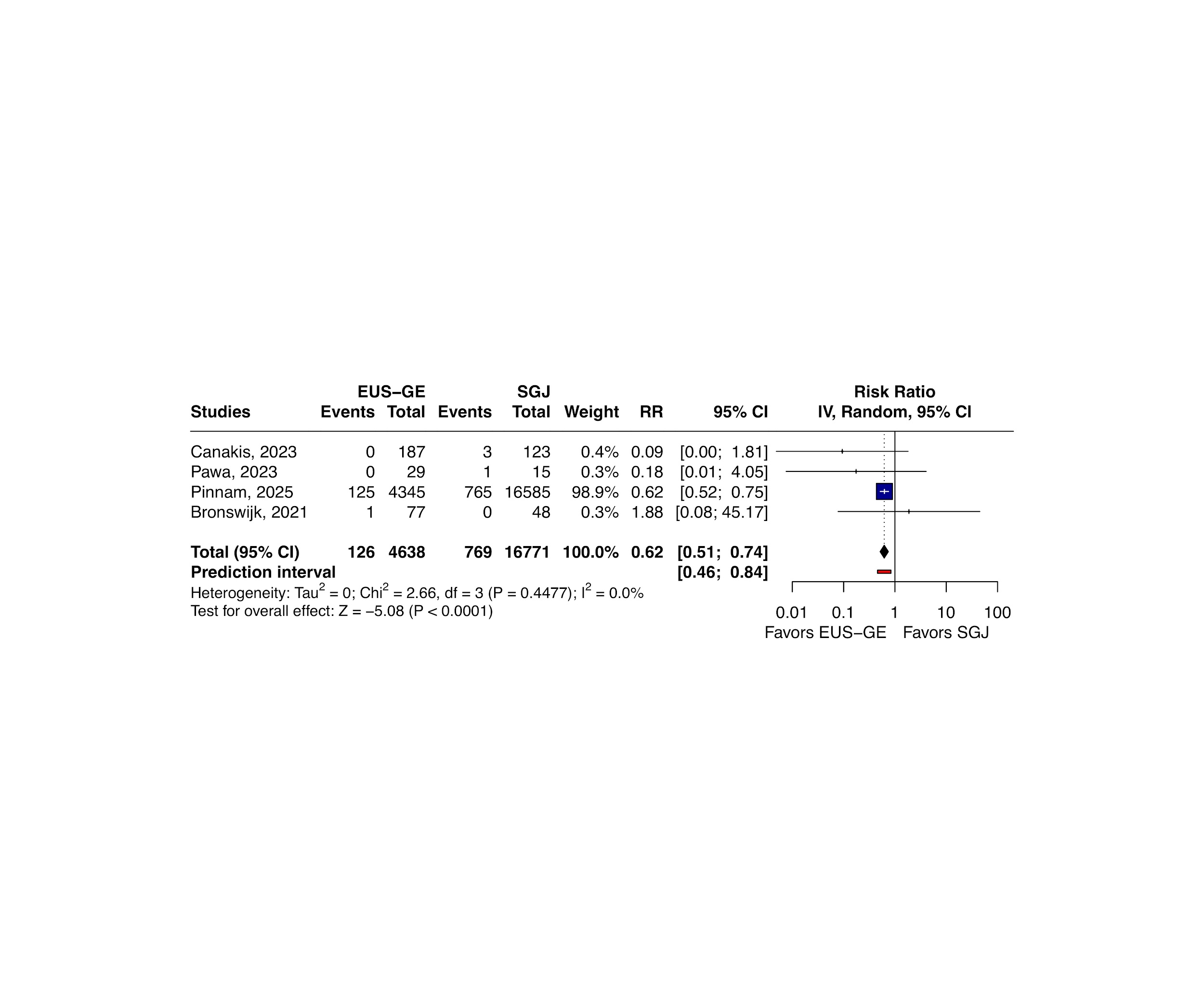


**Supplementary Figure 2.** Forest plot comparing EUS-GE and SGJ in mGOO patients for anastomotic leak


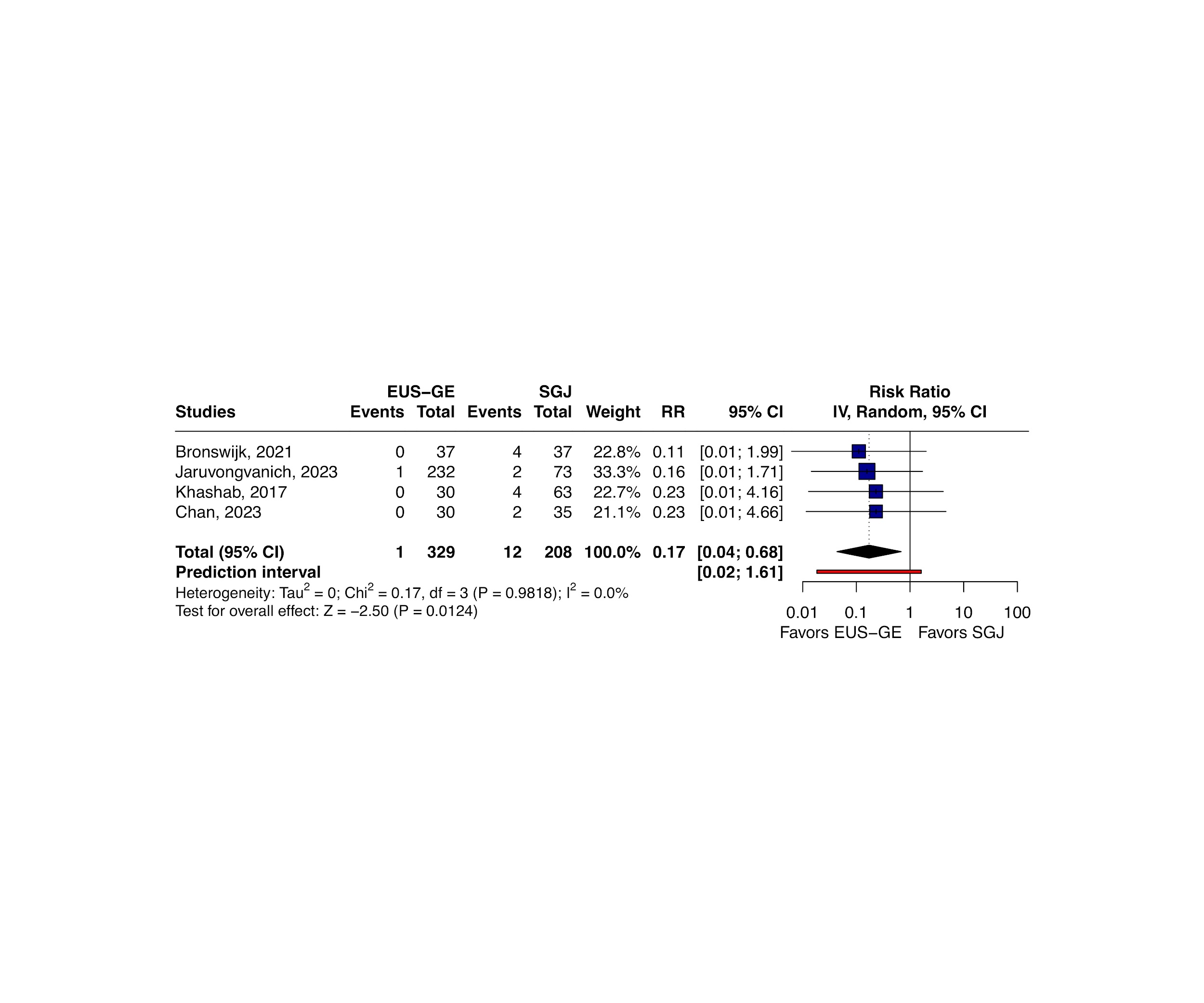


**Supplementary Figure 3.** Forest plot comparing EUS-GE and SGJ in mGOO patients for perforation


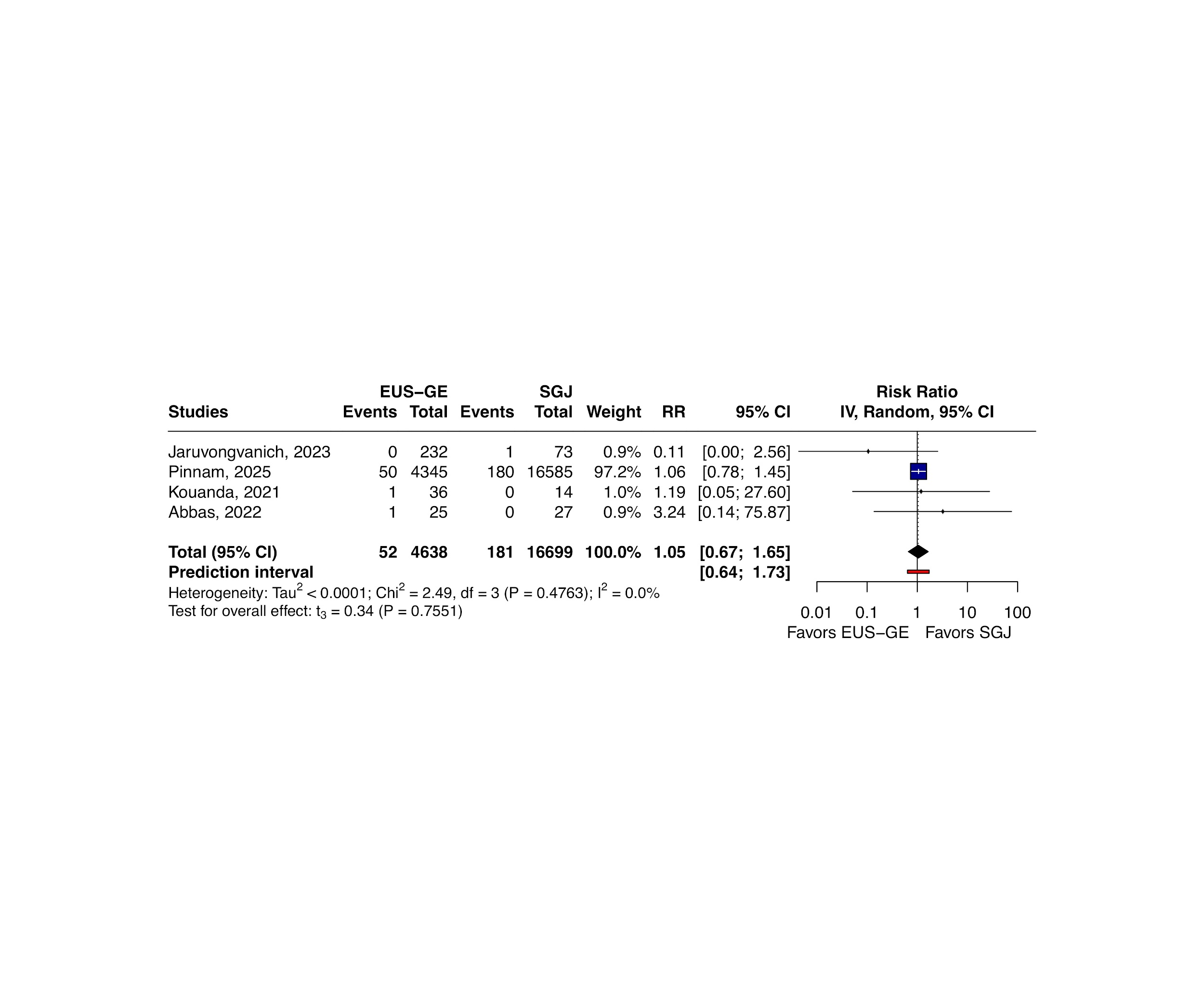


**Supplementary Figure 4.** Forest plots comparing EUS-GE and SGJ in mGOO patients for mortality across different time intervals (30-day, follow-up, and inpatient)


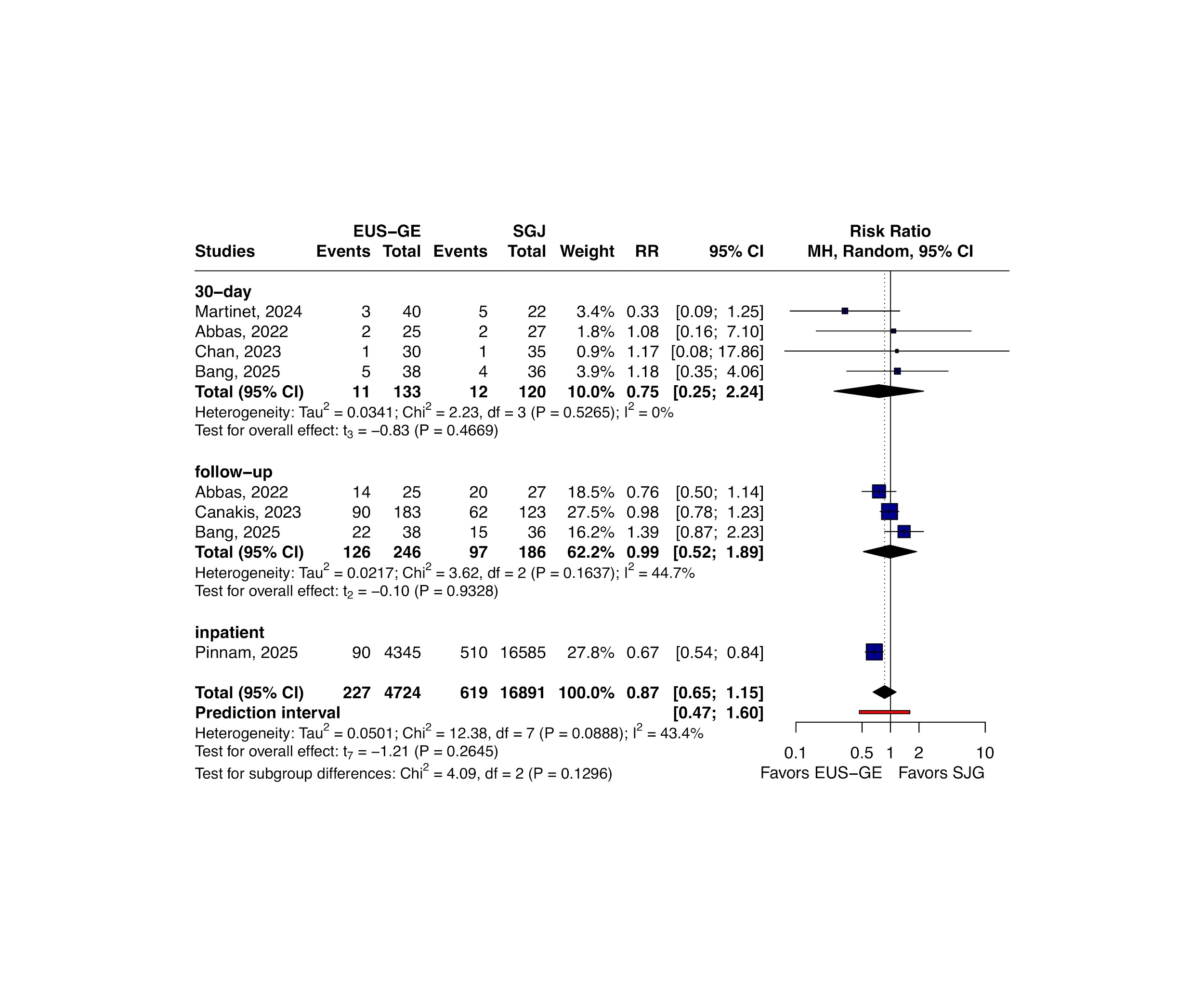


**Supplementary Figure 5.** Forest plot comparing EUS-GE and SGJ in mGOO patients for reintervention


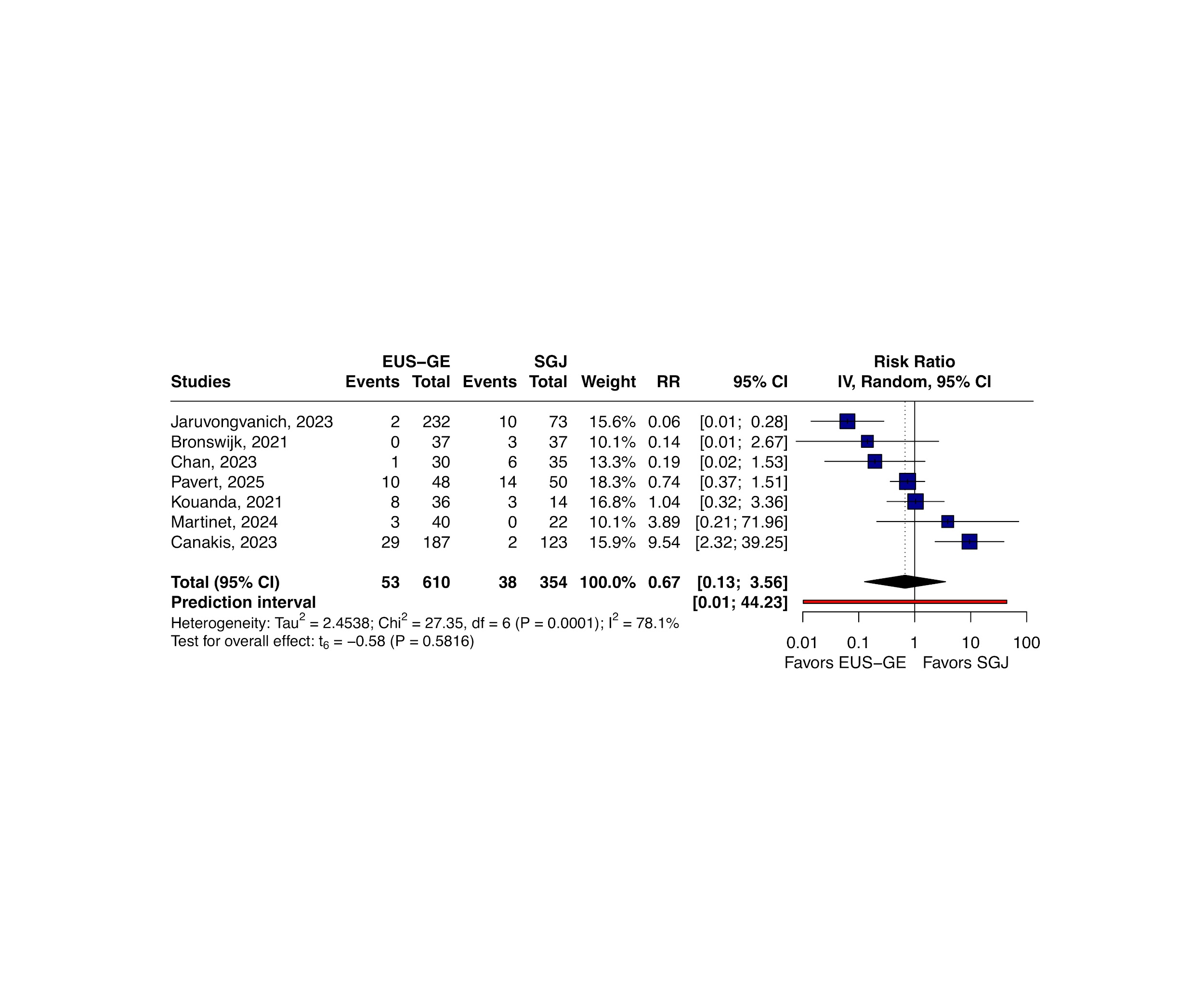


**Supplementary Figure 6.** Forest plot comparing EUS-GE and SGJ in mGOO patients for recurrent obstruction


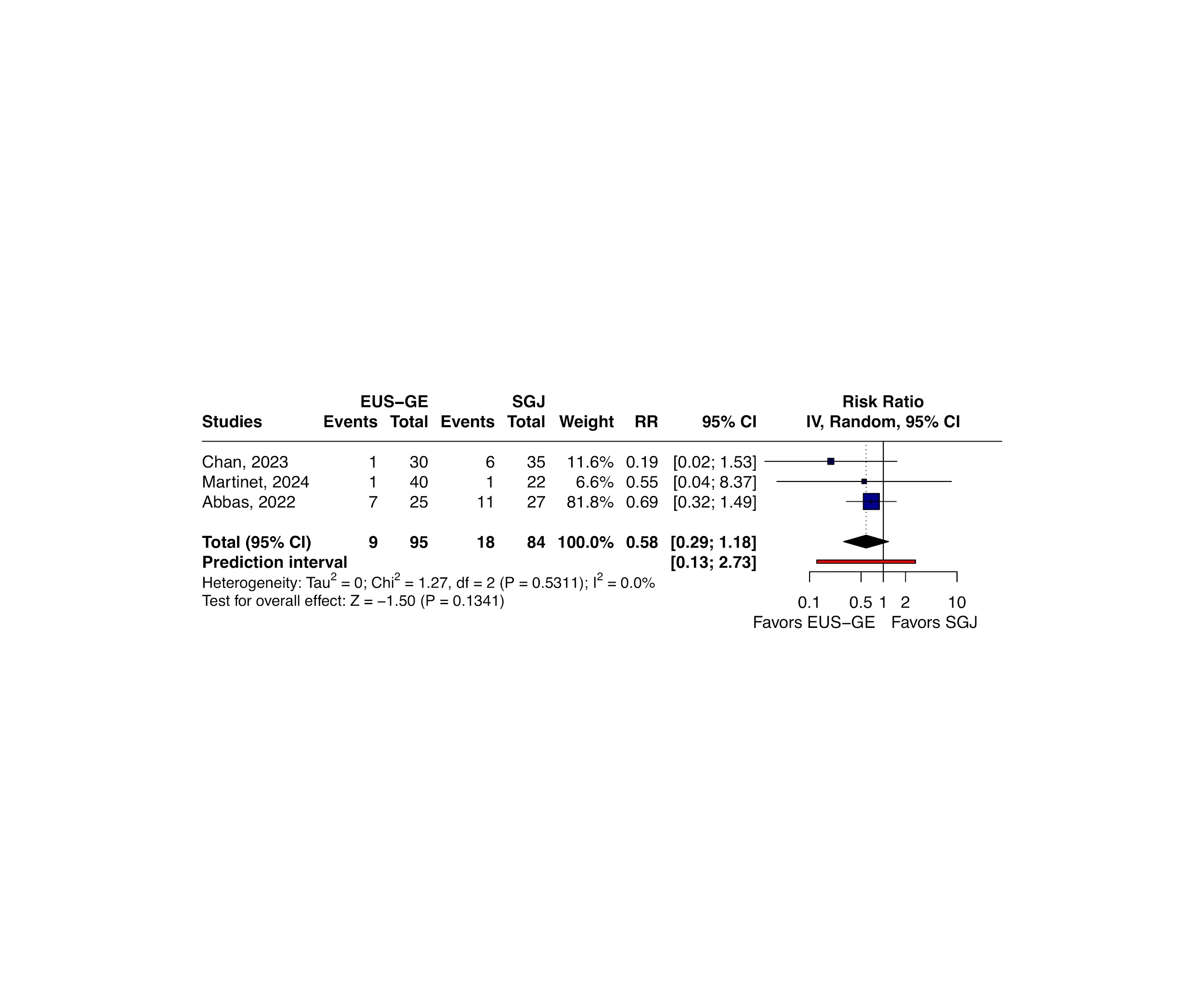


**Supplementary Figure 7.** Forest plots presenting subgroup analyses (randomized controlled trials and propensity score–matched cohort studies) for length of hospital stay comparing EUS-GE and SGJ in patients with mGOO


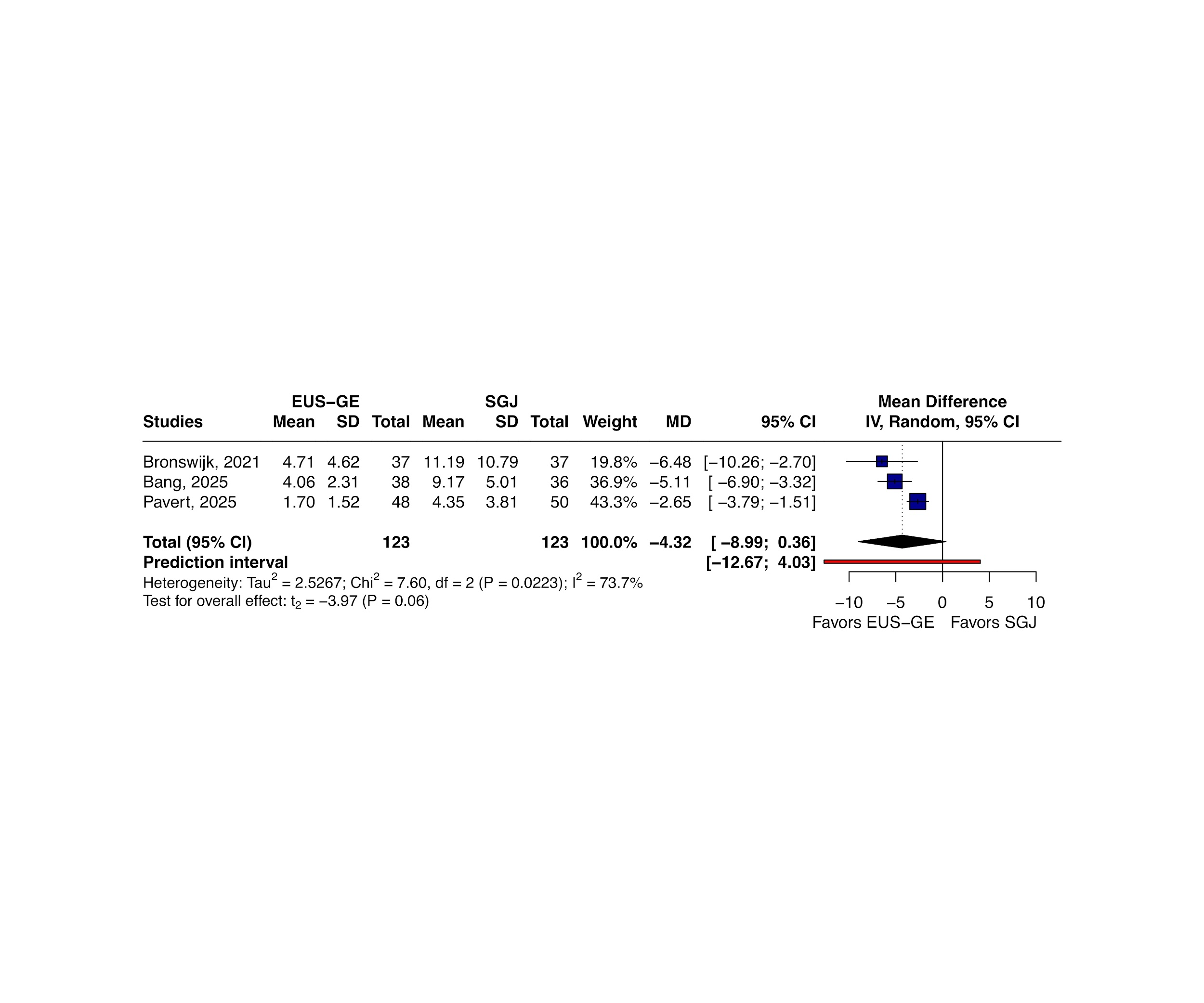


**Supplementary Figure 8.** Forest plot comparing EUS-GE and SGJ in mGOO patients for time to oral intake


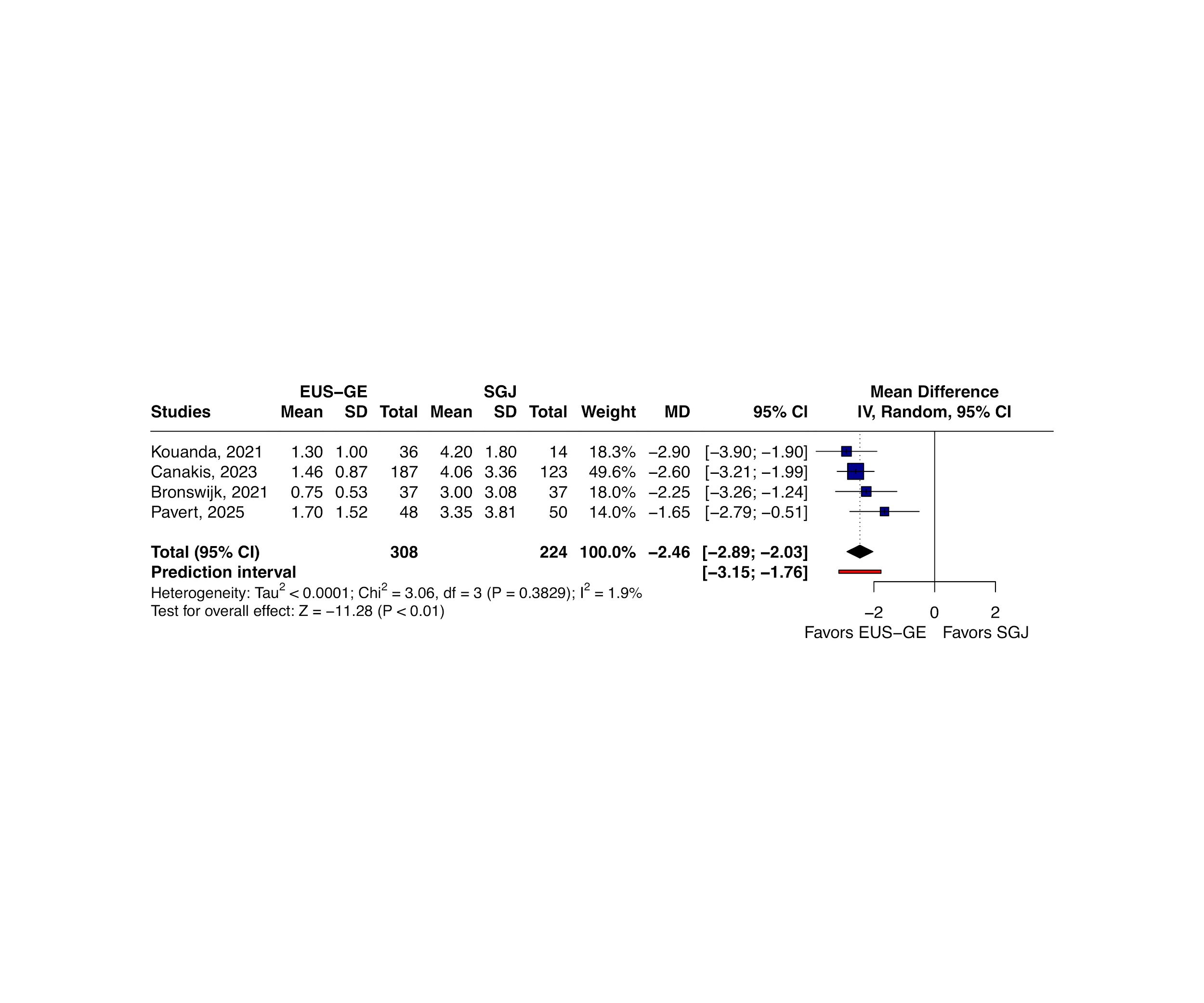


**Supplementary Figure 9.** Forest plots presenting subgroup analyses (randomized controlled trials and propensity score–matched cohort studies) for technical success comparing EUS-GE and enteral setting (ES) in patients with mGOO


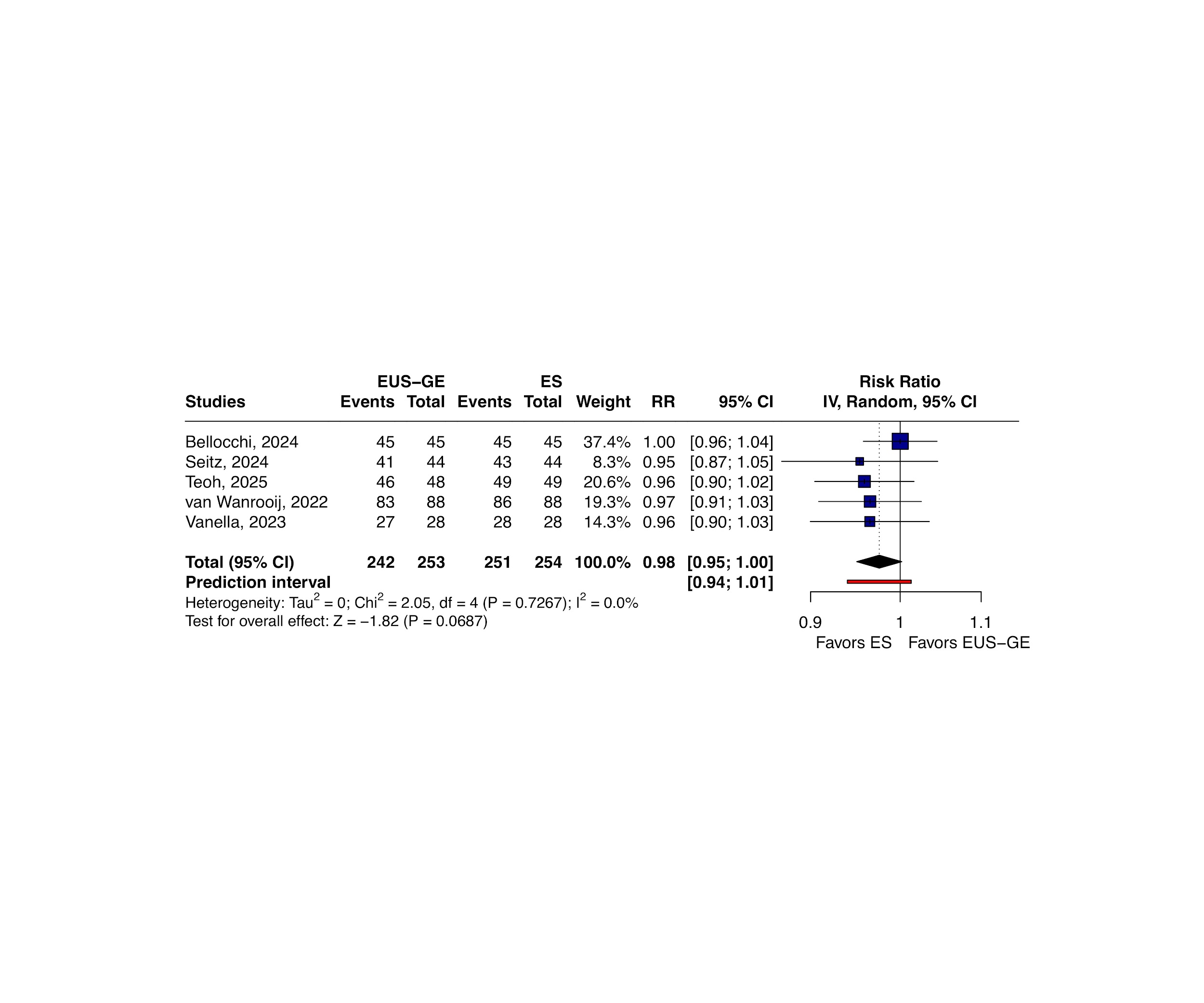


**Supplementary Figure 10.** Forest plots presenting subgroup analyses (randomized controlled trials and propensity score–matched cohort studies) for postoperative complications comparing EUS-GE and enteral setting (ES) in patients with mGOO


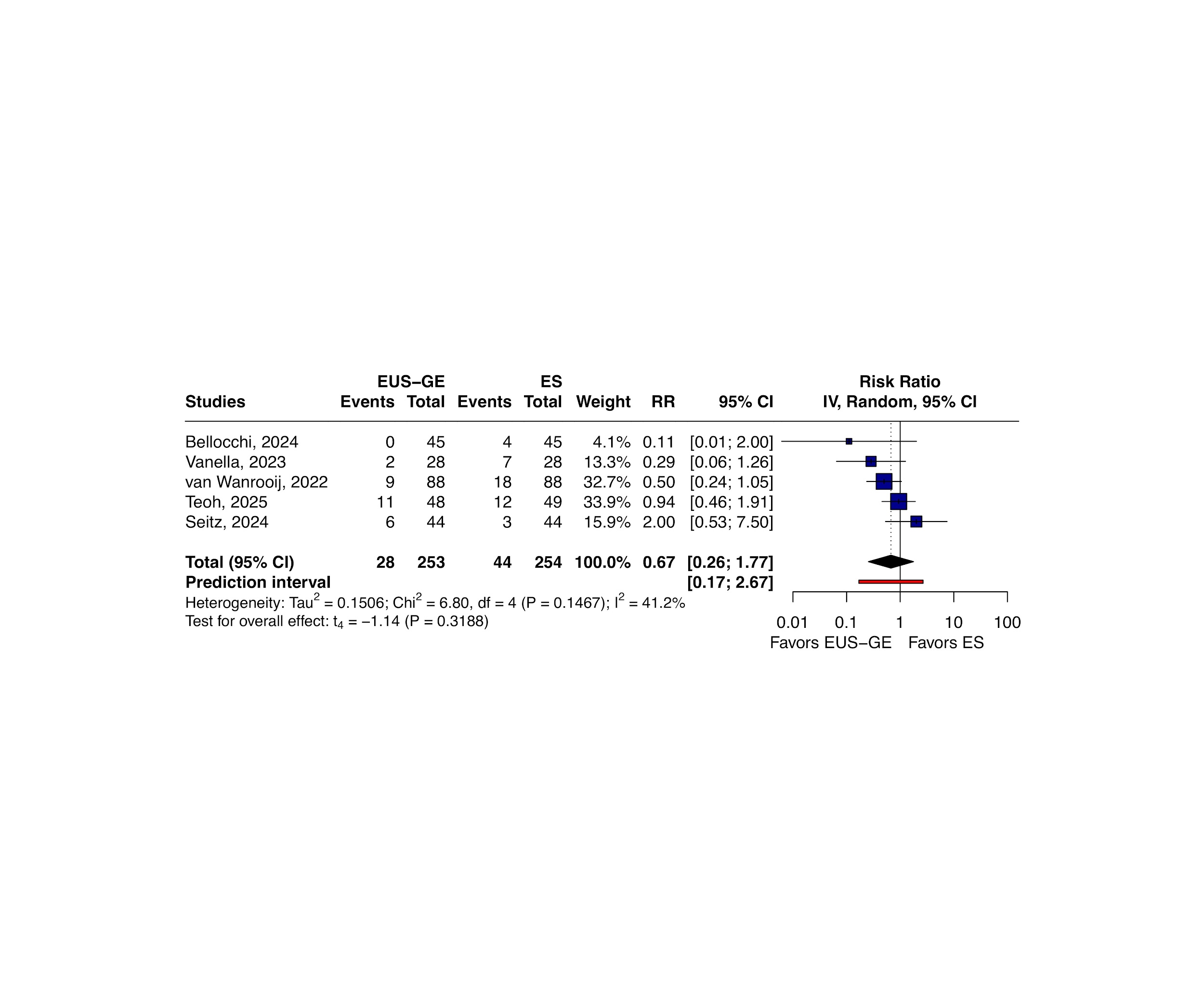


**Supplementary Figure 11.** Forest plot comparing EUS-GE and enteral stenting (ES) in mGOO patients for length of hospital stay


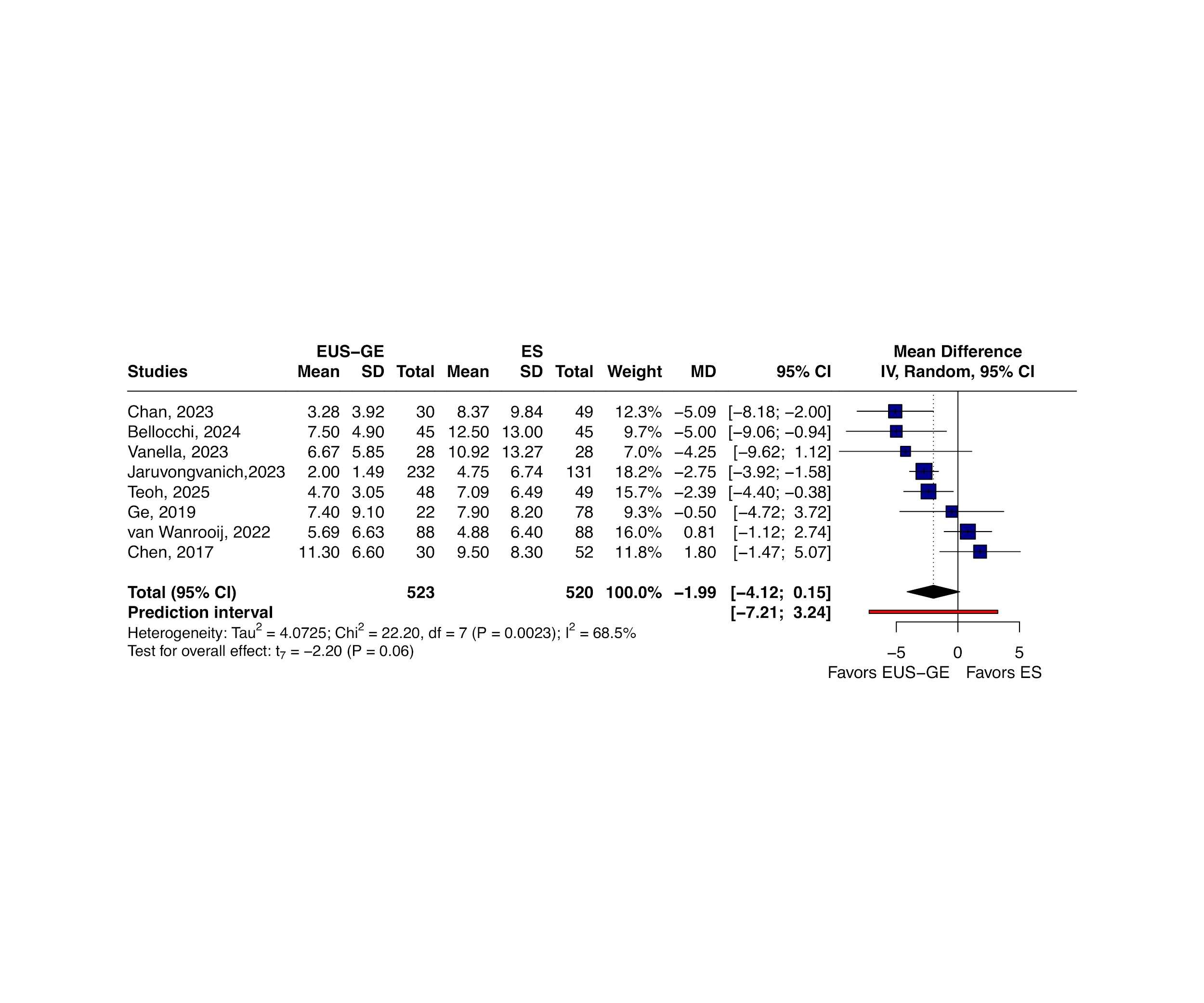


**Supplementary Figure 12.** Forest plots presenting subgroup analyses (randomized controlled trials and propensity score–matched cohort studies) for length of hospital stay comparing EUS-GE and enteral setting (ES) in patients with mGOO


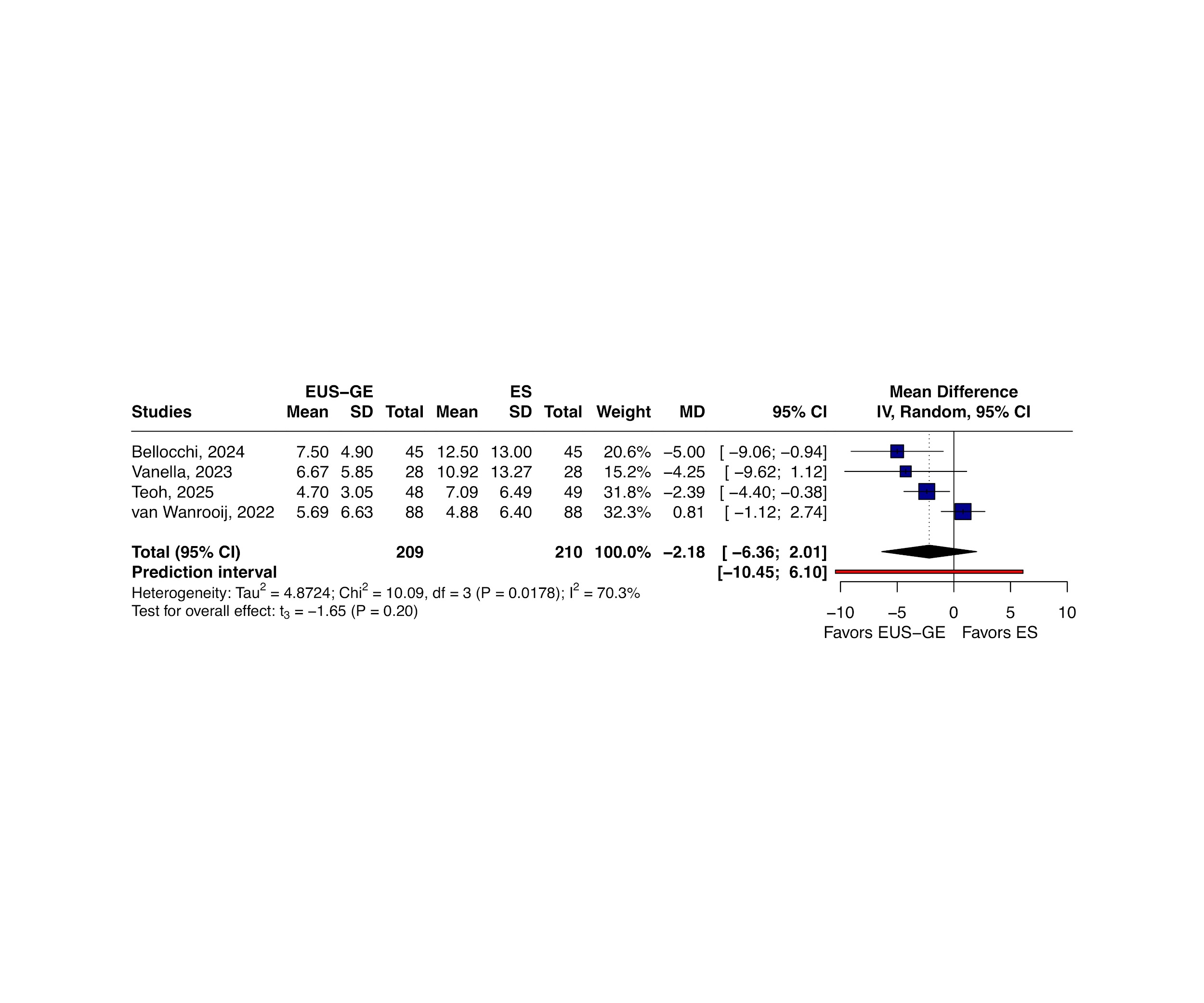


**Supplementary Figure 13.** Risk-of-Bias for RCTs performed by Rob-2


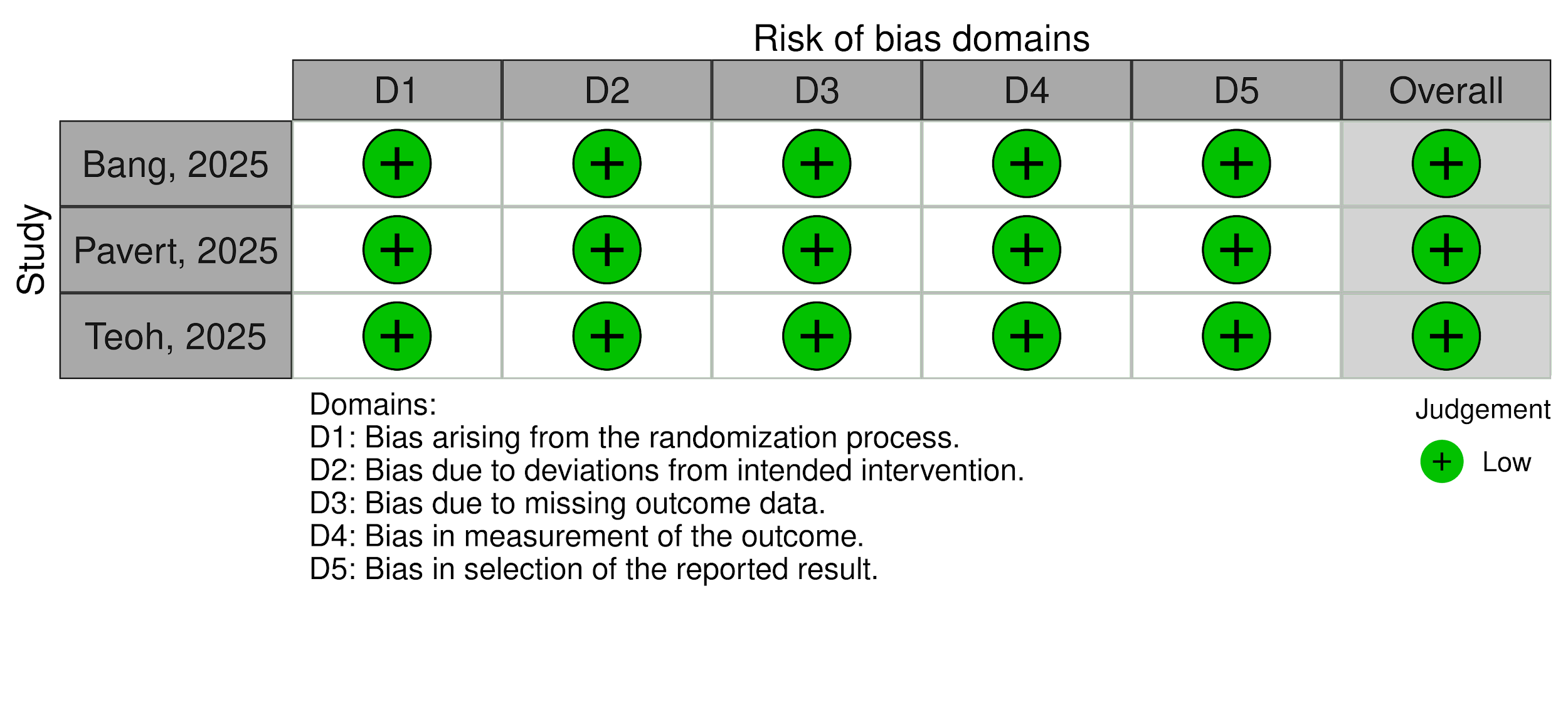


**Supplementary Figure 14.** Risk-of-Bias for observational studies by Robins-I


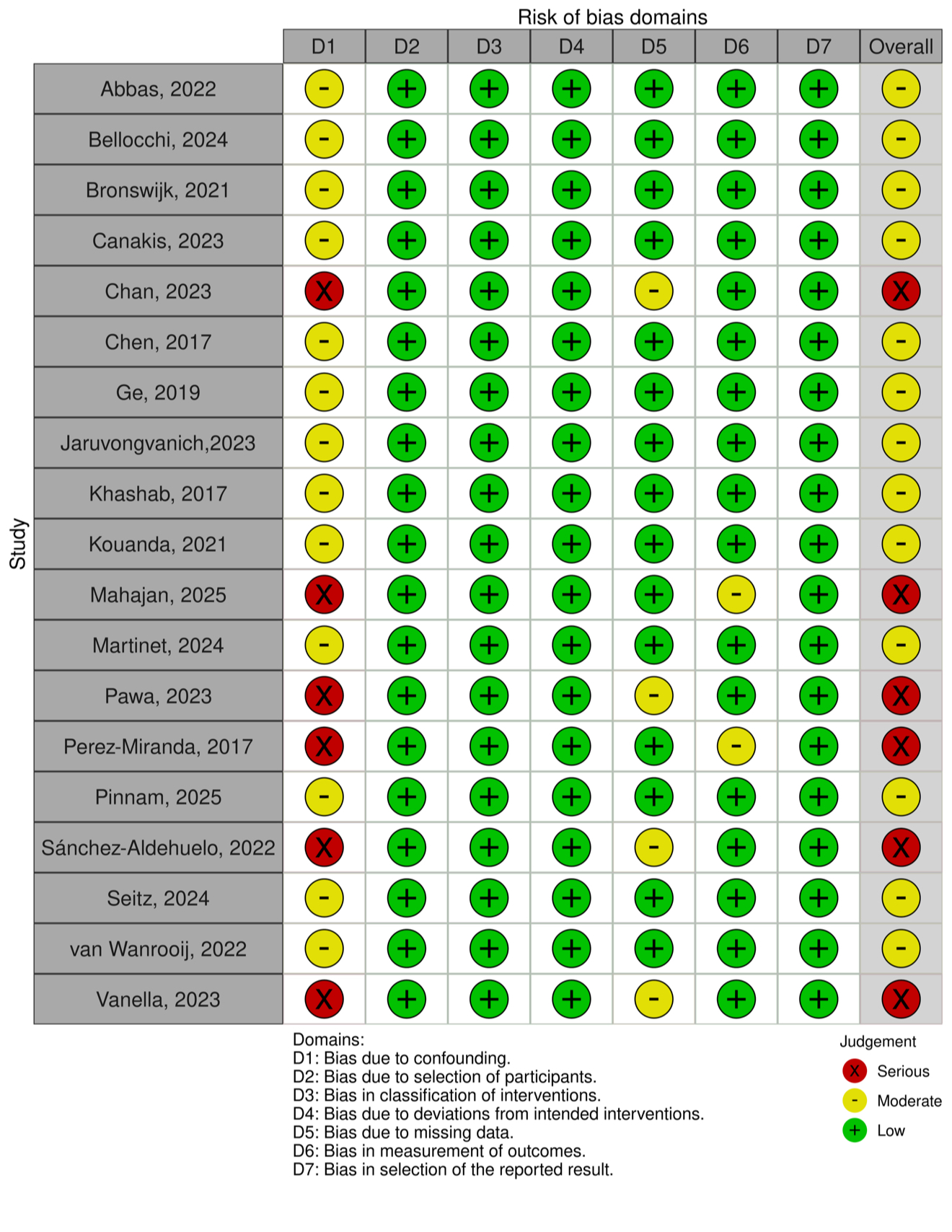


**Supplementary Figure 15.** Leave-one-out of mortality comparing EUS-GE and SGJ


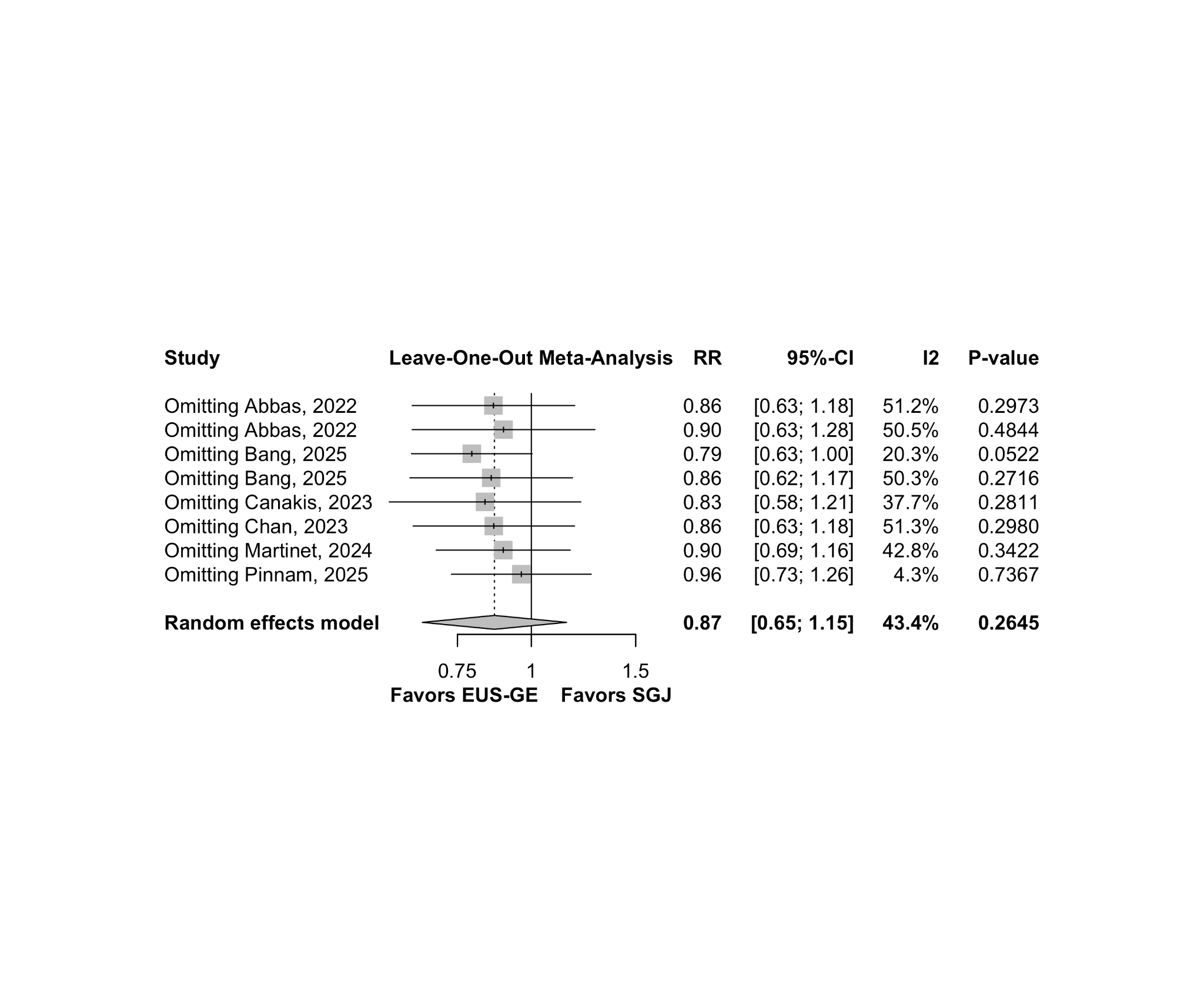


**Supplementary Figure 16.** Leave-one-out of reintervention comparing EUS-GE and SGJ


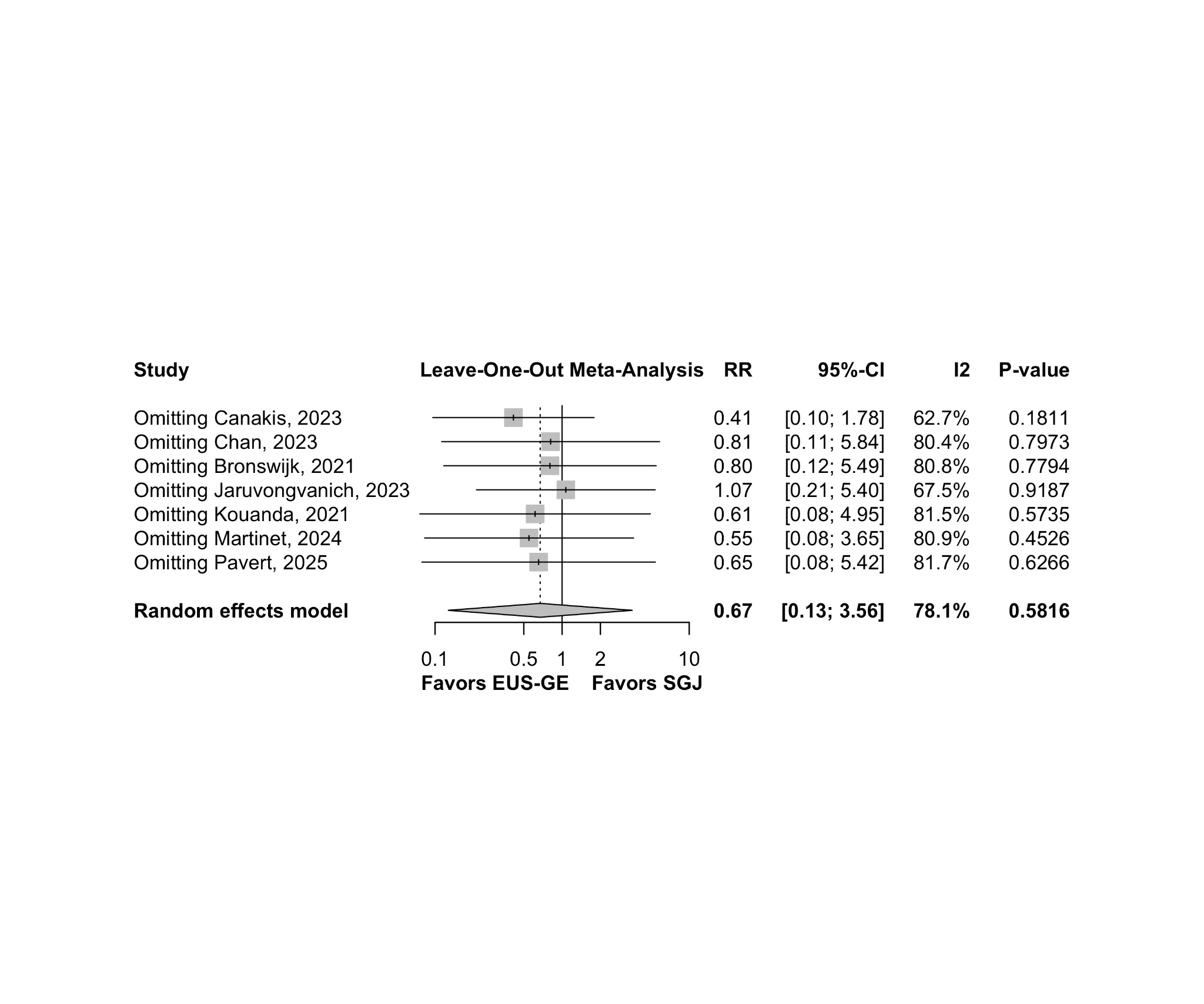


**Supplementary Figure 17.** Leave-one-out of length of hospital comparing EUS-GE and SGJ


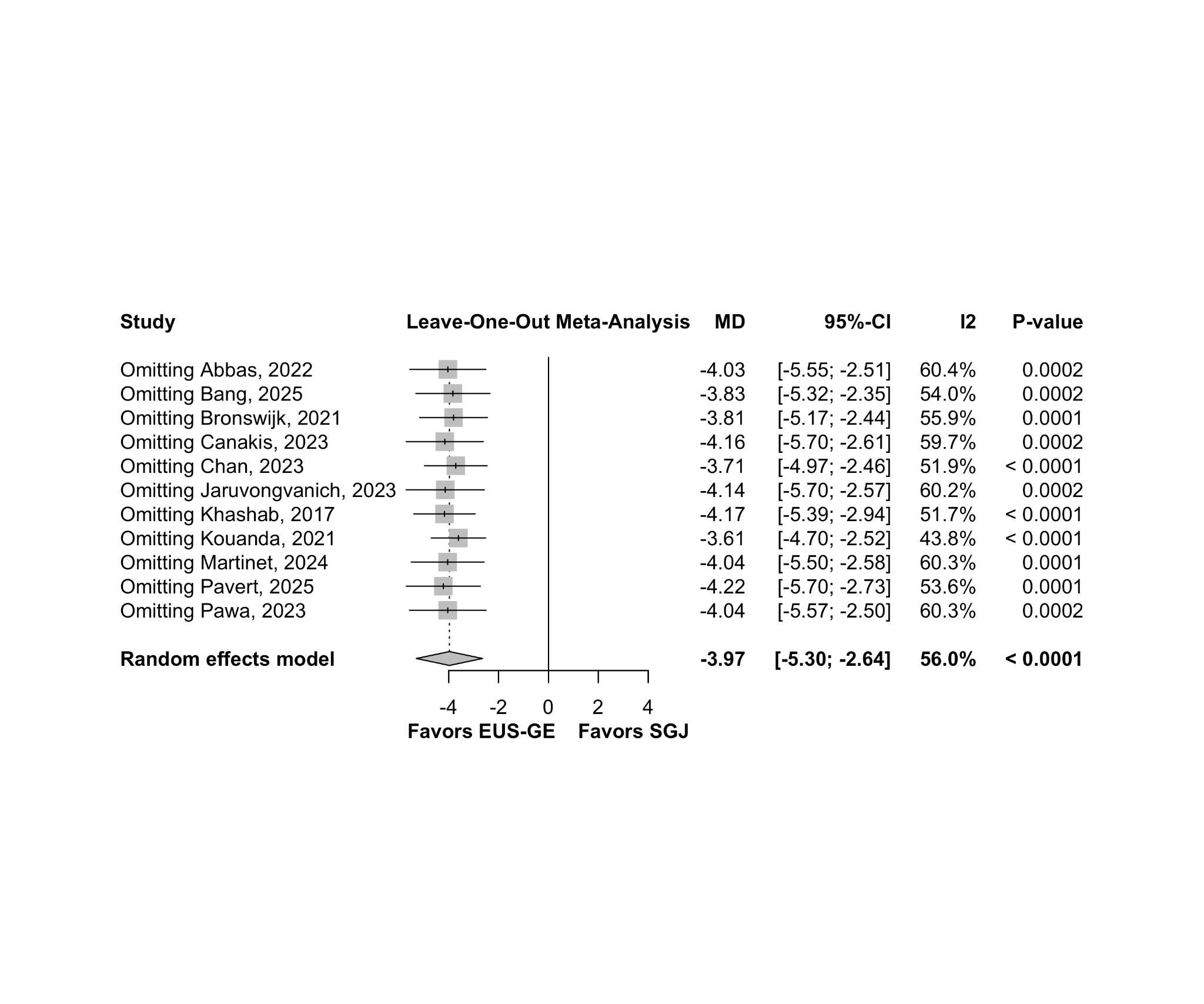


**Supplementary Figure 18.** Leave-one-out of operative time comparing EUS-GE and SGJ


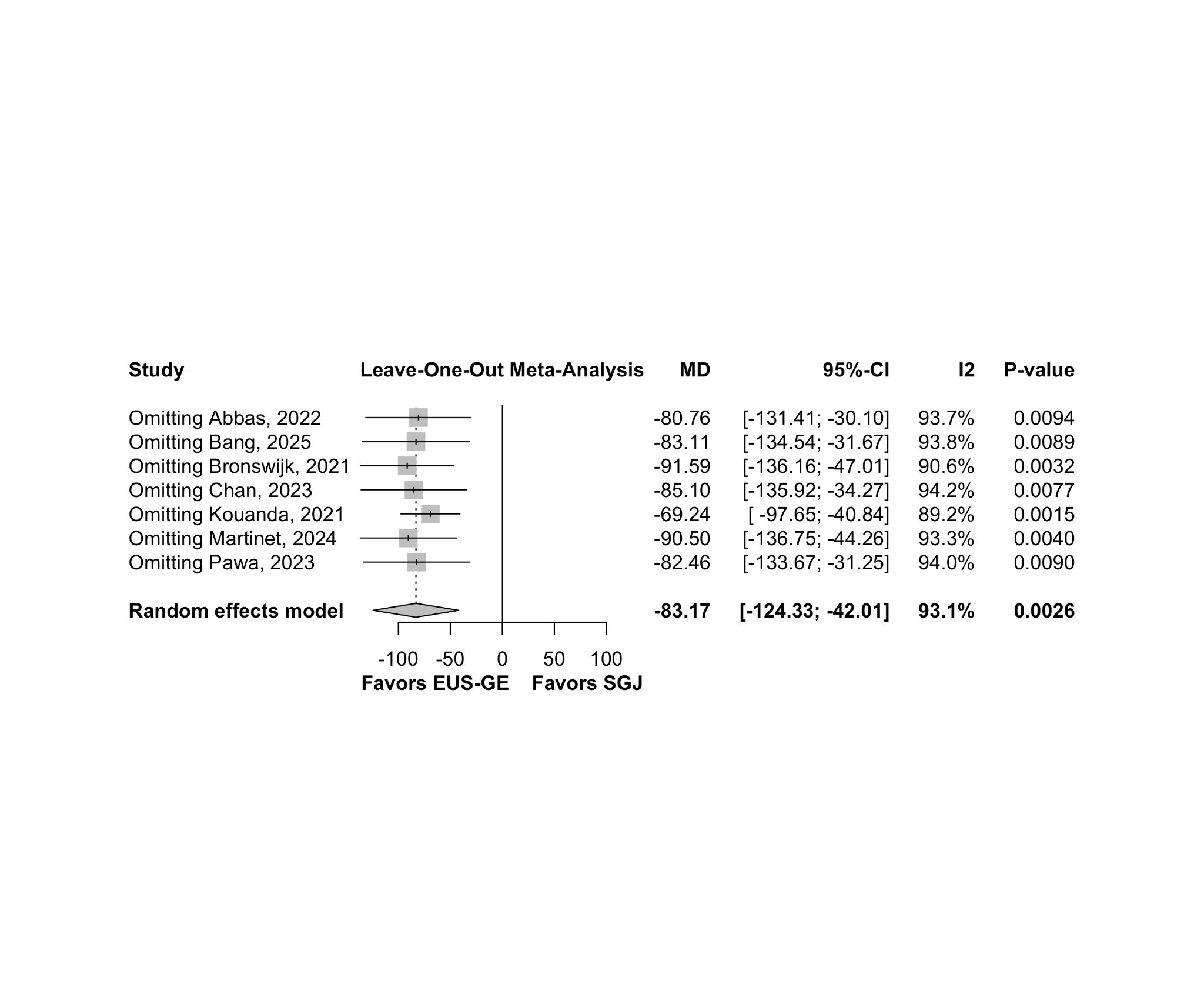


**Supplementary Figure 19.** Leave-one-out of postoperative complications comparing EUS-GE and enteral stenting


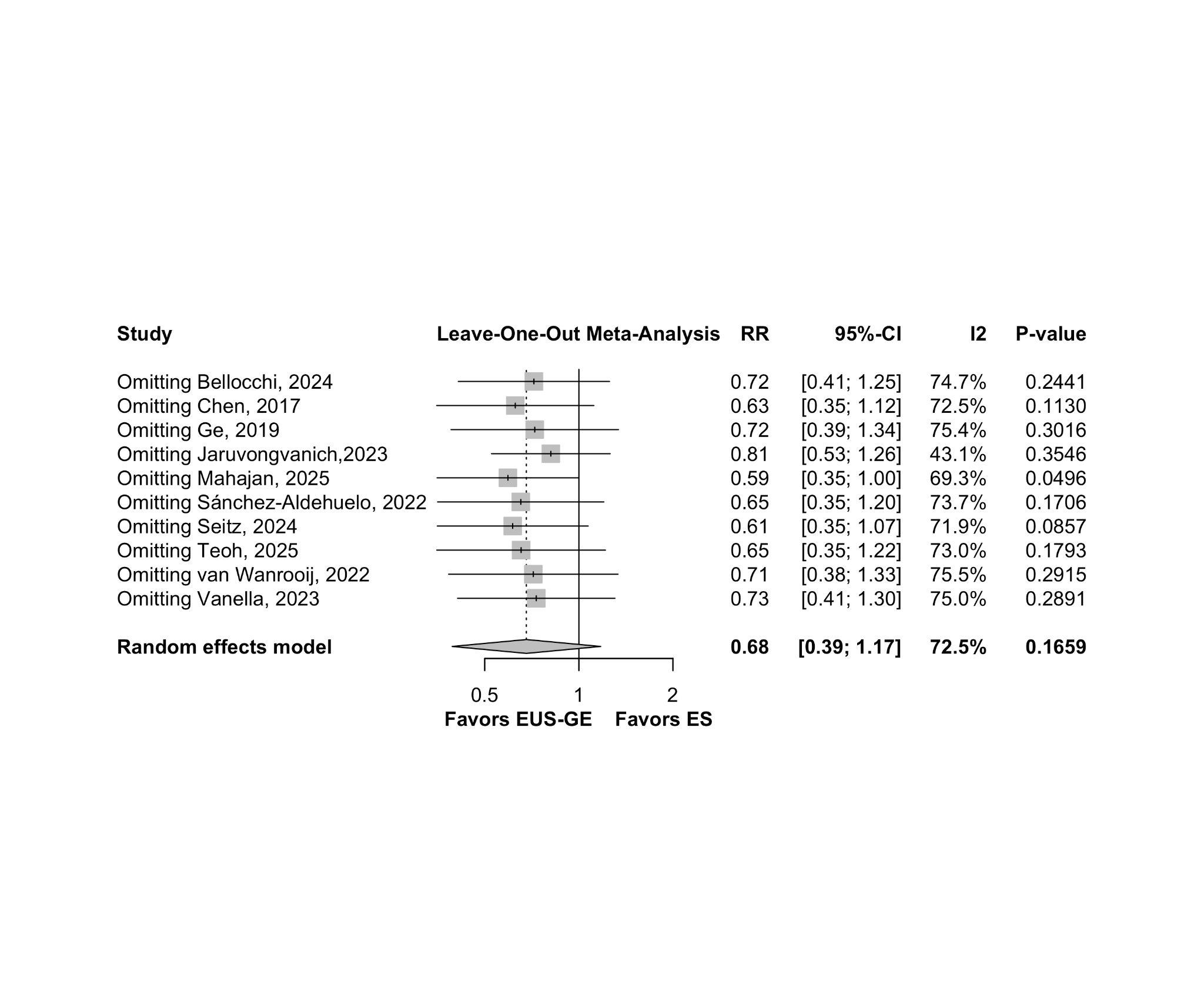


**Supplementary Figure 20.** Leave-one-out of length of hospital stay comparing EUS-GE and enteral stenting


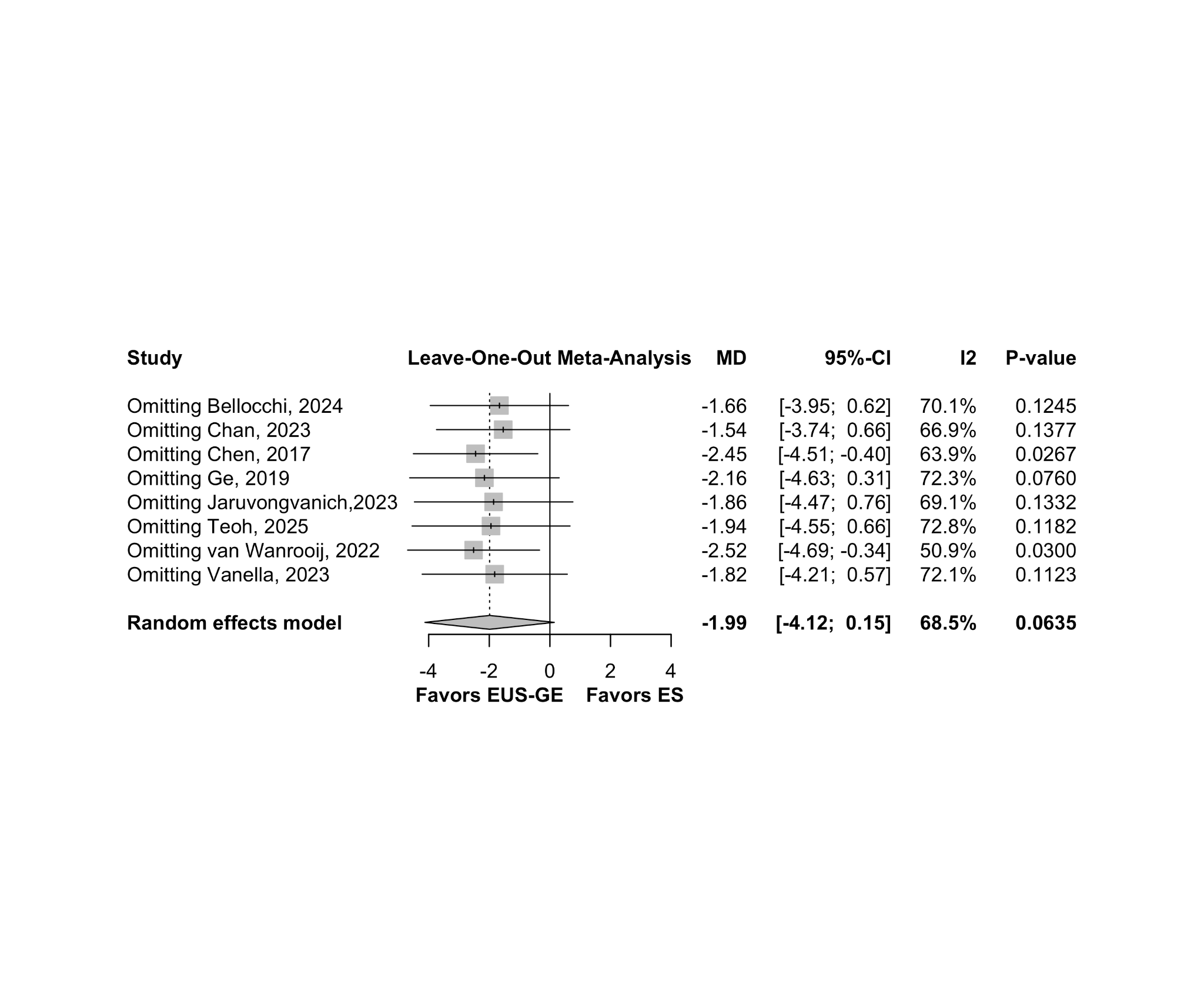


**Supplementary Figure 21.** Baujat plot analysis of mortality comparing EUS-GE and SGJ


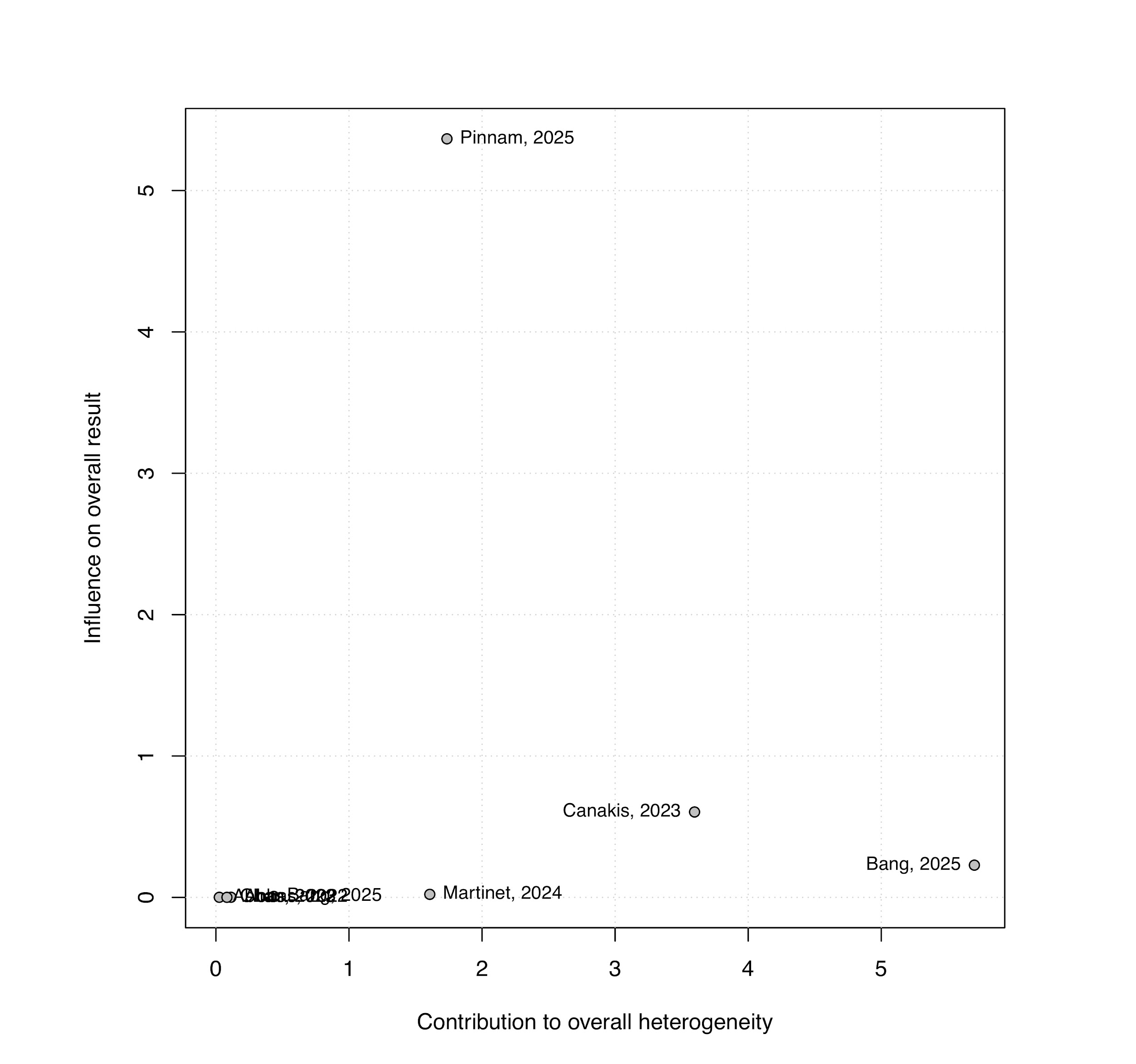


**Supplementary Figure 22.** Baujat plot analysis of reintervention comparing EUS-GE and SGJ


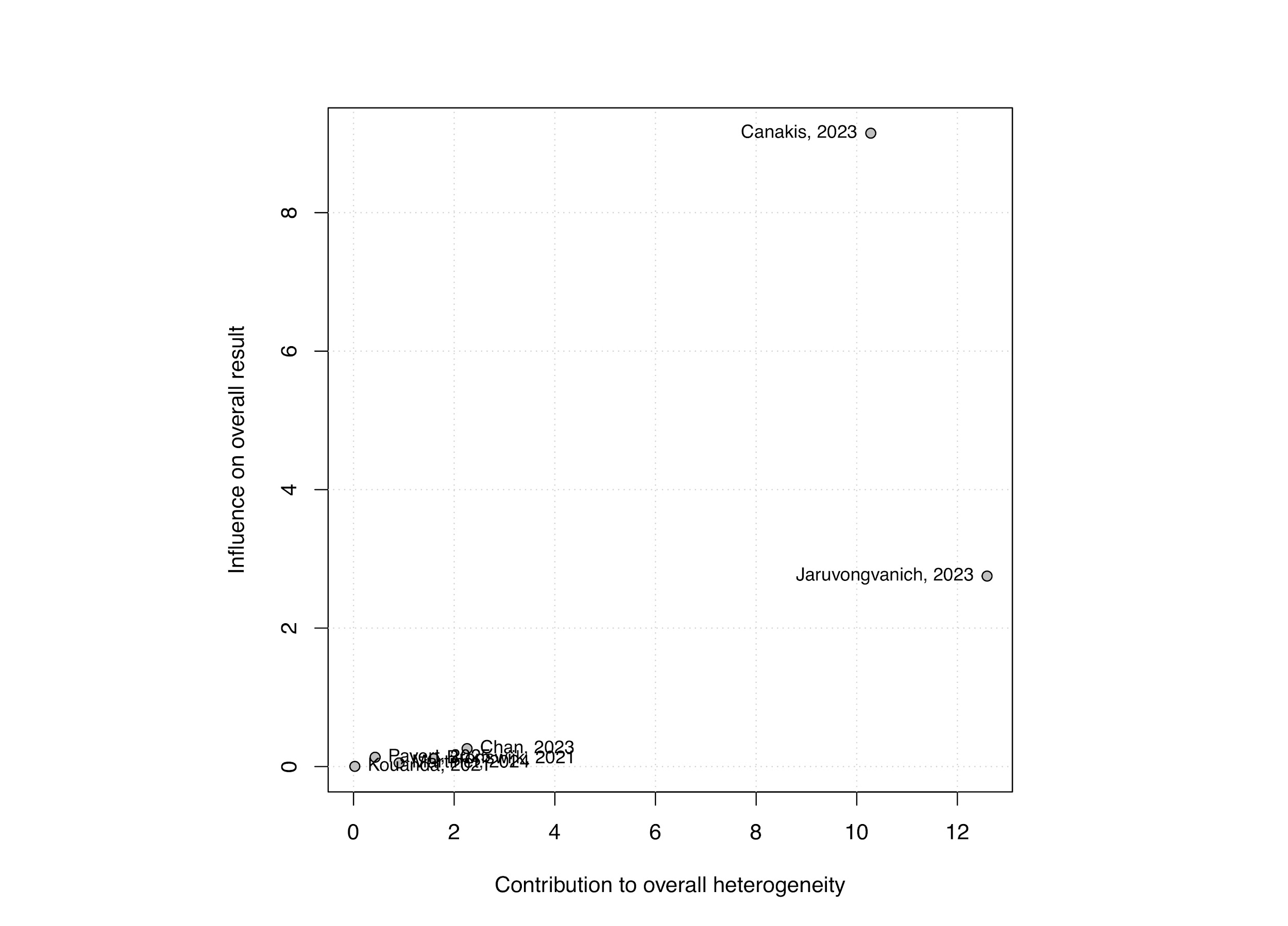


**Supplementary Figure 23.** Baujat plot analysis of length of hospital stay comparing EUS-GE and SGJ


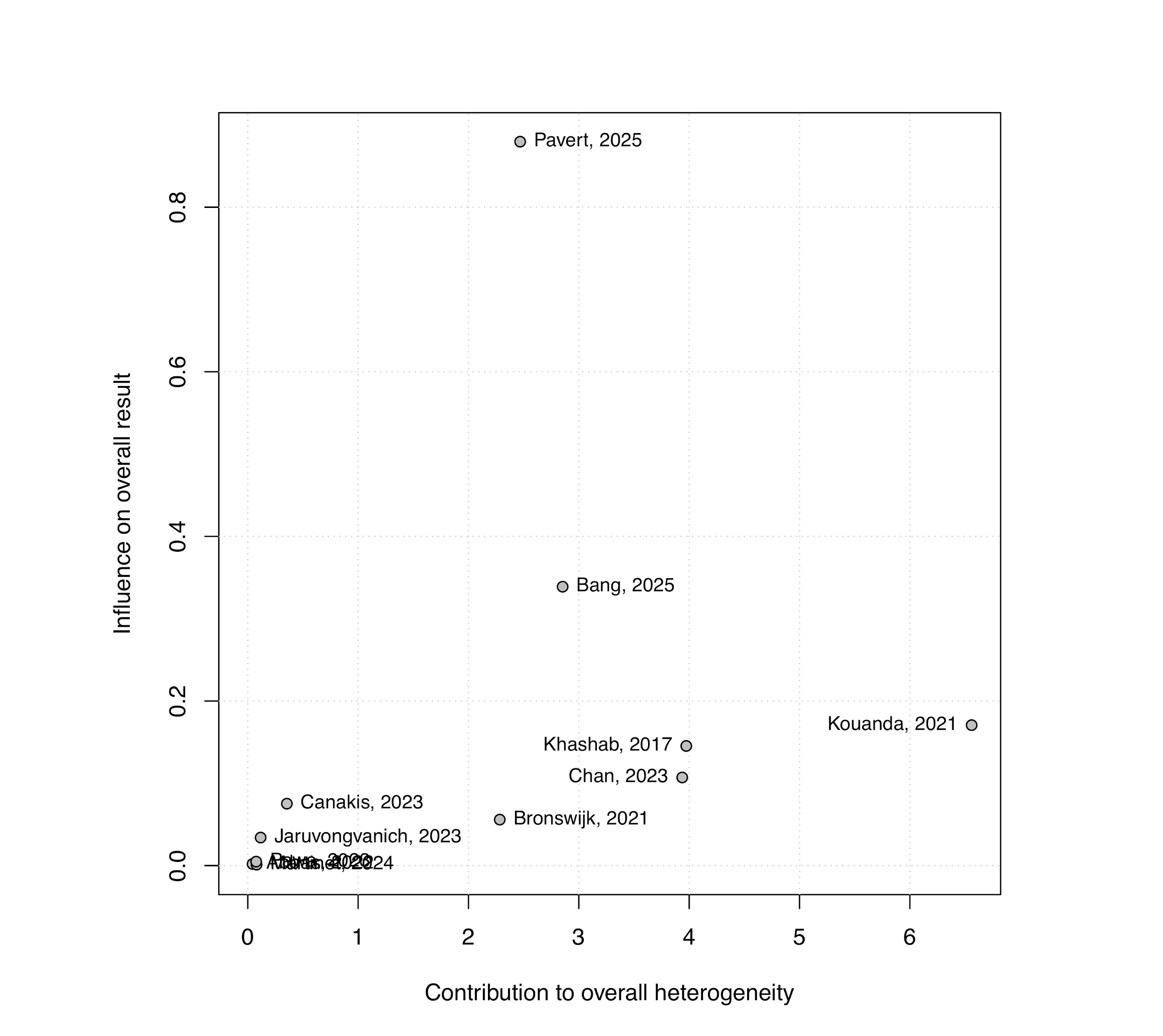


**Supplementary Figure 24.** Baujat plot analysis of operative time comparing EUS-GE and SGJ


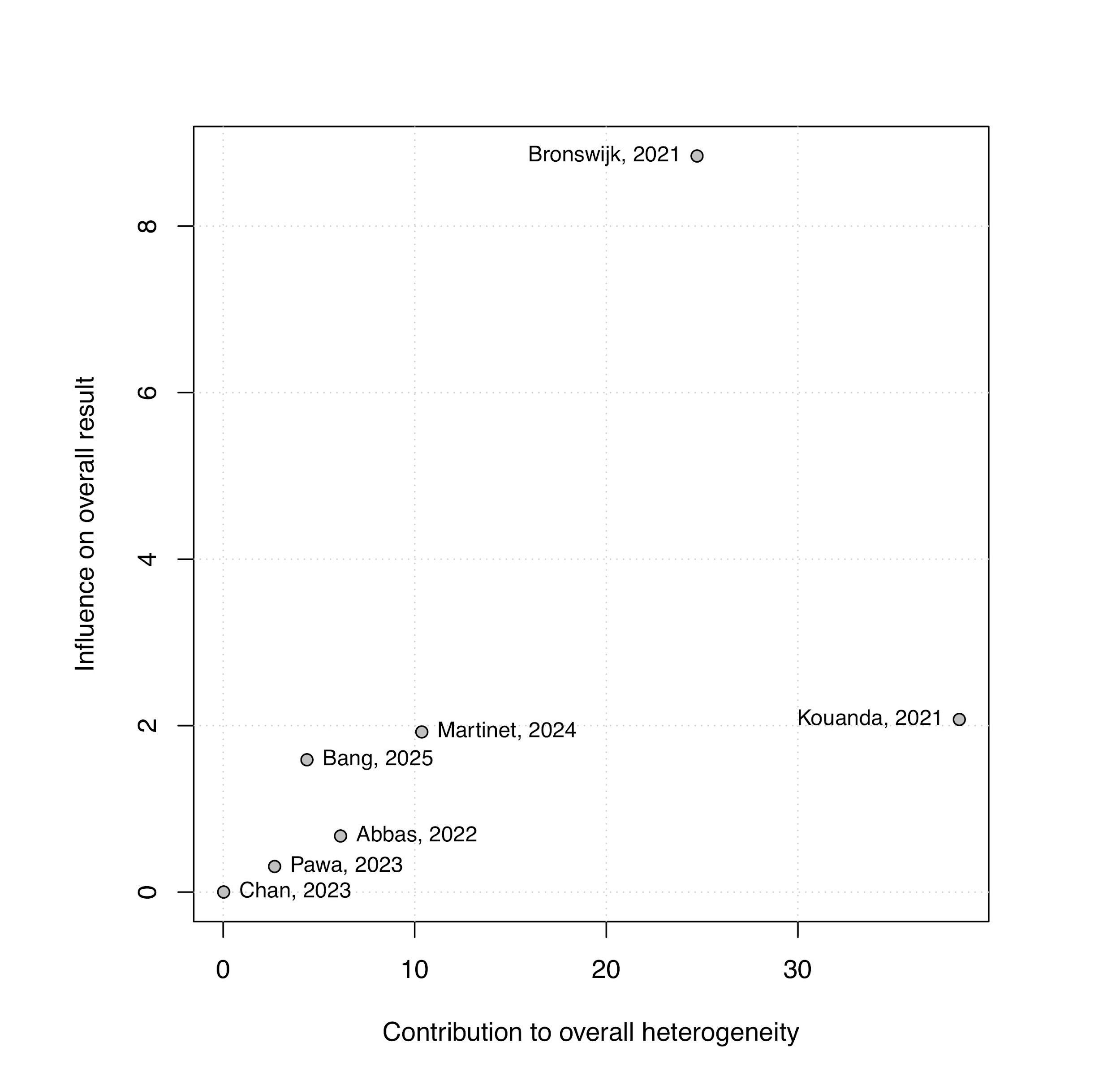


**Supplementary Figure 25.** Baujat plot analysis of postoperative complications comparing EUS-GE and enteral stenting


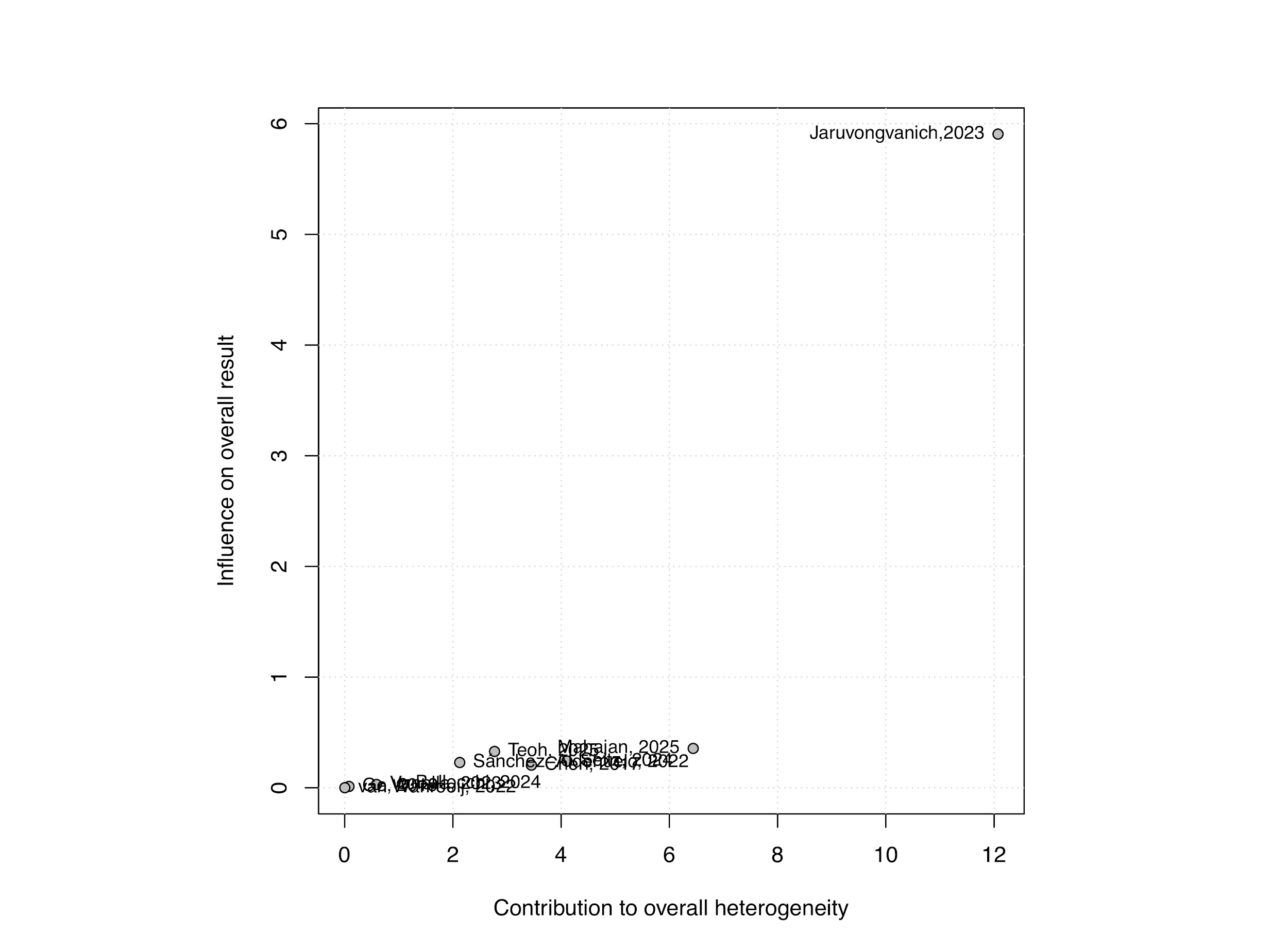


**Supplementary Figure 26.** Baujat plot analysis of length of hospital stay comparing EUS-GE and enteral stenting


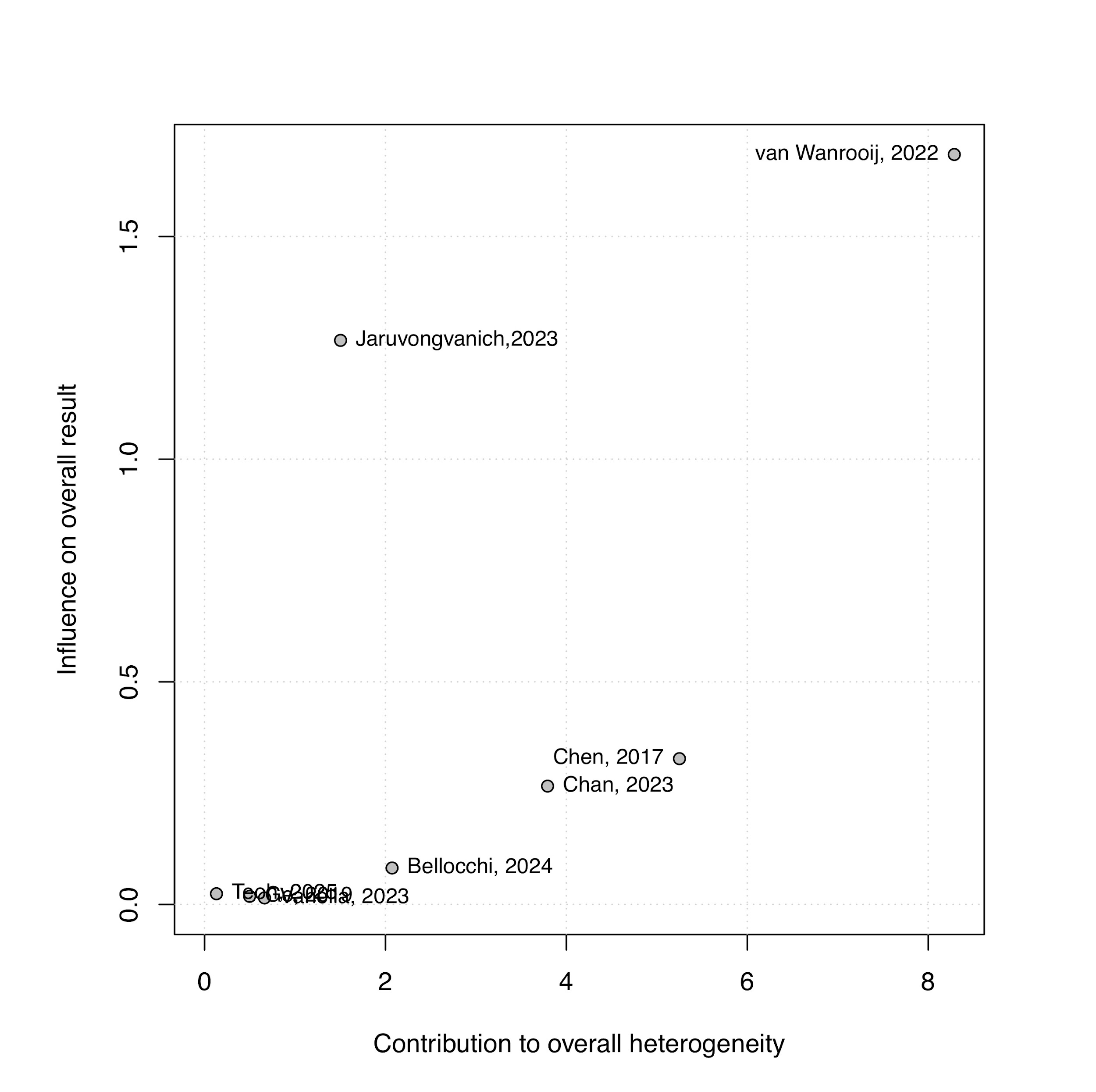


**Supplementary Figure 27.** Funnel plot of technical success comparing EUS-GE and SGJ


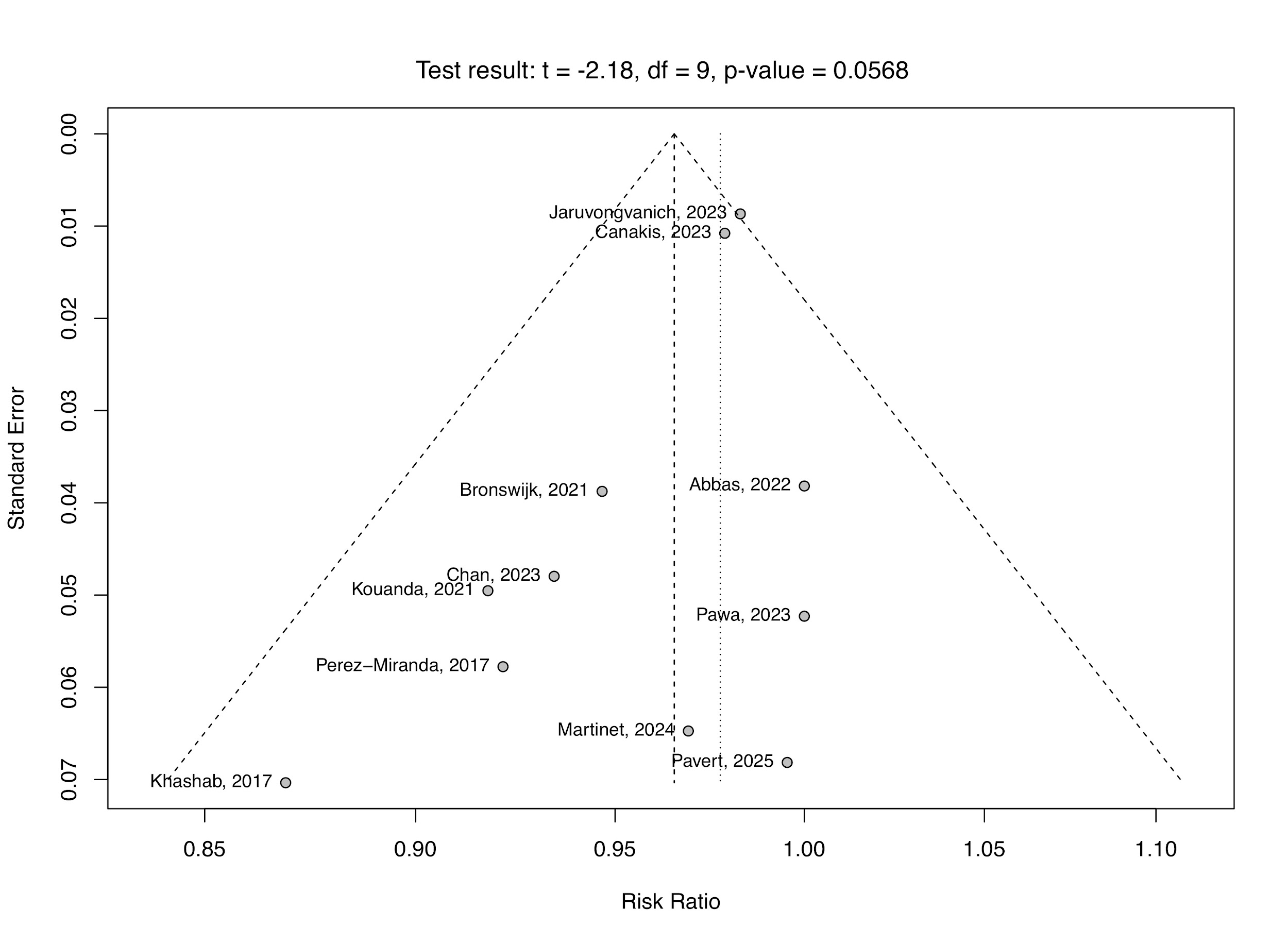


**Supplementary Figure 28.** Funnel plot of clinical success comparing EUS-GE and SGJ


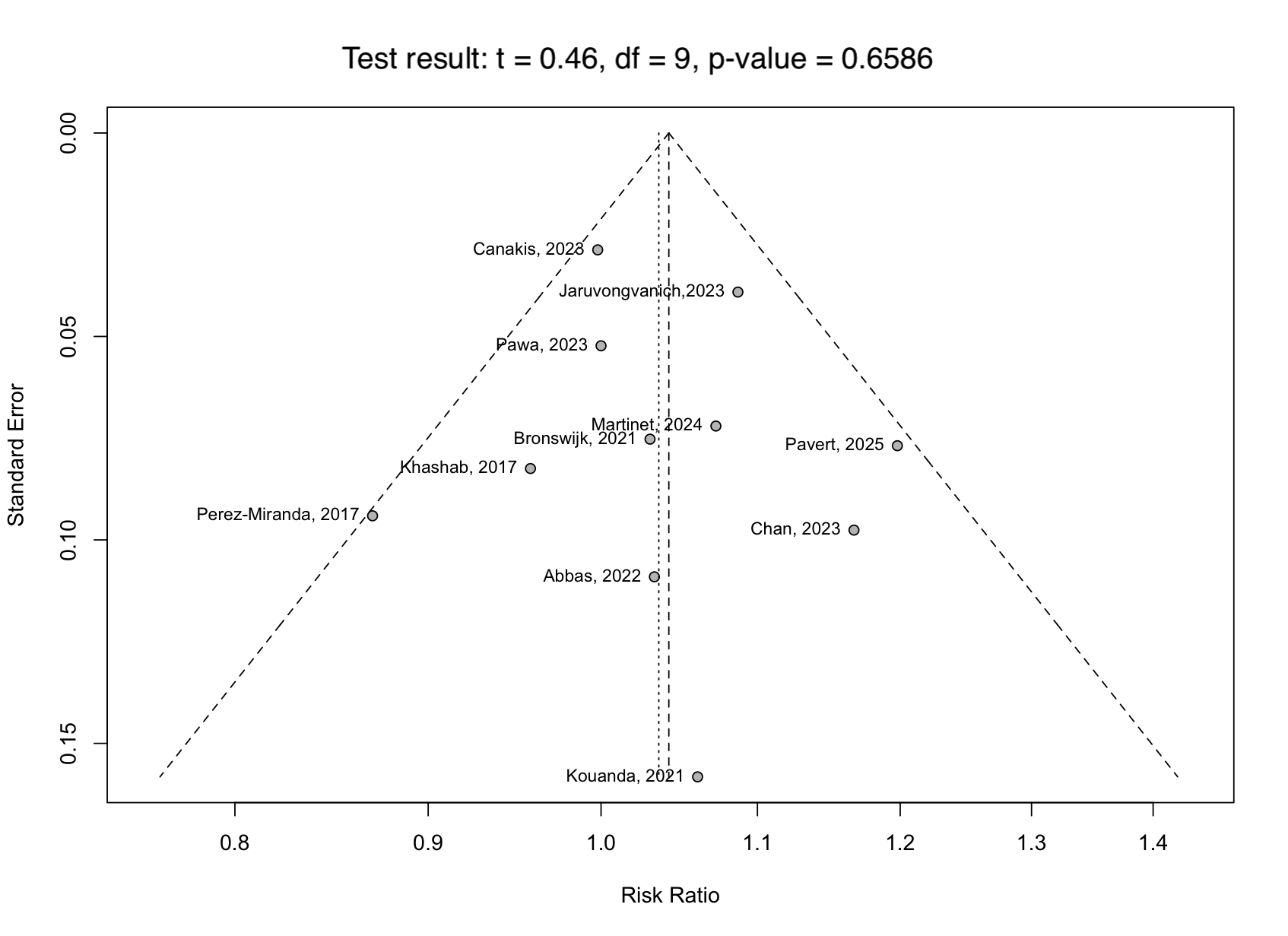


**Supplementary Figure 29.** Funnel plot of technical success comparing enteral stenting


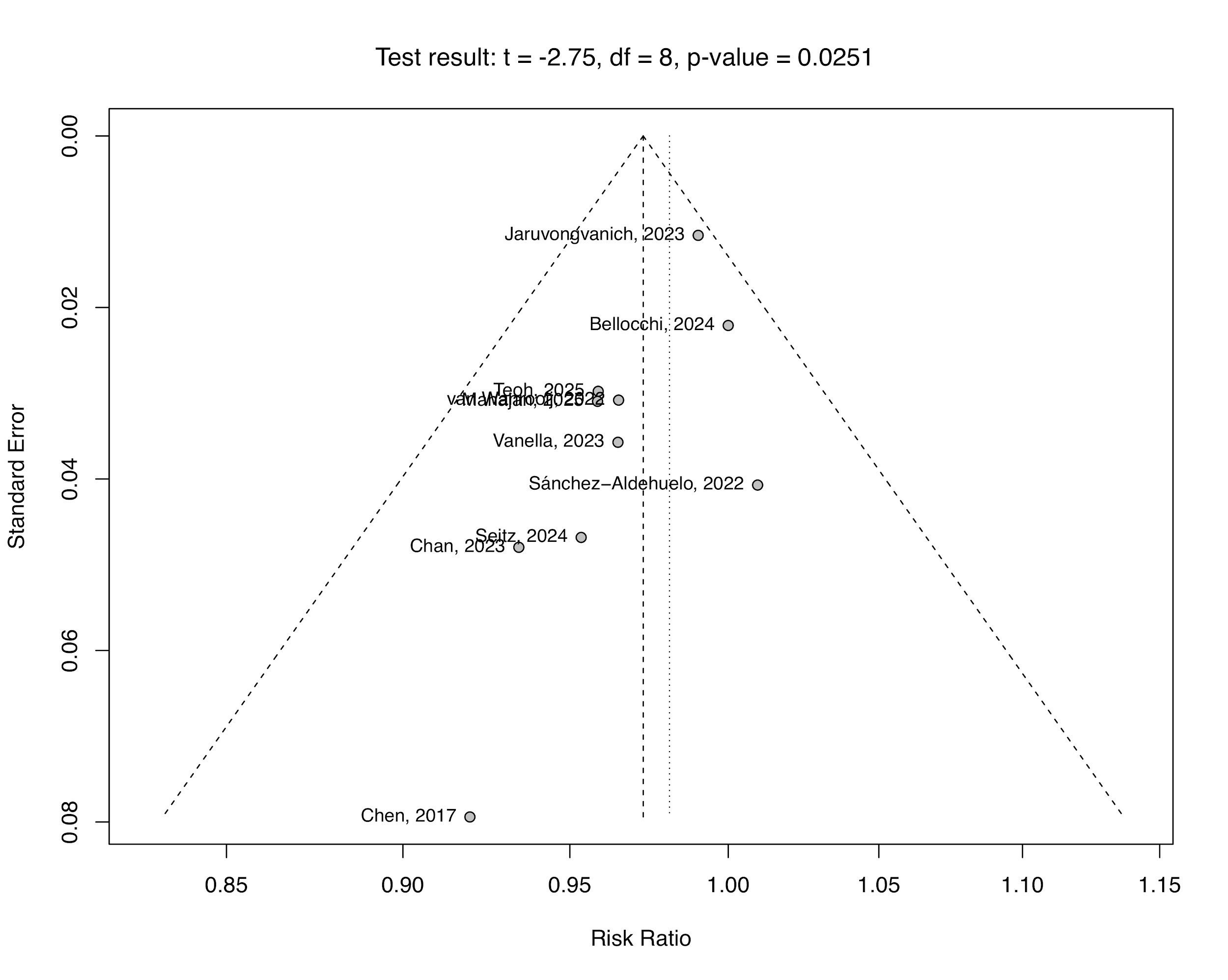


**Supplementary Figure 30.** Funnel plot of clinical success comparing enteral stenting


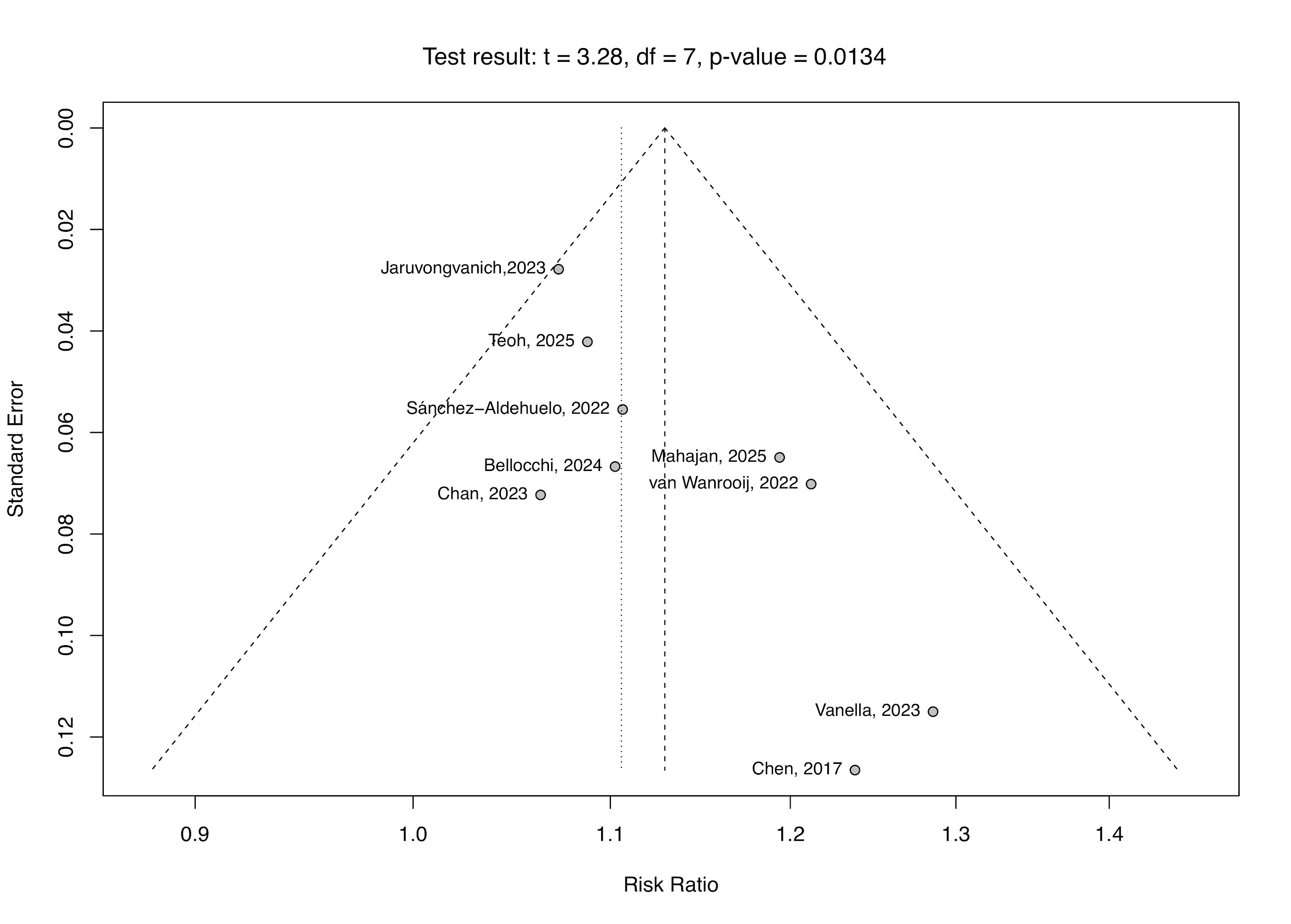


**Supplementary Figure 31.** TSA comparing EUS-GE with SGJ for overall postoperative complications (all-grade Clavien-Dindo)


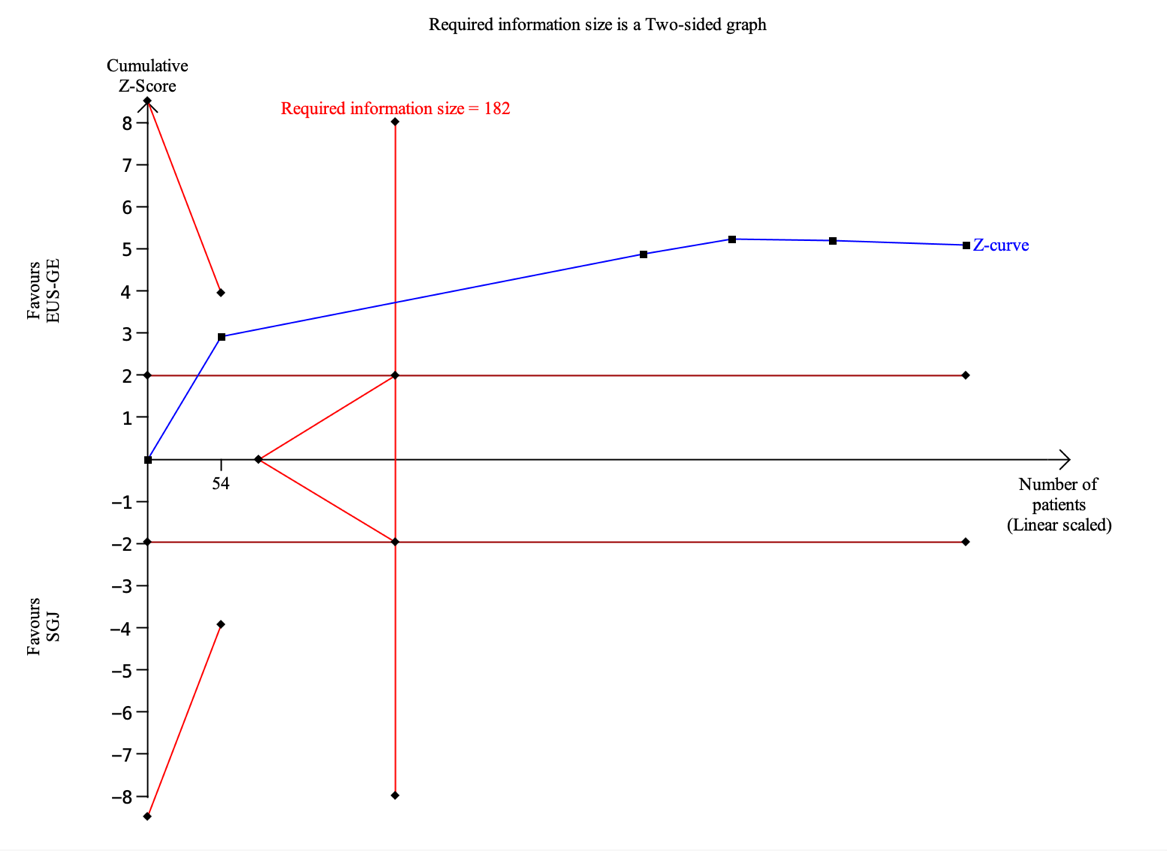


**Supplementary Figure 32.** TSA comparing EUS-GE with enteral stenting for reintervention


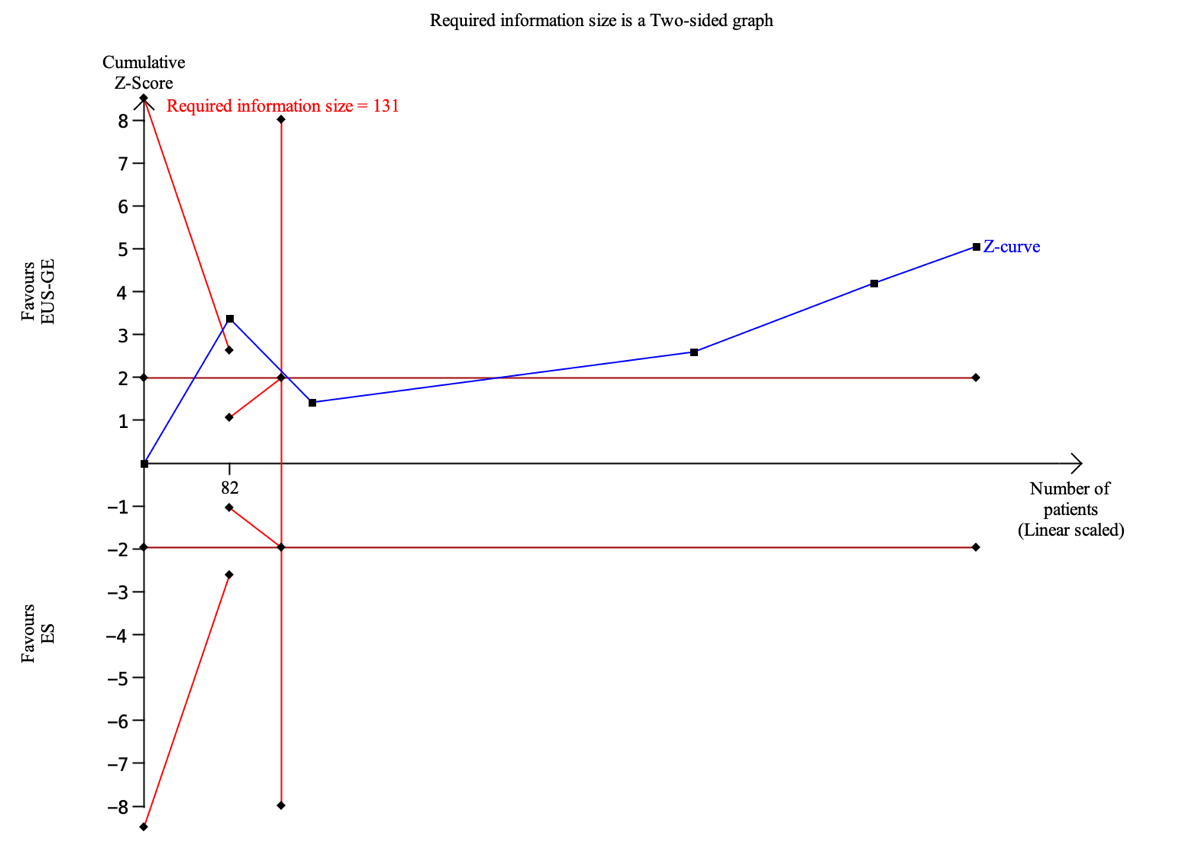

Supplement: Supplementary file 1 — (DOCX 10958 KB) [file 464_2026_12864_MOESM1_ESM.docx]
